# Nanobioprospecting of photoautotrophs for the fabrication of quantum dots: mechanism and applications

**DOI:** 10.3389/fchem.2024.1458804

**Published:** 2024-10-15

**Authors:** Pranav Pandya, Thomas J. Webster, Sougata Ghosh

**Affiliations:** ^1^ Department of Microbiology, School of Science, RK University, Rajkot, Gujarat, India; ^2^ School of Health Sciences and Biomedical Engineering, Hebei University of Technology, Tianjin, China; ^3^ School of Engineering, Saveetha University, Chennai, India; ^4^ Materials Program, Federal University of Piaui, Teresina, Brazil; ^5^ Department of Physics, Faculty of Science, Kasetsart University, Bangkok, Thailand

**Keywords:** quantum dots, algae, plant, mechanism, application, fluorescent probe, biocompatible

## Abstract

Quantum dots (QDs), also known as nanoparticle-based fluorescent probes, are luminescent semiconductor particles with a size range of 2–20 nm. The unique optical and electronic capabilities of QDs have led to expanded applications in several fields such as optoelectronics, transistors, sensors, photodetection, catalysis, and medicine. The distinct quantum effects of nanocrystals can be controlled by changing their sizes and shapes using a variety of top-down and bottom-up tactics. QDs were traditionally fabricated using complex, expensive, toxic, and aggressive chemical techniques, which limited their application in a variety of disciplines. A unique approach for the biosynthesis of nanomaterials has been devised, which employs living organisms in the synthesis process and adheres to green chemistry principles. Biogenic QDs have favorable physicochemical features, biocompatibility, and fewer cytotoxic effects as a result of using natural biomolecules and enzymatic processes for mineralization, detoxification, and nucleation of metals and nonmetals to synthesize QDs. This is the first comprehensive review of its kind that highlights the synthesis of several doped and undoped QDs, including graphene QDs, carbon dots, silicon QDs, N/S-CDs, silver-CDs, cadmium-selenium QDs, and zinc oxide QDs, exclusively using photoautotrophic algae and plants. The different plausible mechanisms behind phyco- and phyto-fabrication of QDs are also discussed in detail along with their applications that include detection of organic and inorganic compounds, degradation of hazardous dyes, free radical scavenging, antimicrobial activity, cytotoxicity and bioimaging. Thus, this review aims to give valuable insights for the rational fabrication of photoluminescent nanomaterials with tunable structural and functional properties.

## 1 Introduction

A class of nanomaterials composed of IV, II–VI, IV–VI or III–V elements formed into tiny bright fluorescent particles known as quantum dots (QDs) has found diverse numerous applications in therapeutics, bioimaging and sensing (Kumar and Kumar, 2013; Demchenko and Dekaliuk, 2013). These QDs are minute spots of matter which are concentrated on a solitary point comprised of trapped electricity. The diameter of these nanoscale semiconductor materials range from 2 to 12 nm (Jin et al., 2023). Different types of QDs may be composed of elemental carbon, silicon, graphene, cadmium, and others. The internal energy of QDs is dependent on the nature of the precursor material used during the fabrication process while their optical properties are mainly affected by the size of the dots themselves (Bentolila, 2015).

QDs are fabricated by various methods like the arc discharge method, acidic oxidation, laser ablation, microwave pyrolysis, etc. QDs fabricated either by physical or chemical methods require high temperature, sophisticated instruments, vacuum conditions, energy, and chemical additives. However, these methods pose a significant threat to the environment owing to the toxic effect of the chemicals that are often attached to the fabricated QDs (Ramanujam and Sundrarajan, 2014; Jain et al., 2018). This in turn reduces the biocompatibility of the QDs. Hence, more recently, researchers have focused on the biogenic fabrication of QDs using microbes and plants. This biogenic synthesis of nanoparticles is rapid, eco-friendly as well as inexpensive (Salem et al., 2015; Mangalampalli et al., 2018; Bloch et al., 2024).

Phycogenic QDs refer to an algae mediated synthesis where the algal metabolites (such as carbohydrates, fats, oil, carotene, chlorophylls, phycobilins, phycocyanin, phycoerythrin, xanthophyll, polyphenols, polyunsaturated fatty acids, tocopherols, and vitamins), have a tremendous scope to synthesize QDs as well as stabilize them (Mukherjee et al., 2021). Algal species like *Chlorella pyrenoidosa*, *Dunaliella salina*, *Sargassum horneri*, *Nannochloropsis*, etc. can be used for the extracellular and/or intracellular synthesis of QDs which is elaborated in Section 2. Biogenic QDs have diverse applications such as bioimaging, sensing, photocatalysis, and energy storage (Liu et al., 2017; Zhou et al., 2021; Ahmadian-Farad-Fini et al., 2021).

Phytogenic QDs refer to a plant mediated synthesis where different biomolecules present in the extracts of seeds, fruits, flowers, peels, leaves, roots, and vegetables (Sahu et al., 2012; De and Karak, 2013; Mondal and Srivastava, 2018), monosaccharides as well as polysaccharides (Peng and Travas-Sejdic, 2009; Zhu et al., 2009; Tang et al., 2012; Shi et al., 2016), proteins and amino acids (Jiang et al., 2012; Huang et al., 2014; Xu H. V. et al., 2018a), nucleic acids (Guo et al., 2013; Li et al., 2015; Song et al., 2015) and biomass as well as their wastes are used for the fabrication of luminescent QDs (Huang et al., 2017; Du et al., 2014; Jones et al., 2017). Plants such as *Aloe vera*, *Camellia sinensis*, *Catharanthus roseus*, *Mangifera indica*, *Phoenix dactylifera* and others can be used for QD synthesis which is discussed in detail in Section 3.

The present review gives elaborate information about the fabrication of QDs by the use of algae and plants along with the mechanism of their formation and various applications.

## 2 Phycogenic quantum dots

Several algae are reported to synthesize pure as well as metal doped or hybrid QDs that are listed in Table 1. This section gives an elaborate overview on the process of synthesizing QDs using algae. Further, it emphasizes the physical and chemical properties of phycogenic QDs.

**TABLE 1:** Phycogenic QDs and their applications.

Name of algae	Type of quantum dots	Size (nm)	Shape	Activity/application	Reference
*Acutodesmus obliquus*	CDs	1.2 to 2.2 (carbohydrate-derived) and 1.5 to 6.6 (lipid-derived)	-	-	Gusain et al. (2021)
Brown algae	N-CDs	13.8	-	Fe^3+^ sensing	Liu et al. (2015)
*Chlorella pyrenoidosa* and *Scenedesmus obliquus*	CdSe QDs	4.15 ± 0.05	spherical	imatinib detection	Zhang et al. (2018)
*Chlorella pyrenoidosa*	CDs	4.5	spherical	fluorescent ink	Guo et al. (2021)
*Chlorella pyrenoidosa*	CDs	<20	sub-globular	Fe^3+^ sensing	Zhang et al. (2022a)
*Chlorella*	CDs	5	spherical	cytotoxicity	Dong et al. (2021)
*Chondrococcus hornemanni*	CD-Ag NC	1–4	spherical	larvicidal, antibacterial and anticancer	Suresh et al. (2023)
*Cladophora rupestris*	CDs	10.0 ± 3.74 nm (from RC precursor) and 76.9 ± 73.1 nm (from CNC precursor)	spherical	solar cell sensitization	Ana and Camacho (2019)
*Cladophora vagabunda*	FCNPs	42.78	-	antibacterial and cytotoxicity	Calangian et al. (2018)
*Dunaliella salina*	CNDs	3–3.5	-	sun protection filter	Chatzimitakos et al. (2020)
*Dunaliella salina*	N/S-CDs	3.2	spherical	Fe(III) detection, photocatalytic degradation, antioxidant and bioimaging	Li et al. (2019a)
*Dunaliella salina*	CDs	4.7	spherical	Hg (II) and Cr (VI) sensing, cytotoxicity, and cell imaging	Singh et al. (2019)
*Enteromorpha prolifera*	CDs	3.6	spherical	antioxidant and cytotoxic effect	Wang et al. (2019)
*Enteromorpha prolifera*	CNDs	4–8	spherical	cell imaging, cytotoxicity, and Fe^3+^ sensing	Xu et al. (2017)
Eutrophic algal bloom	CDs	8.5 ± 5.6	quasi-spherical	cytotoxicity and *in vitro* imaging	Ramanan et al. (2016)
*Halimeda opuntia*	CQDs	<5	spherical	energy storage	Al-Ghamdi et al. (2023)
*Leathesia difformis*	CNDs	17–57	quasi-spherical	-	Kim et al. (2022a)
Microalgal biochar	CDs	-	-	Pb^2+^, Cu^2+^, Cd^2+^, and Ni^2+^ sensing	Placido et al. (2019)
Microalgal powder	CQDs	1–8	spherical	cytotoxicity and cellular imaging	Guo et al. (2017)
*Nannochloropsis*	N/S-CDs	4	spherical	plant tissue imaging	Zhang et al. (2017)
*Pectinodesmus* sp. strain PHM3	CQDs	67	spherical	anticancer	Amjad et al. (2019)
*Sargassum horneri*	CNDs-ZnO	70–90	spherical	antibacterial and antifungal	Kim et al. (2022b)
*Thalassiosira rotula*	SiQDs	2	spherical	phytophysiological effect on cucumber seedling	Li et al. (2019b)

### 2.1 Carbon quantum dots

Carbon dots (CDs) are novel zero-dimensional carbon nanomaterials with sp^2^/sp^3^ hybrid carbon nuclei. They have abundant hydroxyl or carboxyl functional groups. Gusain et al. (2021) fabricated carbon dots (CDs) by using lipids and carbohydrates of microalgae *Acutodesmus obliquus*. The algal species was cultivated in blue-green 11 medium (BG-11) under average intensity of light and temperature (25°C ± 5°C) followed by harvesting of cells via centrifugation, washing, and lyophilization. The carbohydrates from the algal powder were extracted by adding 0.15 g powder in 50 mL H_2_SO_4_ (4%) followed by microwaving the solution at 125°C for 30 min. In the carbohydrate-mediated synthesis of CDs, the extracted solution containing carbohydrates was removed and subjected to centrifugation for 10 min at 6,000 rpm followed by the addition of 20 mL supernatant in urea solution. The reaction mixture was stirred for 10 min and was subjected to microwave heating for 60 min at 180°C. The resulting brown color of the solution suggested CDs fabrication. Similarly, the lipids were extracted by mixing 0.25 g microalgal powder in 50 mL water followed by its addition in a 2:1 mixture of chloroform and methanol. The reaction mixture was stirred and microwaved at 100°C for 10 min followed by filtration of the sample. The fabrication of CDs from lipids was initiated by pouring hexane into the lipid filtrate followed by the addition of an urea solution to it. The reaction mixture was further heated at 220°C in the microwave which resulted in the formation of two different layers (hexane and aqueous layer). UV-Vis analysis identified the first excitation peaks at 354 nm (for carbohydrate-derived CDs) and 343 nm (for lipid-derived CDs) which formed emission peaks at 430 and 422 nm, respectively. The second excitation peaks were observed at 630 and 624 nm for carbohydrate-derived and lipid-derived CDs, respectively. TEM analysis revealed the size of particles ranging from 1.2 to 2.2 nm for carbohydrate-derived CDs and 1.5–6.6 nm for lipid-derived CDs.

Liu et al. (2015) introduced a novel method for the fabrication of nitrogen-doped carbon dots (N-CDs) by employing alginic acid (AA) derived from brown algae and further studied their use in sensing metal ions. Initially, 0.15 g AA was mixed in 5 mL water supplemented with 0.3 mL ethanediamine (EDA) followed by 1 h of stirring. The reaction mixture was further heat treated for 6 h at 180°C and afterwards was cooled to room temperature. The resulting dark brown colored solution was subjected to 30 min of sonication followed by 30 min of centrifugation (7,500 rpm). The supernatant obtained was dialyzed for 2 days in order to obtain the highly pure CDs. Quantum yield (QY) of the synthesized CDs was about 18.9% higher than that of undoped CDs (non-nitrogen). UV-Vis analysis identified absorption peaks at 292 nm (aromatic π-π* transition of carbon core) and 362 nm (n-π* transition). PL analysis demonstrated the emission at 450 nm upon excitation with a 380 nm wavelength. The diluted aqueous CDs showed light yellow color under natural light whereas it exhibited vivid cyan fluorescence under UV exposure (365 nm). FTIR analysis determined absorbance peaks at 3,505, 3,290, 1,667–1,593, and 1,490–1,272 cm^−1^ that were attributed to the stretching vibrations of O-H, N-H, amide linkages, and C=C, C=N, and C=C-O groups, respectively. The particles were smaller in size with a mean diameter of 13.8 nm. No significant damage in PL intensity was observed at pH 2–5. Even after 8 cycles from pH 3 to 9, no noticeable perturbation was observed in the pH-switching PL ability of the N-CDs.

Guo et al. (2021) carried out the fabrication of nitrogen self-doped CDs (N-CDs) via *C. pyrenoidosa* and its use as a fluorescent ink. The particles were initially synthesized by mixing 1 g algal powder in 50 mL water followed by stirring at 200 rpm, 170°C–210°C and 1–3 h of incubation time. The reaction mixture was further cooled to room temperature and was centrifuged for 6 min at 10,000 rpm. The supernatant obtained was dialyzed for 48 h in order to obtain a suspension of CDs which was further lyophilized (48 h) to form a powder. The solid residue was further dried for 24 h at 105°C. With an increase in temperature from 170°C to 210°C after 1 h of incubation, the hike in the liquid phase yield was noted from 76% to 89% whereas the decrease in solid phase yield was noted from 24% to 11%. Similarly, after 3 h of incubation at 200°C, enhancement in the liquid phase from 76% to 89% was noted and a reduction in solid phase yield was noted from 24% to 11%. UV-Vis analysis determined the absorption peaks at 220 and 280 nm (π-π* transition of C=C) and a broad peak at 330 nm (n-π* transition of C=O). PL analysis identified shifting in emission peaks from 360 nm to 527 nm (CD-170), 373 nm–527 nm (CD-190), and 425 nm–527 nm (CD-120) with an increase in excitation wavelength from 280 nm to 460 nm. TEM images revealed a spherical morphology of the particles having a uniformly dispersed nature with sizes ranging from 3 to 6.5 nm. The mean size of the particles was around 4.5 nm. The height of the particles was around 5 nm. FTIR analysis identified absorbance peaks that were attributed to the stretching vibrations of C=O (1,642 cm^−1^), N-H (1,530 cm^−1^), C-N (1,456 cm^−1^), and O-H or N-H (3,280 cm^−1^). XPS analysis confirmed the existence of C (61.6%–69.2%), N (4.1%–11.9%), and O (34.3%–18.9%) in the particles. The QY of CD-170, CD-190, and CD-210 were around 1.79% ± 0.026%, 1.81% ± 0.025%, and 2.31% ± 0.036%, respectively. The particles under UV exposure (365 nm) exhibited blue-white fluorescence (CD-170), blue-grey fluorescence (CD-180), grey fluorescence (CD-190), and no fluorescence (CD-200 and CD-210).

Zhang et al. (2022a) fabricated CDs via the hydrothermal treatment of *C. pyrenoidosa* and further analyzed the detection ability for the Fe^3+^ ion. The synthesis of particles was initiated by mixing the algal powder in water in a liquid to solid ratio of 5:1 to 30:1 followed by the addition of nitrogen gas and autoclaving at 150°C–270°C for 2–10 h. The product formed was cooled to room temperature and centrifuged for 20 min at 8,000 rpm to collect the supernatant. The dialysis of the supernatant was followed by freeze-drying. The pellets were treated with ethyl acetate and were further dried for 12 h at 80°C. The optimum parameters to obtain a better yield of CDs were a 20:1 liquid-to-solid ratio, 230°C reaction temperature, and 6 h of reaction time. At optimum conditions, the amount of C, H, N, S, and O were 56.8, 6.3, 8.1, 1.2, and 27.6%, respectively, which suggested successful doping of N and S on the CDs. The highest yield of 7.2% was observed for the particles under optimum conditions. UV-Vis analysis identified the absorption in the range of 200–500 nm. The particles also exhibited a red-shift of peak to 238 nm whereas n-π* transition of C=O of CDs was revealed from a peak at 292 nm. The peak at 365 nm was attributed to the n-π* transition of N/P/O-containing groups in CDs. At 360, 340, and 300 nm excitation, the CDs showed PL intensities at 440, 410 and 410 nm, respectively. The red-shifting of peaks of emission was from 440 nm to 550 while for the excitation intensity the same was between 300 and 500 nm. The particles emitted blue fluorescence under UV exposure (365 nm). FTIR spectra revealed absorbance peaks that were attributed to the stretching of asymmetric N-H (3,008 cm^−1^), symmetric N-H (1,522 cm^−1^), C-O (1,300–1,000 cm^−1^), and SO_4_^2-^ (1,119, 701, and 620 cm^−1^). The size of CDs was less than 20 nm with a sub-globular structure. EDS analysis identified the occurrence of O, K, P, S, and N elements in the CDs which suggested their direct doping on CDs.

**FIGURE 1:**
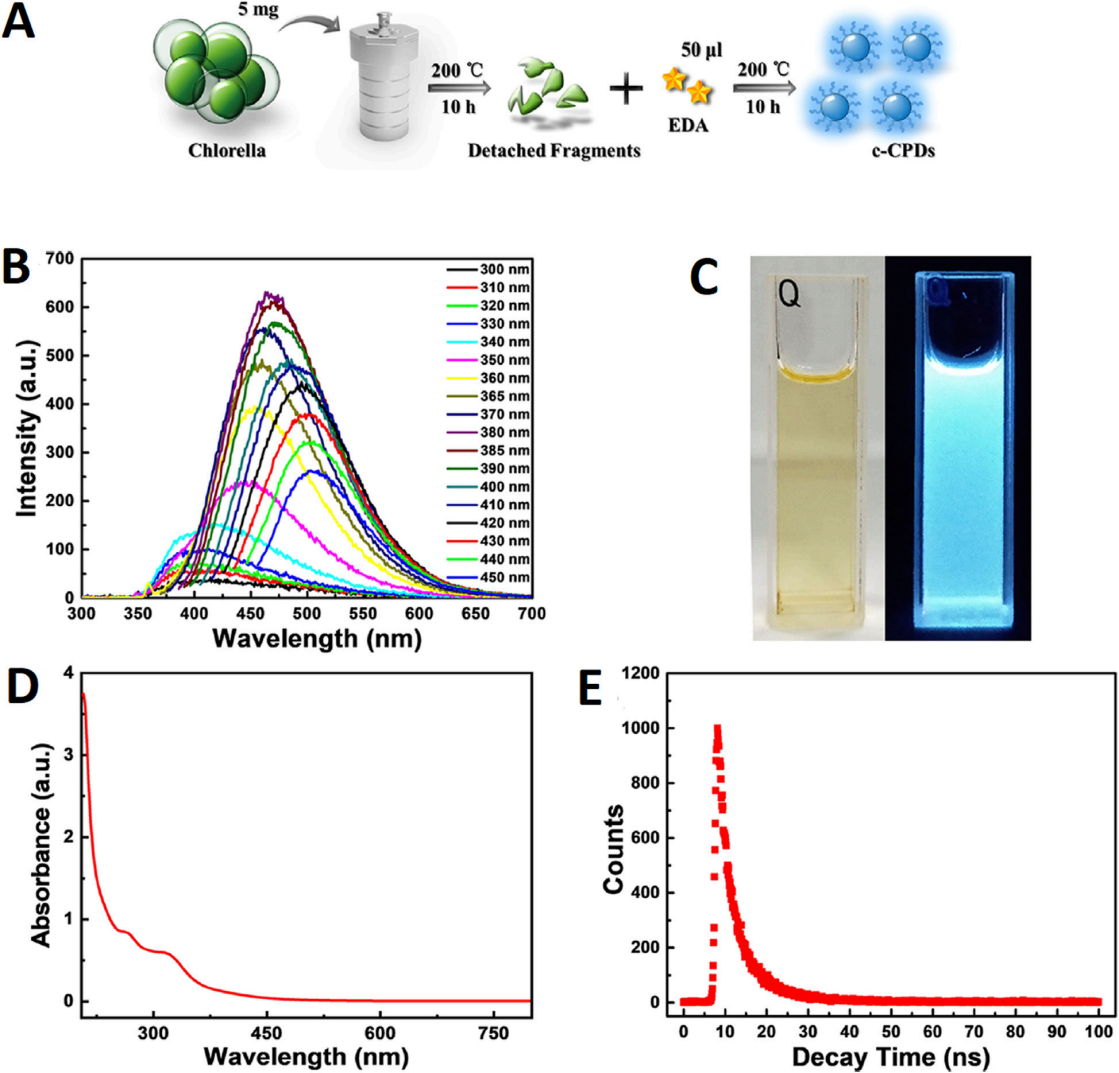
**(A)** Schematic illustration of the experimental procedure; **(B)** Fluorescence emission spectra of c-CPDs; **(C)** the photographs of c-CPDs under visible (left) and UV (right) light (365 nm); **(D)** UV−vis absorption spectra of c-CPDs; and **(E)** the fluorescence decay curve of the c-CPDs. Reprinted with permission from Dong et al. (2021). Facile hydrothermal synthesis of *Chlorella*-derived environmentally friendly fluorescent carbon dots for differentiation of living and dead *Chlorella*. *ACS Appl. Bio Mater.* 4 (4), 3,697–3,705. doi:10.1021/acsabm.1c00178. Copyright^©^ 2021 American Chemical Society.

In another study, Dong et al. (2021) fabricated fluorescent CDs using *Chlorella* as schematically depicted in Figure 1. The synthesis was carried out by implementing a hydrothermal method. Firstly, the algal species were cultured in BG-11 medium at 25°C under the illumination of light followed by centrifugation of the medium in order to obtain the moist precipitates of alga. About 5 mg of alga (dry weight) was added to 10 mL of water which was further heated at 200°C for around 10 h. After heating, the reactor was allowed to cool down to room temperature followed by the addition of 50 µL ethanediamine. The reaction mixture was further heated at 200°C for about 10 h. After cooling down to room temperature, the pale-yellow colored solution was filtered and placed in a dark condition at ambient temperature. The particles exhibited a transparent nature with a pale yellow color under natural light, whereas, they illuminated bright blue colored fluorescenced under UV exposure (365 nm). At 380 nm excitation intensity, the particles displayed 460 nm of emission wavelength. With the increase in excitation wavelength from 330 to 450 nm, the fluorescence emission peak showed a red shift from 400 nm to 510 nm. The quantum yield of the particles was 10.6% in solution excited with 380 nm of light. UV-Vis analysis showed absorption peaks for the particles at 260 nm (π-π* transition of aromatic C=C bonds) and 315 nm (n-π* transition of C=O bonds). At 375 nm, the average lifespan of the particles was about 5.79 ns. FTIR spectra identified the absorbance that was attributed to the vibrational stretching of O-H or N-H (3,423 cm^−1^), C-H (3,193 and 3,010 cm^−1^), C=O (1,635 cm^−1^), C=C (1,589 cm^−1^), N-H (1,512 cm^−1^), C-N (1,338 cm^−1^), C-O (1,033 cm^−1^), and C-S (665 cm^−1^). TEM images revealed agglomerated particles exhibiting a spherical morphology with sizes ranging from 3.5 to 6.5 nm and a mean particle size of 5 nm. With an increase in dosage concentration of algal species, the intensity of the peak at 387 nm showed reduction whereas that of 455 nm showed a significant increase.

Suresh et al. (2023) used *Chondrococcus hornemanni* for the fabrication of a CD-Ag nanocomposite (CD-Ag NC) via a hydrothermal process and further studied its role in larvicidal, antibacterial and anticancer applications. The fabrication process began by mixing 2 g algal powder in 100 mL water followed by heating at 60°C for 1 h. The extract was filtered and 50 mL of the filtrate was mixed with 0.169 g AgNO_3_ and was stirred for 10 min at room temperature. After autoclaving the reaction mixture for 4 h at 180°C, the reaction was completed and was further allowed to cool up to room temperature. The resulting NC were dark brown in color. The particles were further centrifuged at 12,000 rpm for 10 min and the water was evaporated at room temperature which resulted in the formation of NCs. UV-Vis analysis identified absorption peaks at 304 nm (CDs) and 402 nm (CD-Ag NC). FTIR analysis revealed absorbance peaks at 3,505, 2,929, 2,353, 1732, 1,646, 1,384, 1,018, 671, and 540 cm^−1^ in the case of NC and at 3,610, 2027, 1,635, 1,252, 721, and 531 cm^−1^ in the case of CDs. XRD analysis identified the face-centered cubic crystalline structure of the particles and TEM images identified the uniformly dispersed spherical-shaped particles 1–4 nm size. The particles were mainly comprised of 2.12% C, 47.92% Ag, and 49.96% Cu without any impurities. The zeta potential of the fabricated NCs was −22.2 mV.

Ana and Camacho (2019) carried out the synthesis of CDs by the use of cellulose extracted from *Cladophora rupestris* and further studied its potential application as a photosensitizer in solar cells. The fabrication process began by mixing 2 g algal cellulose (raw cellulose-RC or cellulose nanocrystals-CNC) in 40 mL water followed by sonication of the mixture for 15 min and autoclaved it at 220°C for 6 h. After cooling, the mixture was subjected to 20 min of centrifugation at 10,000 rpm in order to separate the solid black-colored particles (hydrochar). The yellow-brown colored solution was collected and purified via dialysis and filtration. A yield of around 54.6% ± 2.71% was observed for CDs in the case of the CNC precursor which was higher compared to the CDs synthesized using the RC precursor (37.2% ± 2.76%). UV-Vis spectral studies identified absorption peaks of CDs in the range of 250–300 nm. FTIR analysis revealed absorbance peaks at 3,410, 2,920, 1,627, 1,220 and 1,160, and 985 cm^−1^ which were attributed to the stretching vibrations of hydroxide, C-H, O-H, C-O, and C-H (carbohydrates). The size of the particle was about 5.79 ± 1.60 nm and the particles were spherically shaped. The spherical morphology of the particles was also confirmed from AFM images and the size of the particles was 10.0 ± 3.74 nm (from the RC precursor) and around 76.9 ± 73.1 nm (from the CNC precursor).

Calangian et al. (2018) derived fluorescent carbon nanoparticles (FCNPs) from the algae *Cladophora vagabunda* and studied their antibacterial and cytotoxic activities. The synthesis was carried out by mixing 0.5 g of algal powder in 150 mL of ethanol (40%) followed by calcinating it for 1 h at 200°C. After cooling to room temperature, the resultant solution was centrifuged at 6,000 rpm for 20 min in order to collect the supernatant. The color of the particles was pale green in normal light whereas it emitted red luminescence under UV exposure. UV-Vis spectra identified an absorption peak at 260 nm as well as a shoulder peak at 305 nm contributed by the aromatic π system or n-π* transition of the carbonyl group. The particles showed two emission peaks at 600 and 650 nm upon excitation with a 550 nm wavelength. FTIR analysis identified absorbance peaks attributing to the stretching vibrations of O-H (3,427 cm^−1^), C-H (2,924 cm^−1^), C=C (1,403 cm^−1^), C=C-H (653 cm^−1^), C=O (1700 cm^−1^), C-N (1,208–1,370 cm^−1^), C-O and P-O (1,038–1,170 cm^−1^). The size of the particle as revealed from DLS analysis was around 42.78 nm.

Chatzimitakos et al. (2020) used *D. salina* in the fabrication of carbon nanodots (CNDs) and further studied their application as sun protection filters. The fabrication process was initiated by heating 30 mg algal biomass for 2 h at 250°C followed by cooling it down to room temperature. After the addition of water to the black powder, the mixture was sonicated for 5 min followed by 5 min of centrifugation at 3,500 rpm. The supernatant obtained was filtered and freeze-dried. Around 12.8 mg CND powder was formed (yield = 38.7%) which was further stored in dark conditions at room temperature. UV-Vis spectra revealed an absorption peak at 270 nm (n-π* and π-π* transition of carboxyl -C-O bonds or π-π* transition of aromatic -C=C bonds). The particles showed the highest emission intensity of 470 nm after excitation at 380 nm. FTIR analysis of the CNDs identified absorbance peaks attributed to the stretching vibrations of O-H (3,300 cm^−1^), C-H (2,930 cm^−1^), C=C (1,600 cm^−1^), -COO- (1,420 cm^−1^), C-O (1,137 cm^−1^), and C=O (996 cm^−1^). The mean size of the particles was around 3–3.5 nm.

Li et al. (2019a) fabricated CDs co-doped with nitrogen and sulfur by employing a one-step hydrothermal carbonization approach and *D. salina* as the source and further investigated its ability for Fe(III) detection, photocatalytic dye degradation, antioxidant activity, and bioimaging applications. The algal species was cultured and the cells were further harvested via 4 min of centrifugation at 7,800 rpm followed by washing thrice with water and drying at 60°C. The dried algal biomass was ground to form a fine powder. About 0.2 g powder was mixed in 50 mL water followed by sonication and heating for 5 h at 200°C. The resulting product was filtered and lyophilized. The highest QY of the fabricated particles was 5.93%. UV-Vis analysis identified an absorption peak in the range of 250–310 nm. The particles possessed an excitation peak at 322 nm and an emission peak at 412 nm (fluorescence). The particles were transparent light yellow in color under the exposure of daylight whereas they emitted blue fluorescence under UV exposure (365 nm). The particles possessed a spherical morphology with a size of 3.2 nm as identified from TEM images. Similarly, AFM studies revealed the particles had a height of 3.2–4.0 nm. FTIR analysis identified absorbance peaks attributing to the stretching of O-H and H-bonding by carboxyl groups from peaks at 3,396, 2,961, and 2,929 cm^−1^ as well as that of C=O (1,660 cm^−1^), C-N (1,385 or 1,140 cm^−1^) and C-S (623 cm^−1^). The elemental composition of N/S-CDs included 66.4% carbon, 26.5% oxygen, 5.0% nitrogen, 1.2% sulfur, and 0.9% sodium.

Singh et al. (2019) used *D. salina* biomass for CD fabrication and further studied their role in sensing Hg (II) and Cr (VI) as well as in cytotoxicity and imaging of live cells. Initially, the algal culture was inoculated in 1,500 mL modified Jonson medium (MJM) comprising 0.5 M NaCl followed by incubating under white fluorescent light at 28°C ± 2°C under 16 h of light and 8 h of the dark cycle. After incubation, the algal biomass was collected via 4 min of centrifugation at 4,500 rpm followed by washing. About 6 g of wet biomass was added to 100 mL water and was further autoclaved at 200°C for 3 h followed by cooling down to room temperature. The resulting solution was centrifuged for 15 min at 10,000 rpm and the supernatant collected was purified via a dialysis process. UV-Vis analysis showed absorption peaks at 275 nm (π-π* transition) and at 328 nm (n-π* transition). The particles exhibited excitation peaks at 240 nm (NPs π-π*) and at 358 nm (n-π* transition of surface moieties). At 340 nm excitation, the particles showed an emission at a 415 nm wavelength. It was observed that the particles emitted blue-colored fluorescence under UV exposure (365 nm). FTIR spectra identified the peaks of absorbance that were attributed to the vibrations of -OH, N-H, aromatic-C-H, aliphatic-C-H, amide C=O, N-H bending, -C=C, C-O, C-N/P-N and phosphate at 3,393, 3,272 and 3,063, 2,927, 2,851, 1,668, 1,516, 1,440, 1,379, 1,152, and 697 cm^−1^, respectively. TEM images identified the spherical morphology of the particles with a size ranging from 2 to 8 nm and a mean particle size of 4.7 nm. The crystalline nature of the particles was also confirmed from the selected area (electron) diffraction (SAED) pattern. XRD studies identified the amorphous nature of the particles while the zeta potential was −5.31 mV. The composition of particles revealed from the elemental analysis showed the occurrence of 52.2% carbon, 42.9% oxygen, 2.6% nitrogen, and 2.2% phosphorous.

Wang et al. (2019) employed *Enteromorpha prolifera* for the fabrication of CDs and further implemented them in radical scavenging as well as tested their cytotoxic effects. After the collection of the algal species, the soaking and washing of the sample were fulfilled followed by draining. About 150 g algal biomass was added to 600 mL water followed by 15 min of mixing. The resulting solution was centrifuged for 15 min at 10,000 rpm and 15°C in order to obtain the green paste of algal species from which 10 g paste was heated for 30 min at 200°C. The reaction mixture was further allowed to cool down to room temperature in order to obtain a dark brown colored solution which was further filtered and dialyzed for 24 h to obtain a pure CDs solution. A yield of about 0.75% ± 0.25% was achieved after the fabrication of CDs. UV-Vis spectral studies identified absorption at 282 nm which was attributed to the π-π* transition. The particles emitted blue fluorescence at 450 nm upon excitation with a 380 nm wavelength. FTIR analysis identified the absorbance peaks associated with the stretching of O-H and N-H (3,350 cm^−1^), C-H (2,940 cm^−1^), C=O (1770 cm^−1^), C=C (1,620 cm^−1^), C-N (1,400 cm^−1^), and C-O (1,100 cm^−1^). HR-TEM analysis indicated the uniform dispersity of the spherical-shaped particles with sizes ranging from 2 to 6 nm and a mean particle size being 3.6 nm.

Xu et al. (2017) used biomass derived from *E. prolifera* for the fabrication of sulfur/nitrogen co-doped carbon nanodots (CNDs) which were further investigated for their application for cell imaging, for studying *in vitro* cytotoxic effects as well as sensing of metal ions. The particles were synthesized by employing a one pot hydrothermal method in which about 2 g algae were chopped and fine powder was dissolved in 120 mL water followed by heating of the reaction mixture for 3, 6 or 10 h at 180°C. The particles were further centrifuged for 10 min at 12,000 rpm followed by filtration and washing in ethanol in order to obtain purified CNDs. After the hydrothermal treatment of algal powder, the solution exhibited a yellow or light brown color in natural light whereas they emitted bright blue light under UV exposure. UV-Vis analysis identified the absorption peak at 286 nm that was attributed to the π-π* transition of C=C bonds. The particles showed the highest emission intensity of 450 nm upon excitation with 370 nm. FTIR spectra determined the absorbance associated with the stretching vibrations of O-H (3,417 cm^−1^), C=O and C=C (1,637 cm^−1^), C-H (2,939 and 1,396 cm^−1^), C-O-C (1,398 cm^−1^), and C-S (1,317 cm^−1^). The QY of the particles before and after sedimentation (using ethanol) was around 10.1% and 12.3%, respectively. TEM images revealed the aggregated particles with a size range of 30–140 nm (at 150°C, 3 h). The mean particle size of the fabricated CNDs was around 2.75 ± 0.12 nm. The height of the particles determined from AFM images was in the range of 1.5–3.0 nm. The shape of the particles demonstrated from TEM and AFM images was spherical with the uniform dispersion of particles without notable aggregation. The hydrodynamic size was 4–8 nm while the zeta potential was −2.57 mV. The optimum temperature and incubation time for synthesizing CNDs were 180°C and 6 h, respectively.

Ramanan et al. (2016) carried out the synthesis of CDs using eutrophic algal bloom comprising species belonging to 10 different algal families and further analyzed its cytotoxicity as well as application in *in vitro* imaging. The algal families included in the algal bloom were *Cyanophyceae*, *Bacillariophyceae*, *Chlorophyceae*, and *Eugenophyceae*. The algal biomass was initially washed and dried under low light exposure followed by their conversion into a fine powder. Around 1 g algal powder was added to 20 mL phosphoric acid (40%) followed by heating in a microwave for 5 min and 50 s which resulted in the formation of a brown-black colored solution suggesting the successful fabrication of CDs. The solution was neutralized using a NaOH solution after the addition of water followed by its filtration and centrifugation for 20 min at 20,000 rpm. The smaller particles and excess amount of NaOH were removed after dialysis of the solution for 48 h. The resulting dialysate was further dried to obtain the solid CDs. The highest production efficiency of CDs was around 110 mg per Gram of algal powder used. UV-Vis spectra identified weak absorption peaks at 250 nm (aromatic C=C π-π* transition) and 330 nm (C=O bonds n-π* transition) as well as an extension peak at 580 nm. Under UV exposure (365 nm), the particles exhibited intense blue-colored luminescence. The particles showed emission intensity at 438 nm when excited with a 360 nm wavelength of light. FTIR spectral studies demonstrated absorbance peaks specific to the stretching vibrations of O-H (3,050 cm^−1^), C=O (1763 cm^−1^), C-O (1,279 cm^−1^), intramolecular hydrogen-bonded carboxylic O-H (2,670 and 2,630 cm^−1^), C=C (1,590 cm^−1^), aliphatic C-H (2,960 cm^−1^), methyl or methylene groups (1,495 cm^−1^) and a carbonization peak (570 cm^−1^). The zeta potential of CDs was −22.3 ± 8.39 mV. TEM analysis identified the quasi-spherical structure (8.5 ± 5.6 nm) as well as their uniformly separated monodispersed nature. The amorphous nature of the CDs was defined by XRD analysis. The particles were mainly composed of 59% C, 41% O and a slight amount of Na. A QY of 13% was noted compared to that of quinine sulfate (0.54). The mean lifetime of the CDs was 1.92 ns.

Al-Ghamdi et al. (2023) developed a novel approach for carbon quantum dot (CQD) fabrication using *Halimeda opuntia* and also studied their application in the storage of energy. The algal biomass was initially ground and around 0.2 g of powder was pyrolyzed at 500°C for 3 h followed by cooling down to room temperature. The color of the algal powder (green color) changed to black-grey after carbonization and was further dissolved in water via sonication (30 min). The suspension formed was subjected to 15 min of centrifugation at 15,000 rpm followed by filtration and dialysis which resulted in the formation of the final product (CQDs). The modification of the glassy carbon electrode (GCE) with CQDs was carried out by polishing the GCE (5 mm) using slurries (1 and 0.5 mm) of alumina followed by its sonication with ethanol, acetone, and water. The electrode was further dried at room temperature and was further coated directly with 5 mg/L suspensions of CQDs which resulted in the formation of CQDs-modified GCE (CQDs/GCE). UV-Vis analysis identified the absorption peaks at 350 and 245 nm that were attributed to the n-π* and C-C transitions, respectively. The yellow-colored solution of CQDs in the daylight suggested its successful synthesis. CQDs emitted intense blue color under UV exposure. At a 285 nm excitation wavelength, the particles showed an emission wavelength of 422 nm. FTIR studies revealed absorbance peaks denoting the stretching vibrations of O-H (3,432 cm^−1^), characteristic CQDs peak (3,400 cm^−1^), C-H (3,000–2,500 cm^−1^), C=O (1785 cm^−1^), -CONH- (1,484 cm^−1^), C-O-C asymmetric (1,081 cm^−1^), and C-H (711 cm^−1^). XRD analysis identified the semi-crystalline nature of the CQDs. HR-TEM analysis defined the monodispersed nature of the particles having spherical morphologies and sizes less than 5 nm. The stable nature of the particles was indicated by the negative zeta potential value (−32 mV).

Kim et al. (2022a) carried out synthesis of CNDs by the use of sea cauliflower *Leathesia difformis*. About 25 g algal biomass was washed, frozen, and ground followed by the addition of 20 mL water. The sample was further sonicated for a period of 45 min and was heated for 4 h at 180°C. The reaction mixture was further cooled to room temperature which resulted in the formation of a brown-yellow colored solution. The solution was further filtered and dialyzed followed by 40 min of centrifugation at 88,000 ×g. The resulting supernatant was stored in dark conditions at 4°C and the final concentration of the particulate solution was about 21.4 mg/mL. The solution of particles was yellow-brown colored under natural light exposure whereas, turned into light-green and coral blue under exposure of LED light (430–440 nm) as well as UV light (365 nm). UV-Vis analysis identified an absorption peak at 290 nm and 294 nm attributed to the aromatic π-π* transition of C=C and peak at 324 nm attributed to the n-π* transition of C=O bonds of CNDs. The highest emission peak of 450 nm was noted for the excitation wavelength of 370 nm. In the initial 180 min, the PL intensity of 82% was observed for CNDs. FTIR spectra revealed the peaks for the stretching vibrations of O-H (3,369.38 cm^−1^), N-H (3,000–3,700 cm^−1^) C-H (2,941.68 cm^−1^), C=O (1,637.94 cm^−1^), O-H (1,414.09 cm^−1^), C-N (1,134.72 cm^−1^), C-O (1,107.81 cm^−1^), and C=C (997.01 cm^−1^). TEM images confirmed the quasi-spherical morphology of the particles with a size ranging from 17 nm to 57 nm with a mean particle size of 10 nm. The CNDs showed the presence of elemental C (45.64%), O (32.47%), N (4.39%), Cl (5.56%), Mg (3.66%), Na (3.22%), Si (2.72%), and S (2.35%).

Placido et al. (2019) developed a new method for the synthesis of CDs by using microalgal biochar (MAB) and evaluated their use in sensing heavy metals. Initially, the biochar (5%) was depolymerized by treating with a 10% KMnO_4_ solution at 120°C for 1 h and 15 psi pressure. After centrifuging for 20 min at 5,000 rpm at room temperature, the upper phase was withdrawn and dried followed by resuspension in water and 1 min of sonication, The MAB-CDs formed were further suspended in water that exhibited an emission of 398 nm upon excitation with a 280 nm wavelength. FTIR analysis demonstrated the absorbance peaks for stretching vibrations of aromatic carbons (1,561 and 1,413 cm^−1^) and different peaks were observed at 1,667, 2,957, 2,933, 2,871, 719, and 648 cm^−1^. AFM studies identified the height of the particles ranging from 2.9 to 7.3 nm along with the mean height of 4.7 ± 0.9 nm. The size of the particles ranged from 38 to 153 nm, with the mean particle size being 68 ± 25 nm. The mean hydrodynamic diameter was 175.5 nm while the zeta potential was −39.9 mV.

Guo et al. (2017) developed CQDs by the use of microalgal powder and used it in cellular imaging and cytotoxic studies. The process of CQDs synthesis was initiated by stirring the aqueous solution of 5 g microalgal powder (50 mL water) at 25°C for 2 h. Then, 20 mL of formaldehyde solution was added and stirred for 10 min at 25°C. The reaction mixture was further heated at 180°C for around 10 h followed by cooling and centrifugation. The supernatant was purified to obtain a solution of CQDs. UV-Vis analysis identified the absorption peak at 264 nm which was mainly due to the aromatic π-π* transitions. FTIR spectral studies determined the absorbance peaks for the stretching of O-H and N-H (3,310 cm^−1^), CH_2_ asymmetric (2,930 cm^−1^) and symmetric (2,860 cm^−1^) of lipids, C=O (1,660 cm^−1^), N-H (1,540 cm^−1^), C-O-C (1,243 cm^−1^), and C-OH (1,075 cm^−1^). The reduction in the intensity of the 3,680–2,800 cm^−1^ band suggested a reduction of lipid content whereas the shifting in the 1780–1,540 cm^−1^ region (hypochromatic) suggested microalgal degradation via dehydration. The particles showed emission at 435 nm upon excitation with a 360 nm wavelength. The PL QY of the CQDs was 4.3%. TEM images exhibited spherical structures ranging from 1 to 8 nm. The particles showed high stability, a yellow-brown color and was a transparent solution without any precipitation upon suspension in water.

Zhang et al. (2017) developed a novel approach for the fabrication of N/S-CDs by the use of *Nannochloropsis* biomass and further used them for imaging of cells. The microwave assisted synthesis involved 1 g of algal biomass that was treated for 30 min. The resulting brown nitrogenous activated polymers were treated with 0.5 mL of concentrated H_2_SO_4_ under constant stirring to complete the carbonization. The reaction mixture was subjected to 30 min of stirring at 80°C followed by dilution using 20 mL water. The pH was adjusted to 7 with NaOH. The suspension formed was filtered to obtain a dark brown colored solution of N/S-CDs. The particle solution was purified via dialysis for 5 h and a final product N/S-CDs of 0.25 g was obtained. UV-Vis and PL analysis identified the transparent yellow-colored solution of particles in daylight which further emitted blue luminescence under UV exposure (365 nm). FTIR analysis revealed absorbance peaks defining the stretching vibrations of O-H and N-H (3,400 cm^−1^), C=O (1,643 cm^−1^), C-N and N-H (1,384 cm^−1^), and C-O, C-N, and C-S (1,123 cm^−1^). XRD analysis confirmed the amorphous nature of the particles. HRTEM studies proved the presence of monodispersed spherical particles with a size ranging from 2.5 to 6 nm and a mean particle size of 4 nm. The elemental composition of the fabricated particles included sulfur (3.87 wt%), carbon (53.25 wt%), nitrogen (4.41 wt%), oxygen (30.72 wt%), magnesium (3.31 wt%), and calcium (1.92 wt%).

Amjad et al. (2019) fabricated CQDs employing a hydrothermal approach with the use of a microalgae *Pectinodesmus* sp. strain PHM3 and further evaluated their anticancer application. Initially, the strain was cultured in the BBM medium and was subjected to white light illumination for 25 h at 26°C–28°C for a period of 14 days. The air pumps were used to provide constant aeration to the medium. The cells were harvested after the completion of the incubation period followed by drying. About 1 g microalgal powder was added into 50 mL water under constant stirring at 50°C–60°C for 10–15 min. This light green colored solution was further heated for 3 h at 220°C and was cooled to room temperature. The final solution (dark green) was centrifuged for 30 min at 16,000 rpm followed by filtration. The particles showed an absorption peak at 236 nm along with a small peak at 254 nm in its UV-Vis spectra that was attributed to the C=C bond π-π* transitions. A shoulder peak corresponding to C=O (n-π*) was also observed at 300 nm. The particles exhibited an emission wavelength of 472 nm at an increasing excitation wavelength from 300 nm to 400 nm. A further increase in excitation wavelength up to 480 nm resulted in an intense redshift (548 nm) in the emission wavelength. The color of CQDs turned green under UV exposure (365 nm). SEM analysis showed a spherical morphology with a 67 nm size. Some agglomerated structures were also noted. The poor crystalline nature of the particles was suggested from the XRD analysis which also further detected the presence of amorphous carbon frame in the CQDs. FTIR analysis revealed absorbance peaks at 3,286 cm^−1^ (O-H and N-H), 2,920 cm^−1^ (C-H decomposition), 2,852 cm^−1^ (COOH decomposition), 1,636 cm^−1^ (aromatic C=O), 1,440 cm^−1^ (aromatic C=C), 1,524 cm^−1^ (N-H bending), and 1,370 cm^−1^ (C-N and N-H groups).

Kim et al. (2022b) developed a novel approach for the synthesis of carbon nanodot-ZnO composites (CNDs-ZnO) from *S. horneri* and further investigated their antibacterial and antifungal activities. Initially 2 g algae was washed and dried at 25°C. The dried brown algae was further lyophilized and crushed in order to form a fine powder which was further added in the mixture of 15 mL ethanol and 15 mL water supplemented with 10 wt% zinc acetate. The mixture was sonicated and heated for 6 h at 180°C followed by cooling down to room temperature. The final solution was centrifuged for 30 min at 5,500 rpm and the supernatant was filtered which finally formed a CNDs-ZnO solution. The concentration of the NCs was 58–60 mg/mL. The particles appeared brown in color under natural daylight while they appeared a yellow-green color under LED light (430–440 nm) and a light coral blue color under the exposure of UV light (365 nm). UV-Vis spectra showed absorption peaks between 240 and 480 nm for the fabricated CNDs. The absorption peak at 265 nm was attributed to the carbonic core center (π-π* transition of C=C bonds) whereas the peak at 326 nm was attributed to the n-π* transition of C=O bonds. The particles exhibited the highest emission at 500 nm with an excitation wavelength of 420 nm. FTIR spectral studies identified absorbance peaks corresponding to the stretching vibrations of O-H/N-H (3,227.79 cm^−1^), C-H (2,977.77 cm^−1^), C=O (1,550.18 cm^−1^), C-H (1,402.96 cm^−1^), C-N (1,088.07 cm^−1^), C-O (1,046.78 cm^−1^), O-H (1,343.47 cm^−1^), and = C-H (879.86 and 946.70 cm^−1^). The atomic composition of CNDs-ZnO mainly comprised of C (54.78%), N (2.95%), O (32.40%), and Zn (9.87%). TEM images revealed the spherical structure of the particles with sizes ranging from 70 to 90 nm. EDS analysis identified the presence of C, O, and Zn along with the residues of Na, Mg, and Cl in the fabricated particles.

### 2.2 Cadmium selenide quantum dots

Zhang et al. (2018) introduced an eco-friendly approach for the fabrication of fluorescent CdSe QDs by the use of the microalgal species *C. pyrenoidosa* and *Scenedesmus obliquus* and further studied its use in determining imatinib. The cells of the microalgal species were initially maintained at 25°C in a blue-green medium (BG) under the constant illumination of light in the form of light and dark cycles (12 h each). Around 1 × 10^6^ cells/mL were initially cultured. After achieving the exponential phase of growth, harvesting of the cells was performed via 15 min of centrifugation at 4,500 rpm followed by their suspension in 20 mL water (sterile). About 200 µL Na_2_SeO_3_ (0.1 mol/L) was added to cells of algae for around 12 h followed by the addition of 200 µL Cd (NO_3_)_2_ (0.1 mol/L) and incubating the mixture for 12 days in order to fabricate CdSe QDs. The demonstration of intense yellow-orange luminescence under UV exposure also confirmed the successful synthesis of CdSe QDs. UV-Vis analysis identified absorption peaks at 300 and 372 nm attributed to protein absorption and first quantum confinement absorption, respectively, in the case of CdSe QDs derived from *C. pyrenoidosa*. Similarly, in the case of QDs derived from *S. obliquus*, the shoulder absorption peak was observed at 302 nm and the peak at 365 nm was attributed to the first quantum confinement. The emission of 470 and 480 nm was noted by QDs synthesized from *C. pyrenoidosa* and *S. obliquus*, respectively under UV exposure (365 nm). FTIR spectra determined the absorbance peaks associated with the stretching vibrations of amide I (1,600–1,690 cm^−1^), protein amide II (1,480–1,575 cm^−1^), -OH (3,304 cm^−1^), and C-H (2,920, 2,856, and 1,058 cm^−1^). TEM images showed the monodispersed nature of the QDs along with the spherical morphology with a mean particle size of 4.15 ± 0.05 nm. XRD analysis identified the face-centered cubic structure of QDs from the 2θ values of 23.44° (100) and 28.74° (101). EDX analysis confirmed elemental Cd and Se in the fabricated QDs. The optimum conditions for the CdSe QDs fabrication from the selected microalgal species mainly comprised of 0.75 mmol/L Na_2_SeO_3_ (6 h) and 1 mmol/L Cd(NO_3_)_2_ followed by 12 days of incubation.

### 2.3 Silicon quantum dots

Li et al. (2019b) used *Thalassiosira rotula* for the fabrication of silicon quantum dots (SiQDs) and checked their phytophysiologcal effect on cucumber seedlings. The diatoms were purified via 10 min of centrifugation (thrice) at 6,000 rpm. About 0.1 g trisodium citrate dihydrate was mixed with 10 mL diatom suspension followed by heating of the reaction mixture at 200°C for 5 h. After cooling to 30°C, it was filtered in order to remove impurities from the obtained SiQDs. UV-Vis analysis demonstrated the absorption peak at 410 nm for the particles. PL studies showed a dual red emission (580–640 nm and 640–720 nm) by the particles upon excitation with a 400 nm excitation wavelength. FTIR analysis identified absorbance peaks at 830, 1,080, 1,256, 1,410, 1,590, and 3,430 which was attributed to the stretching vibrations of Si-N, Si-O, Si-C, C-O, C=O, and N-H or O-H, respectively. TEM images identified the uniformly dispersed spherical-shaped particles having a mean particle size of 2 nm. The particles were clear and transparent under ambient light whereas they exhibited red luminescence under UV exposure (365 nm). The zeta potential was - 44.3 mV. XPS analysis showed the occurrence of Si, C, N, O, and Na in the fabricated particles.

## 3 Phytogenic quantum dots

Various plants have been used for making QDs as listed in Table 2 which have attractive physico-chemical and opto-electronic properties. This section discusses the synthesis process and various parameters significant for the phytofabrication of QDs.

**TABLE 2:** Phytogenic QDs and their applications.

Name of plant	Type of quantum dots	Size (nm)	Shape	Activity/application	Reference
*Aloe barbadensis*	CQDs	5	spherical	-	Malavika et al. (2021)
Aloe vera	CeO_2_QDs	2.6–3.5	spherical	antimicrobial	Pisal et al. (2019)
*Andrographis paniculata*	CDs	8–11	-	anticancer and antioxidant	Naik et al. (2020)
Bamboo timber waste	GQDs	2–8	spherical	curcumin detection	Tade and Patil (2020)
Betel leaf	CQDs	3–7	-	Fe^3+^ sensing	Raja and Sundaramurthy (2021)
Broccoli	CQDs	2–6	spherical	Ag^+^ detection	Arumugam and Kim (2018)
*Calotropis gigantea*	CQDs	5.7	spherical	bioimaging	Sharma et al. (2022)
*Camellia sinensis*	CdSQDs	3–5	spherical	antimicrobial and hemolysis	Shivaji et al. (2018)
*Catharanthus roseus*	CQDs	5	spherical	antioxidant, anticancer, and multi-ion detection	Arumugham et al. (2020)
*Clitoria ternatea*	GQDs	10–20	spherical	-	Tak et al. (2020)
*Echinops persicus*	CQDs	4–6	regular shrub	-	Nasseri et al. (2020)
*Eclipta alba*	ZnOQDs	3–9	spherical	antimicrobial	Singh et al. (2018)
*Eichhornia crassipes*	AgQDs	less than 10	icosahedral, cubic, and rhombohedric	antimicrobial	Silva et al. (2017)
*Elettaria cardamomum*	CDs	-	-	photocatalytic degradation of CR and MB dye	Zaib et al. (2021)
*Ficus johannis*	CdTeQDs	3.7	spherical	antimicrobial	Alvand et al. (2019)
*Lawsonia inermis*	CDs	5	quasi-spherical	anticancer drug sensing and antimicrobial	Shahshahanipour et al. (2019)
Lemon juice	CQDs	12–15	-	-	Hoan et al. (2019)
*Mangifera indica*	GQDs	2–8	-	cell cycle analysis	Kumawat et al. (2017)
*Manikara zapota*	CDs	1.9 ± 0.3 (blue-CDs), 2.9 ± 0.7 (green-CDs), and 4.5 ± 1.25 (yellow-CDs)	spherical	bioimaging	Bhamore et al. (2019)
*Musa acuminata*, *Citrus limetta*, *Citrus indica*, and *Annona squamosa*	CDs	5–37	-	bioimaging	Pete et al. (2023)
Neem	GQDs	5.0 ± 0.4	spherical	cell imaging	Roy et al. (2015)
Nutmeg seeds	GQDs	6.1	face-centered cubic	antioxidant, antimicrobial, antifungal, and anticancer	Anooj and Praseetha (2019)
*Opuntia* sp.	GQDs	2.6 ± 0.63	quasi-spherical	phytic acid and phosphates detection	Centeno et al. (2021)
*Phoenix dactylifera*	CQDs	20–630	quasi-spherical	-	Tungare et al. (2020)
*Plectranthus amboinicus*	CQDs	less than 5	-	-	Sunitha et al. (2021)
Polar wood	CQDs	3.4–4.6	-	-	Gong et al. (2019)
Potato	CdSQDs	5	-	Ag^+^ detection	Wang et al. (2021)
Pseudo-stem of banana plant	CDs	2–3	spherical	nanosensor, bioimaging, and cytotoxic effect	Vandarkuzhali et al. (2017)
*Psidium guajava*	GQDs	-	-	Hg^2+^ detection	Khose et al. (2021)
*Rhaphanus sativus* L	CdSQDs	4–6	-	anticancer	Gholami et al. (2020)
Sandalwood	CQDs	3.5	quasi-spherical	-	Shukla et al. (2020)
Sugarcane bagasse pulp	CQDs	4.1 ± 0.17	spherical	-	Thambiraj and Shankaran (2016)
Sweet potato	CDs	3.39	spherical	Fe^3+^ sensing, bioimaging	Shen et al. (2017)
*Trapa bispinosa*	CDs	5–20	spherical	cytotoxic effect	Mewada et al. (2013)
*Azadirachta indica*, *Ocimum tenuiflorum*, and *Tridax procumbens*	CQDs	10	-	biocompatibility and bioimaging	Meena et al. (2019)

### 3.1 Cadmium sulphide quantum dots

**FIGURE 2:**
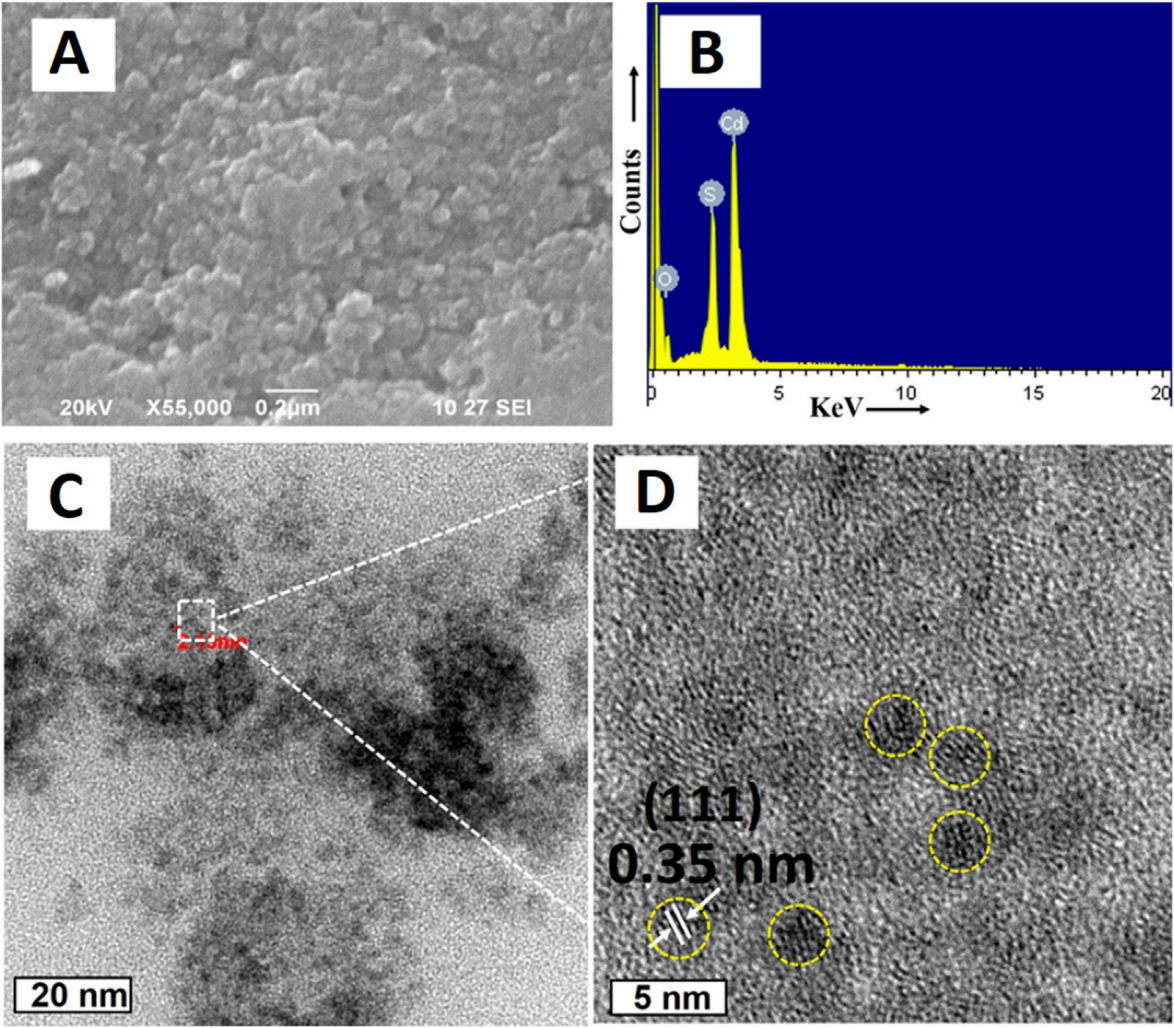
**(A)** SEM image of CdS QDs; **(B)** EDAX spectrum of CdS QDs; HRTEM image of the synthesized CdS QDs **(C)** at a 20 nm scale and **(D)** high magnification at a 5 nm scale. Reprinted with permission from Shivaji et al. (2018). Green-synthesis-derived CdS quantum dots using tea leaf extract: Antimicrobial, bioimaging, and therapeutic applications in lung cancer cells. *ACS Appl. Nano Mater.* 1 (4), 1,683–1,693. doi:10.1021/acsanm.8b00147. Copyright^©^ 2018 American Chemical Society.

Shivaji et al. (2018) synthesized CdS QDs from the *C. sinensis* leaf extract (tea leaf extract). The fabrication was carried out in two different stages, in the first stage, 30 mL tea leaf extract and 2 mL CdSO_4_ (0.025 M) were mixed and incubated for 3 days in dark conditions. In the second stage, the previous mixture was further mixed with 0.5 mL Na_2_S (0.025 M) and the solution was again incubated in the same conditions for 4 days in order to obtain CdS QDs. A bright yellow colored solution was formed which was further centrifuged (13,000 rpm, 10 min). The contaminants were removed by repeated washings (3 times) of CdS QDs and the pellet obtained was further lyophilized. The deviations in the functional groups revealed by FTIR after comparing peaks of the tea leaf extract and CdS QDs confirmed CdS formation from the weak peak obtained at 630 cm^−1^. Along with it, other peaks at 1,633, 3,421, and 1,376 cm^−1^ were mainly attributed to the C=O (amide group), O-H (carboxyl group), and N-H bending (amide-II), respectively. One of these constituents was involved in the capping of CdS QDs that had a smooth and spherical morphology as indicated from the SEM images as evident from Figure 2. The EDS spectra displayed intense peaks of Cd and S (confirming the CdS formation) and oxygen (originated from the organic capping material). The size of the particle ranged from 3 to 5 nm and the distribution was homogeneous, with the average size being 3 ± 1 nm.

**FIGURE 3:**
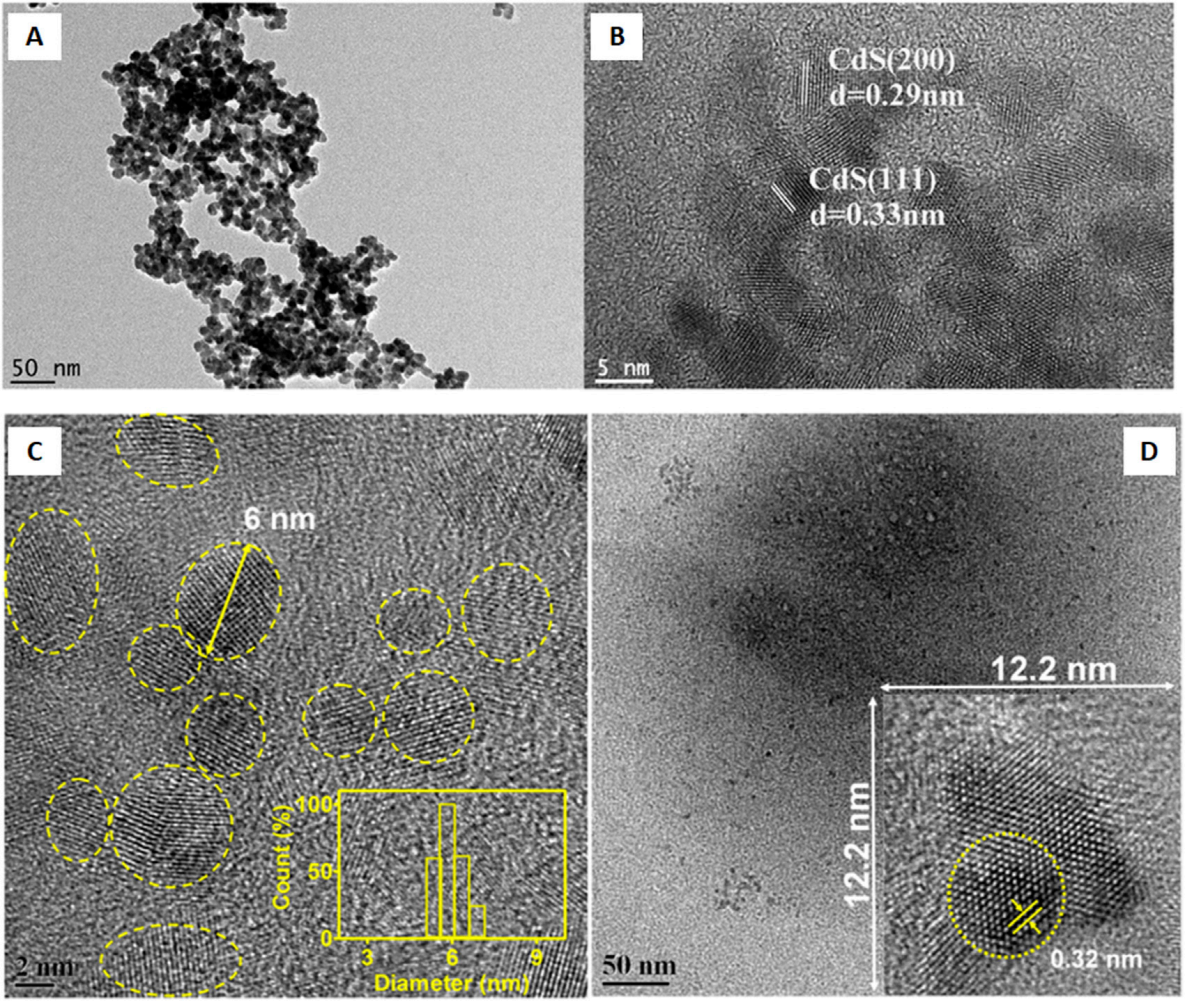
**(A)** TEM and **(B)** high-resolution TEM (HRTEM) images of prepared water-soluble CdS quantum dots. Reprinted from Wang et al. (2021). One-step synthesis of water-soluble CdS quantum dots for silver-ion detection. *ACS Omega* 6 (10), 7,139–7,146. doi:10.1021/acsomega.1c00162. Copyright^©^ 2021 The Authors. Published by American Chemical Society (Open access); **(C)** FEG-TEM micrograph of mGQDs at a 2 nm scale (inset showing size distribution characterization using DLS); **(D)** Micrograph showing mGQDs at a 50 nm scale with an inset showing a frame with dimensions of 12.2 × 12.2 nm displaying a high-resolution image of mGQDs with a fringe lattice width of 0.32 nm. Reprinted with permission from Kumawat et al. (2017). Graphene quantum dots from *Mangifera indica*: Application in near-infrared bioimaging and intracellular nanothermometry. *ACS Sustain. Chem. Eng.* 5 (2), 1,382–1,391. doi:10.1021/acssuschemeng.6b01893. Copyright^©^ 2016 American Chemical Society.

Wang et al. (2021) investigated CdS QDs fabrication from potato extracts. Thioacetamide (TTA, 2.5 g) and 3CdSO_4_.8H_2_O (0.275 g) were mixed into the potato extract and further the reaction mixture was heated at 121°C for 20 min. After the completion of the reaction, the solution was further cooled to room temperature, and thus finally a uniformly dispersed water-soluble CdS QDs was obtained. The UV-Vis spectrum revealed the highest absorption at 330 nm. The fluorescence spectrum identified an excitation wavelength at 330 nm while the emission wavelength was at 490 nm. The FTIR spectrum showed enhanced peaks at 2,922, 1,630, 1,538, 1,012, and 702 cm^−1^ that were attributed to C-H vibrations, carbonyl stretching, amide stretching vibrations, C-OH bond stretching vibrations, and C-S bond stretching, respectively. The uniformity in size, better dispersity, crystalline nature, and size (5 nm) of the particle was identified by using TEM analysis as evident from Figures 3A, B. XPS analysis showed elemental Cd, C, O, and S on the CdS surface. On the basis of FTIR and XPS analysis, the components stabilizing the CdS QDs were speculated to be proteins, minerals, starch, amino acids, and other substances present in the potato extract.

Gholami et al. (2020) reported the formation of CdS QDs by hairy roots of *Rhaphanus sativus* L. About 30 mL of the root extract was stirred at 28°C with 250 rpm. Further, 2 mL of CdSO_4_ (0.025 M) was added to it followed by continuous stirring for 3 days in the dark with 250 rpm at 28°C. 500 μL of Na_2_S (0.5 M) was further added to the yellow colored solution and the reaction mixture was stirred at 28°C and 250 rpm under dark conditions. The solution was further centrifuged at 5,000 rpm for 10 min after 4 days and the supernatant obtained was used in the experiment while the salt-free extract was taken as the control. UV-Vis spectra of the CdSQDs revealed the absorption between 350 and 550 nm with the highest absorbance maxima 460 nm. The size of the particle was 4–6 nm. The highest emission of 520 nm was emitted upon excitation of CdS QDs with 370 nm of an excitation wavelength. The functional groups present on the surface of CdS QDs exhibited absorption peaks at 1,634.32, 2069.29, and 3,436.05 cm^−1^ that were specific to the amide compounds (N-H), C-H, and OH groups.

### 3.2 Cadmium telluride quantum dots

Alvand et al. (2019) employed a rapid green synthesis method for cadmium telluride quantum dot (CdTe QD) fabrication from the *Ficus johannis* plant. The green synthesis began by adding 50 mL of Cd(NO_3_)_2_.7H_2_O to 25 mL of the plant extract (pH 9). Around 50 mL Te solution (5 mM) along with NaBH_4_ (5 mM) was added to the reaction mixture. After the completion of the reaction, the collected CdTe QDs solution was further placed at 5°C. The absorption band ranging from 400 to 425 nm was identified for all of the samples. Fluorescence spectra revealed an intense symmetric emission peak (440–480 nm). TEM analysis showed a spherical shape of the particles with an average size of 3.7 nm. The FTIR studies were carried to identify the nature of the capping layer from the peaks obtained at 1,500–1,637 cm^−1^ (C=O), 3,438 cm^−1^(-OH), 3,079 cm^−1^(=C-H), and 1,400 cm^−1^ (C=C aromatic stretching), respectively. The mean particle size calculated from the XRD spectrum was 3.27 nm and the EDX results confirmed elemental Cd and Te in the CdTe QDs.

### 3.3 Cerium oxide quantum dots

Pisal et al. (2019) evaluated cerium oxide quantum dot (CeO_2_ QD) synthesis from the *A. vera* leaf extract. The synthesis of QDs began by boiling 100 g *A. vera* leaves (fresh) in 100 mL distilled water for 10 min. The mixture of 2.7 g of cerium (III) nitrate hexahydrate (Ce(NO_3_)_3_.6H_2_O) in distilled water (35 mL) was further added to the aqueous leaf extract (65 mL) followed by 30 min of stirring. Yellow-white colored precipitates were formed after sonicating the solution for 2 h at 80°C. The precipitates were further dried at 90°C for 4 h and crushed to obtain a fine pale yellow colored powder of CeO_2_ QDs followed by 2 h stirring at 400°C. UV-Vis spectroscopy revealed the highest absorption peaks for CeO_2_ QDs at 333 nm. The strongest intensity at 458 cm^−1^ was revealed by Raman spectroscopy which corresponds to the symmetric Ce-O stretching mode of vibrational units. The identification of surface functional groups through FTIR spectroscopy revealed different peaks that were attributed to C=O (2,348 cm^−1^), O-H stretching vibrations (3,541 cm^−1^), carbonate species (1,358 cm^−1^), C-O stretching vibrations (1,036 cm^−1^), and Ce-O stretching (below 500 cm^−1^). The PL spectroscopy detected 325 nm as the excitation wavelength and different emission peaks at 452, 469, 482, and 493 cm^−1^ exhibited by the synthesized CeO_2_ QDs. The blue shift confirmed the successful synthesis of QDs. The single cubic fluorite phase of the particles was identified from XRD analysis. The aggregated porous spherical microstructured QDs were observed in the SEM images. The TEM analysis confirmed the formation of QDs by identifying small crystalline agglomerates with a size of 2–3 nm.

### 3.4 Silver quantum dots

Silva et al. (2017) synthesized silver quantum dots (AgQDs) from *Eichhornia crassipes* (EC) by taking 2 m^2^ of the biomass that was repeatedly washed and then dried at room temperature for 15 days. Leaves, stem, and roots were separated and ground individually and then further washed and dried (50°C) for a period of 48 h. The biomass obtained was reacted with a 0.003 M AgNO_3_ solution that resulted in the appearance of brown color that indicated the successful fabrication of AgQDs. The absorption maxima of roots, stems, and leaves of EC were in between 277 and 280 nm which was attributed to the existence of polyphenols in plants. EDS analysis confirmed the existence of Ag along with C, O, and P in the AgQDs fabricated from leaves. The particles were less than 10 nm in size. The mean particle size of AgQDs fabricated at pH 12, 10, 7, and 4 were 4.18 ± 3.9, 5.7 ± 2.6, 6.9 ± 7.7, and 7.8 ± 9.6 nm, respectively. The AgQDs synthesized from the leaves of EC showed the highest stability. The AgQDs synthesized at different pH values were of various shapes and structures like icosahedral, cubic, and rhombohedric.

### 3.5 Zinc oxide quantum dots

Singh et al. (2018) developed a novel sustainable method for synthesizing zinc oxide quantum dots (ZnO QDs) employing *Eclipta alba* leaf extracts. ZnO QDs were fabricated by mixing 5 mL leaf extract with an equal volume of a zinc acetate solution (1 mM). The change in color from dark brown to yellow after continuous stirring at 20°C for 5 min confirmed the successful fabrication of ZnO QDs. The mechanism behind ZnO QDs synthesis involved the reduction of Zn^2+^ to Zn^0^ atoms (activation stage) due to the bioreducing agents present in the leaf extract. Conversion of Zn^0^ to ZnO (nucleation stage) resulted due to the reaction with dissolved oxygen from the reaction mixture, followed by the spontaneous aggregation of small QDs to form larger particles (growth stage). The spherical shape evolution was governed by the phytochemicals that acted as stabilizing/capping agents (termination/stabilization stage). UV-vis absorption revealed the highest peak at 324 nm. The solution containing 5 mM reaction mixture and 5 mL leaf extract demonstrated the maximum synthesis of ZnO QDs. The optimum amount of leaf extract was 7 mL for 5 mL of zinc acetate while the optimum temperature was 40°C. The neutral pH of the medium was optimum for the biosynthesis of ZnO QDs. The effective reaction time for optimum yield of Zn QDs was 75 min. XRD analysis confirmed the hexagonal wurtzite phase of ZnO QDs and the average crystalline size of the particles was around 6 nm. XPS analysis of the particles defined their surface composition and chemical states. The monodispersed spherical structure with 6 nm size was revealed from TEM analysis. It also showed a homogeneous narrow size distribution pattern ranging from 3 to 9 nm.

### 3.6 Carbon quantum dots

Malavika et al. (2021) investigated the green synthesis of CQDs by using *Aloe barbadensis* miller extracts. The microwave-assisted reflux was employed for the synthesis of CQDs. A mixture of smashed fresh leaves (50 mg) and double-distilled water (50 mL) was microwaved, centrifuged (5,000 rpm for 20 min), purified by silica gel column separation and dialyzed. The formation of CQDs resulted due to the continuous heating of fluorophores that led to their polymerization, condensation, and carbonization. The optical studies of CQDs via UV-vis spectra identified the highest absorption peak at 301 nm (n-π* electronic transition of a C=O bond) and two small peaks at 257 and 274 nm (π-π* electronic transition of a conjugated C=C segment), respectively. CQDs synthesized at 8 min of reaction time had the maximum absorbance and photo-luminescence (PL) emission spectrum (at 360 nm excitation wavelength). X-ray diffraction (XRD) analysis of CQDs confirmed their amorphous nature. The study of functional groups of the CQDs by FTIR confirmed the presence of carboxylic acid (O-H and C=O groups) from the broad peaks obtained at 3,337 cm^−1^ and 1,650 cm^−1^ and also denoted the aromatic C-H bending by the peak at 662 cm^−1^. PL analysis of CQDs detected the highest intensity of emission peak at 434 nm (blue emission) for 360 nm of excitation wavelength which originated due to the combination of excited electrons of C=O groups during n-π* transition. The -OH groups present on CQDs mainly resulted in blue emission and aided in uniform dispersity in an aqueous solution. An increase in excitation wavelength from 360 to 420 nm resulted in a decrease in emission intensity. X-ray photoelectron spectroscopy (XPS) analysis revealed 56.33% C and 43.67% O as the main elements in the CQDs. Quantum yield (QY) evaluated by quinine sulfate as the standard was 31%. HRTEM revealed the spherical, monodispersed, and non-aggregated particles with a size below 5 nm.

Raja and Sundaramurthy (2021) studied the fabrication of fluorescent CQDs by using betel leafs (*Piper betle*). The leaves were initially washed and crushed to prepare leaf juice which was further heated at 180°C for 24 h, cooled at ambient temperature, and filtered. After filtration, the CQDs were centrifuged at 10,000 rpm in order to remove large sized carbon materials and purify the CQDs. Two different absorption peaks at 215 nm and 275 nm attributed to π-π* (C=C) and n-π* (C=O) transitions were identified by UV-vis spectroscopy. TEM images revealed an abundance of monodispersed nanoparticles along with their amorphous nature as well as sizes ranging from 3 to 7 nm. From th PL spectra, the strongest emission at 456 nm was obtained for 380 nm of excitation wavelength for the synthesized CQDs.

Arumugam and Kim (2018) used broccoli to synthesize CQDs by a one-step hydrothermal process. Initially, the mixture of 50 mL broccoli juice and 25 mL distilled water was heated at 190°C for 6 h in order to carry a hydrothermal reaction. The resulting dark brown colored solution of CQDs was filtered and further purified by centrifugation at 8,000 rpm for 20 min to derive the pure dark brown colored CQDs from the supernatant. The optical characterization of CQDs as analyzed using UV-vis spectroscopy revealed π-π* transitions of the C=C bond as strong peaks at 282 nm and n-π* transitions of the C=O bonds as shoulder peaks at 233 nm. An intense blue PL was emitted from the CQDs upon exposing UV light. The excitation and emission peaks for the CQDs were observed at 355 nm and 450 nm, respectively. With an increase in pH from 2 to 7, there was an enhancement in PL while a gradual reduction in PL was observed at pH 7 to 12, respectively. The amine groups on the surface of the CQDs were confirmed by the FTIR spectrum which also detected a O-H stretching vibration (3,617 cm^−1^) and N-H stretching vibration (3,457 cm^−1^). Further, the C=N, C=O, C=C, and C-H stretching vibration associated peaks were obtained at 1,301 cm^−1^, 1700 cm^−1^, 1,519 cm^−1^, and 3,079 cm^−1^, respectively. The N-H bending vibration was noted at 850 cm^−1^. XRD analysis indicated a poor crystalline nature having a broad peak centered at ∼19.5°. About 0.42 nm of the interlayer spacing was detected for CQDs that were mono-dispersed, spherical, and well-separated with sizes between 2 and 6 nm. The partial crystalline structure of CQDs along with lattice spacing (0.22 nm) was attributed to the graphite plane as recognized by HRTEM.

Sharma et al. (2022) reported a one-pot microwave assisted synthesis of fluorescent CQDs using the *Calotropis gigantea* (crown flower) leaf extract. The synthesis was initiated by mixing 10 g fresh crushed leaves in distilled water (100 mL) followed by microwave irradiation till a brown viscus fluid was formed. The agglomerates were separated by centrifuging the mixture at 15,000 rpm for 15 min. The supernatant was filtered to recover the CQDs suspended in water. The UV-vis spectrum exhibited the highest absorption (280 nm) along with extension of a tail to the visible range which were due to the n-π* transition (of the C=O bond) and π-π* transition (of the conjugated C=C bond), respectively. The CQDs were spherical, non-uniformly dispersed, and agglomerated with the size ranging from 2.7 to 10.4 nm, the average being 5.7 nm. The zeta potential was found around −13.8 mV which confirmed its negatively charged surface. The poly-crystalline nature and amorphous carbonaceous core-shell materials of the synthesized CQDs were identified from the XRD spectrum. The existence of different oxygen groups and their linkages in CQDs such as -OH, C=O, C-O, -C-H, and O-H were revealed by the peaks obtained at 3,487, 1745, 1,078, 2,980, and 1,380 cm^−1^ in the FTIR spectra. The sp^3^-and sp^2^-hybridization of the carbon atoms present in the CQDs were determined by nuclear magnetic resonance (NMR) spectroscopy (^1^H and ^13^C).

Arumugham et al. (2020) reported a one-pot synthesis method for CQDs using *Catharanthus roseus* (white) leaves via a hydrothermal carbonization technique. 0.5 g of finely chopped leaves in 50 mL deionized water were heated at 200°C for 4 h and allowed to cool down to room temperature. The resulting pale-yellow colored solution was filtered and purified to obtain fluorescent CQDs at pH 6. This was attributed to the -OH and -COOH groups on its surface. The optical characteristics of CQDs using UV-vis spectroscopy detected broad absorption peaks at 270 and 350 nm corresponding to n-π* and π-π* transitions due to C=O and C=C functional groups, respectively. At a 365 nm wavelength of UV light, a fluorescence of bright greenish blue color was emitted from the CQDs. TEM images identified the spherical shape of CQDs 5 nm of size that were well dispersed in the solution. Different elements like oxygen (wt. 0.73%), carbon (wt. 98.14%), and nitrogen (wt. 1.13%) were present in the CQDs. The analysis of the surface functional groups using FTIR identified O-H_(as)_ stretching (3,334 cm^−1^), aliphatic C-H_(as)_ and C-H_(s)_ (2,918–2,853 cm^−1^), C=O_(s)_ (1,693 and 1,365 cm^−1^), aromatic C=C_(s)_ (1,595 cm^−1^), C-N_(s)_ (1,442 cm^−1^), C-O_(s)_ and C-O_(b)_ (1,245, 1,103, and 1,020 cm^−1^). Upon evaluating the fluorescence properties of CQDs, the highest emission of 405 nm was observed upon excitation with 330 nm light while the highest QY was 28.2%.

Naik et al. (2020) studied carbon dot (CD) fabrication from the ayurvedic medicinal plant *Andrographis paniculata* (AP) using a hydrothermal method. The aqueous extract of fresh leaves of AP (AEAP) was prepared by adding fresh leaves (10 g) in water (150 mL) followed by boiling the mixture until the volume reduced to 100 mL. The extract was further filtered and heated at 160°C for 8 h. The CDs were centrifuged at 11,000 rpm for 15 min and purified using a 0.22 µm syringe filter. UV-vis analysis identified an absorption peak at 265 nm which was attributed to the π-π* transition of an aromatic ring present in the AAPCDs. Fluorescence in the 380–600 nm range with a maximum of 430 nm was obtained for AAPCDs when excited with a 350 nm wavelength. The FTIR analysis identified the peaks for OH and NH at 3,387 cm^−1^, C-O bond (stretching and bending) in the carboxyl group at 1,076 and 1,026 cm^−1^, C-H and CN vibrations at 2,929 and 1,408 cm^−1^, C=C stretching at 1,597 cm^−1^, and the formation of more OH groups from AEAP to AAPCD by broadening of 3,387 cm^−1^. TEM analysis revealed the size of the CDs prepared from leaves (fresh) of the AP aqueous extract (AAPCDs) which ranged from 8 to 11 nm, the average size being 9 nm. AAPCDs possessed a zeta potential value of −3.70 mV. The EDS spectrum detected elemental O, N, and C in the CDs. The fabricated AAPCDs had a pH value of 6.04 while the QY of AAPCDs was 15.10% as evaluated at 350 nm (excitation wavelength).

Nasseri et al. (2020) investigated CQDs synthesis from the *Echinops persicus* plant extract. Different organs of the plant were utilized in the synthesis process via the hydrothermal method. The reaction mixture containing 6 g of plant powder in 75 mL distilled water was sonicated at room temperature for 30 min. Further, the reaction mixture was heated for 10 h at 200°C. The maximum synthesis of CQDs was obtained at a 200°C calcination temperature and a 6 g/75 mL extract concentration. UV-vis spectroscopy revealed a wide absorption spectrum ranging from 400 to 450 nm for CQDs which suggested the transition of electrons. The size of the synthesized CQDs was between 3.5 and 5.5 nm on the basis of the green color formation. FTIR analysis of CQDs identified the characteristic C-H stretching bond from the peak obtained at 2,930 cm^−1^. XRD patterns of CQDs revealed the characteristic peak (amorphous) at 2θ = 23°. Elemental analysis of CQDs using EDX revealed the existence of 40.47 wt% (C), 40.51 wt% (O), and 3-4 wt% (Mg, K, and Cl). The surface analysis of CQDs using TEM identified a homogeneous distribution and regular shrub structure of the particles with a size between 4 and 6 nm. The homogeneous spherical shapes of CQDs were also detected by HRTEM having a size of about 5–6 nm and 0.222 nm lattice fringe spacing.

Zaib et al. (2021) synthesized CDs from *Elettaria cardamomum* (E.C) leaves by a simple ultrasonication method. The mixture of dried leaves (3 g) in 20 mL distilled water was ultrasonicated for 45 min to obtain a greenish-brown product which was further centrifuged (4,500 rpm, 15 min) to separate the supernatant. After centrifugation, the large sized particles were eliminated from the supernatant via filtration (0.22 µm). XRD analysis revealed a poor crystalline structure with an amorphous nature. UV-vis spectroscopy indicated two different absorption peaks at 220 nm and 272 nm which were denoted as -C=O and C=C bonds (π-π* transition n-π* transition), respectively. FTIR analysis identified the absorption peaks for OH and NH stretching (3,257 cm^−1^), C-H (2,919 cm^−1^), COO- (1,580 cm^−1^ and 1,400 cm^−1^), C=O (1715 cm^−1^), and C=C (1,634 cm^−1^). The peaks at 1,299 cm^−1^, 1,105 cm^−1^, and 1,027 cm^−1^ were attributed to the C-OH stretching vibration. The intense peak at 959 cm^−1^ was associated to epoxy resins. The PL spectrum bands were observed at 514 nm. The synthesized CDs showed two string emission bands at 520 nm and 850 nm, which confirmed the fluorescent nature of the synthesized CDs.

Shahshahanipour et al. (2019) discovered the fabrication of novel CDs from an ancient plant *Lawsonia inermis* (Henna). A 0.5 g leaf powder was dissolved in 40 mL of distilled water which upon stirring resulted in the formation of a greenish orange color. The solution was further heated at 180°C for 12 h and cooled until it reached room temperature. In order to remove large sized particles, the sample was centrifuged at 12,000 rpm for 20 min and filtered. The purified sample was dried (overnight) resulting in brown colored CDs. The parameters, like 180°C and 12 h reaction time, were considered optimal for the fabrication of CDs. The absorption spectrum had a broad peak from 270 to 380 nm corresponding to the conjugation of C=C and C=O bonds through π-π* and n-π* transitions, respectively. The fluorescence spectral studies identified the highest emission intensity of 440 nm for 360 nm of excitation wavelength. TEM analysis showed the quasi-spherical (5 nm) and well-dispersed nature of CDs while DLS indicated a hydrodynamic size ranging from 3 to 7 nm. The zeta potential was −39 mV which was mainly due to the carboxyl, hydroxyl, and carbonyl groups on the surface. The XRD pattern of the CDs confirmed the poor crystalline structure of the particles. The elemental configuration of CDs from the EDX analysis confirmed the abundance of C over O content, thus, proved the presence of amorphous nanocrystalline carbons. Different functional groups present on the CDs surface were identified from FTIR analysis, the peaks obtained at 3,380, 2,934, 1,585, and 1,415 cm^−1^ were attributed to the absorption bands of -OH and -NH stretching, C-H vibrations, C=C and C=O stretching, and COO^−^ stretching vibrations which played an important role in providing hydrophilicity and stability to the CDs. The QY of CDs determined by Rhodaminie B (standard) was 28.7%.

Hoan et al. (2019) investigated the synthesis of highly luminescent CQDs using lemon juice by a hydrothermal process. The CQDs were fabricated by autoclaving lemon juice (40 mL) at 120°C–280°C for the period of 12 h. The successful formation of CQDs was confirmed from the change in color (ivory white to dark brown) of the solution. The large sized particles formed along the CQDs were removed via filtration (2 µm). The CQD solution showed a green color emission under UV excitation. The UV-vis analysis identified the absorption peak at 283 nm which was mainly attributed to the π-π transition of the C=C bond. PLE spectral studies of the CQDs identified the peaks occurring in the range of 410–440 nm for particles synthesized at 240°C–280°C and around 450–480 nm for particles synthesized at 150°C–200°C. CQDs possessed the highest excitation wavelengths lying in the range of 400–480 nm. The CQDs synthesized at 150, 200, 240, and 280°C showed emission peaks at 550, 540, 518, and 508 nm, respectively. The infrared spectrum identified peaks for C=O (1713 cm^−1^), C-H bond (1,390 and 2,925 cm^−1^), COOH group vibration (1,360 cm^−1^), and C-O-C stretching (1,124 cm^−1^). The particles fabricated at 240°C for 12 h showed a hydrodynamic size of 50 nm while the zeta potential was 9.48 mV. The HRTEM studies revealed the size of CQDs synthesized at 200°C and 240°C to be around 12–15 and 3–5 nm, respectively. The amorphous nature of CQDs was also determined by the HRTEM. QY of CQDs was dependent on the temperatures at which the synthesis was carried on and were evaluated as 14.86% (150°C), 16.87% (200°C), 21.37% (240°C), and 24.89% (280°C).

Bhamore et al. (2019) derived multicolored emissive CDs from *Manikara zapota* fruits. The green CDs were fabricated by the mixture of 0.5 g freeze-dried fruit and 10 mL H_3_PO_4_ (40 N) along with water (5 mL) followed by heating of the reaction mixture (30 min, 80°C). The green CDs were prepared by dialyzing the product for 24 h with water. The yellow CDs were synthesized in the similar way where the reaction was carried out for 15 min at 80°C. The resulting CDs were isolated via dialysis for 24 h against water. The blue-colored CDs were prepared at 100°C by a similar procedure except for the treatment of fruit with H_2_SO_4_. The blue-, green-, and yellow- CDs revealed absorption peaks at 248, 395, and 489 nm which also exhibited bright blue, green, and yellow fluorescence at 365 nm under UV irradiation. These absorption transitions were mainly attributed to the π-π* and n-π* transitions. The formed CDs had fluorescence emissions at 443, 515, and 563 nm upon excitation with 350, 420, and 440 nm wavelengths, respectively. The QY of the synthesized CDs was obtained around 5.7% (blue-CDs), 7.9% (7.9%), and 5.2% (yellow-CDs). The CDs were stable up to 100 days from the time of synthesis. The average fluorescence lifetime of 4.9, 5.2, and 4.3 ns were noted for the blue-, green-, and yellow-CDs, respectively. The FTIR spectra of all of the three CDs revealed stretching vibration peaks of the -OH group (3,453, 3,449, and 3,451 cm^−1^), -C=O and amide bonds (1,635, 1,642, and 1,638 cm^−1^), -C-H (2,826, 2,853, and 2,988 cm^−1^), -C-NH-C (1,401, 1,400, and 1,401 cm^−1^), C-N/C-S (1,195, 1,143, and 1,142 cm^−1^), P-O (529, 937, and 1,082 cm^−1^), and P=O (530, 938, and 1,081 cm^−1^). The HR-TEM images revealed a spherical morphology and crystalline nature of all the CDs with the average particle size of 2.9 ± 0.7 nm (green-CDs), 1.9 ± 0.3 nm (blue-CDs), and 4.5 ± 1.25 nm (yellow-CDs). DLS analysis determined the mean hydrodynamic diameters for blue-CDs (7 ± 1.5 nm), green-CDs (8 ± 1.7 nm), and yellow-CDs (10 ± 2.1 nm).

Pete et al. (2023) fabricated fluorescent CDs from the peels of various fruits like *Musa acuminata*, *Citrus limetta*, *Citrus indica*, and *Annona squamosa*. The Microwave-assisted hydrothermal method was employed in the fabrication process. Initially, the powder of the fruit peel was dissolved separately in the distilled water (100 mg/25 mL) which was followed by baking at various temperatures for different time intervals in a microwave oven. The resulting solutions were further filtered (0.2 µ) and centrifuged for 20 min at 20,000 rpm to eliminate any impurities. The CDs synthesized from the digested *A. squamosa* peel at 160°C after 150 min showed higher fluorescence than that of other samples. The UV-vis spectrum revealed different absorbance maxima at 276, 277, 496, and 490 nm for the different *A. squamosa* peel powder concentrations like 0.1, 0.2, 0.4, 0.6, 0.8, and 1.0 per 25 mL distilled water. The spectrum showed deflection because of the π-π* transitions. The PL spectrum of the synthesized CDs was strongest at 467 nm. CDs emitted fluorescence between 420 and 500 nm wavelengths upon excitation (between 270 and 370 nm). The FTIR analysis revealed the characteristic absorption bands for O-H and N-H stretching (3,787 cm^−1^), C-H and S-H stretching (2,958 cm^−1^), C=C of polycyclic aromatic hydrocarbons (2,385 cm^−1^), N-H bending (1,643 cm^−1^), and the C-O-C and C-H bending vibrations of a pyranose ring in the peels (1,093 cm^−1^). The peak at 1,090 cm^−1^ was attributed to C-O-C and C-H groups that served as capping and stabilizing agents. The average size of the CDs was 15.4 nm at a concentration of 6.39 × 10^8^ particles/mL. The average size of particles synthesized from *A. squamosa* was in the range of 5–37 nm with a zeta potential of −20.8 mV. EDX analysis showed 99.54% C with minor impurities of Cd and S in the phytogenic CDs.

Tungare et al. (2020) reported the synthesis of CQDs using *P. dactylifera* (Date palm fruit). A bottom-up approach was employed where a mixture of 5 g of crushed fruit in 50 mL distilled water was pyrolyzed at 100°C for 6 min under microwave. The extract was further purified using the dialysis method (pore size 2.4 nm). A narrow peak of absorbance at 270 nm was identified for CQDs in the UV-vis spectra which was attributed to the n-π* transition of -C=O and π-π * transition of C=C bonds. The excitation wavelength of 250 nm and highest fluorescence intensity at 320 nm was recorded for the synthesized CQDs. The PL intensity was observed at 445 nm (highest) upon excitation with 375 nm. The FTIR spectra identified the functional groups present on CQDs as sharp peaks at 3,431 cm^−1^ (O-H stretching vibrations), 2,925 cm^−1^ (representing C-H stretching vibrations of alkanes), 2,853 cm^−1^ (O-H stretching vibrations of the carboxyl groups), left side shifting of 1,626 cm^−1^ (C=C stretching of the aromatic group and N-H bending vibrations of the amide group), 1,417 cm^−1^ (bending vibrations of C-H alkanes and C=C stretching aromatic group), and 1,023 cm^−1^ (C-O stretching of esters or ethers). The morphological data of CQDs using TEM revealed the quasi-spherical structure, spatial distribution, and size of particles between 0.02 and 0.63 µm.

Sunitha et al. (2021) derived CQDs from the *Plectranthus amboinicus* leaf extract using hydrothermal treatment for 84 min. The color of the extract converted from green to brown eventually forming dark brownish CQDs that were recovered in the supernatant after centrifugation at 5,000 rpm for 15 min. Further, the larger particles were separated from the dispersed solution by repeated centrifugation and were filtered before characterization. The highest emission of the particles was obtained at 446.5 nm upon excitation at 345 nm. The hydrodynamic size of the particles was <5 nm as determined from DLS analysis.

Gong et al. (2019) reported CQDs synthesis from the polar wood powder using a one-step hydrothermal method. The mixture of about 1 g wood powder, 0.5 g citric acid (CA), and 200 µL ethanediamine (EDA) in 20 mL water was sonicated for 20 min and stirred for 30 min to form a homogeneous suspension. After heating at 200°C for 6 h, a dark brown suspension was formed that was filtered under vacuum conditions in order to remove the precipitates as well as the residues of carbonized wood powder. The purified CQDs were obtained by drying the solution at 30°C. The UV-vis spectroscopy identified the maximum absorbance wavelength for CQDs at 310 nm that was attributed to the n-π* transition. The emission of bright blue light was observed after the excitation (365 nm) of CQDs using a UV beam. The average particle size was 3.4–4.6 nm. The FTIR analysis confirmed the stretching vibrations of C-OH, C-N, C=O, C-H, N-H, and O-H from the peaks obtained at 1,048, 1,180, 1,210, 1,646, 2,992, 3,063, and 3,210 cm^−1^, respectively.

Vandarkuzhali et al. (2017) used an alternative approach to derive fluorescent CDs from a banana pseudo-stem. A mixture of 30 mL of plant extract and 40 mL of ethanol was autoclaved 180°C for 2 h. The unreacted organic moieties present in the newly formed dark brown solution were eliminated by washing with dichloromethane. UV-vis spectra showed an absorption peak of CDs with bright green fluorescence at 365 nm. The strong absorption band was observed at 284 nm due to the π–π* transition corresponding to the carbon core C = C units. The CDs exhibited a typical excitation-dependent PL characteristics with a maximum QY of 48% at the excitation wavelength of 360 nm. TEM images showed a spherical morphology with a homogeneous dispersion of CDs. HR-TEM images identified the crystalline structure of CDs along with its lattice spacing (0.22 nm) and particle size distribution with an average diameter of 2–3 nm. The average hydrodynamic diameter was 2.5 nm with a narrow distribution. XRD analysis confirmed the formation of an amorphous graphite carbon cluster of CDs. The broad absorption peak for O-H (3,315 cm^−1^) stretching vibration of carboxylic acid, C=C, and C-O-C (1,634 cm^−1^ and 1,040 cm^−1^) indicated the presence of sp^2^ hybridized carbon atoms. The highest fluorescence intensity was at 340 nm along with a red-shift of fluorescence maxima with an increase in excitation wavelength. The CDs possessed higher salt tolerance ability (up to 2.0 mol/L).

Shukla et al. (2020) derived CQDs from sandalwood powder employing a hydrothermal carbonization method. The mixture containing 0.5 g sandalwood powder in 50 mL of deionized water was stirred and heated for 8 h at 200°C. The light brown solution obtained after natural cooling was further centrifuged at 15,000 rpm for 20 min, filtered and stored. The prominent absorption peak at 279 nm was revealed from the UV-vis spectrum attributed by the n-π* transition from the C=O functional groups. The fluorescence spectrum disclosed the emission intensity of 458 nm upon excitation with 320 nm of an excitation wavelength for the synthesized CQDs. Around a 12% QY was observed for CQDs when analyzed with respect to quinine sulphate as a reference. The fluorescence lifetime was 8.63 ns. FTIR analysis revealed peaks at 3,423, 2,931, 1,627, 1,400, and 1,075 cm^−1^ assigned to the OH and NH stretching, CH stretching, NH bending, OH bending, and C-O/C-N stretching, respectively. The amorphous nature of the surface of the CQDs was identified from XRD analysis. The quasi-spherical CQDs with a 3.5 nm size was revealed by TEM while the zeta potential was −13 mV.

Thambiraj and Shankaran (2016) synthesized CQDs from a sugarcane bagasse pulp that was dried for 6 days in sunlight before combustion at 60°C. The carbon formed (2 g) was further added to 200 mL toluene and stirred continuously at room temperature for 24 h for complete dispersion. After sonication for 1 h and centrifugation at 10,000 rpm for 30 min at room temperature, the supernatant was collected and diluted with ethanol. UV-vis spectral studies revealed the absorption peak of CQDs at 283 nm which was mainly contributed by the π-π* transition of aromatic sp^2^ hybridization. The CQDs exhibited various emission peaks of 327, 338, 408, and 438 nm at a 283 nm excitation wavelength. The FTIR spectrum detected the absorption peaks for C=C stretching vibration (2,985 and 2,813 cm^−1^), hydrogen bonds (3,440 cm^−1^), and C=O groups (1728 cm^−1^). The HR-TEM analysis revealed small spherical shaped particles with an average size of 4.1 ± 0.17 nm. The EDS spectrum confirmed 87.18% C in the CQDs. The small size of the CQDs with a spherical structure along with 3–5 nm of average diameter and a surface roughness of 5 nm were established from atomic force microscope (AFM) images. The QY of CQDs was 18.7% at 283 nm of an excitation wavelength.

Shen et al. (2017) investigated CDs synthesis from sweet potato. The fluorescence CDs were fabricated by heating the mixture of crushed sweet potato at 80°C for around 3 h under stirring conditions which was followed by centrifugation at 8,000 rpm for 20 min. The supernatant was further heated for 18 h at 180°C followed by cooling it down to room temperature. The dark colored solution obtained was further centrifuged for15 min at 10,000 rpm and filtered. The resulting CDs were dialyzed in deionized water for 48 h. The UV-vis spectrum showed an absorption peak at 266 nm associated with the π-π* transition of C=C. The highest emission peak of 442 nm was obtained upon excitation at 360 nm. The shifting in emission peaks from 406 to 486 nm was noted with an increase in excitation wavelength from 300 to 410 nm. The FTIR spectrum revealed peaks at 3,291, 2,925, 1,695, 1,608, 1,388, 1,145, and 1,022 cm^−1^ which were attributed to -OH stretching, C-H stretching, C=O, C=C stretching vibrations, C-H bending, C-OH stretching, and C-O stretching vibrations, respectively. The TEM analysis indicated a spherical structure and well-separated CDs. The size of CDs was in a range of 2.5–5.5 nm, the average diameter being 3.39 nm.

Mewada et al. (2013) fabricated biocompatible CDs from a *Trapa bispinosa* peel extract. 50 gm of a *T. bispinosa* peel was crushed and added to 500 mL distilled water which was followed by centrifugation to obtain a light pink color extract. C-dots were synthesized by refluxing 100 mL of a *T. bispinosa* peel extract at 90°C for 2 h until formation of a greenish brown colored solution. Further, enhancement in the fluorescent property of CDs was carried out by centrifugation at 5,000 rpm for 20 min and suspension in 5 mL 1 N NaOH. After centrifugation, the CDs were purified by dialysis of a 3 mL solution against nano pure water for 24 h which resulted in the formation of a yellowish solution exhibiting intense green fluorescence under UV light. The observed fluorescence in CDs was due to the recombination of electron-hole pairs from oxygen-bearing functional groups and atomic impurities. UV-visible spectroscopy showed a redshift after 30 min followed by a blue shift after 120 min indicating the complete formation of the CDs. The broad peak and shoulder peak were at 538 nm and 875.61 nm, respectively, which indicated the slow formation of the CDs. The highest intensity of CDs was obtained at 450 nm. HR-TEM images showed spherical CDs with a 5–10 nm size. XRD analysis revealed the graphite nature with a turbostratic carbon structure. FTIR analysis showed absorption peaks at 2,925 cm^−1^ and 2,860 cm^−1^ that were attributed to the stretching vibration of C-H due to the methyl or methylene group associated with the aliphatic hydrocarbons which were present in the plant peel extract. The absorption peak at 3,394 cm^−1^ and 1,648 cm^−1^ were due to O-H and -C=C- stretching, respectively. The role as a linker for the attachment of the therapeutic moieties was fulfilled by -CH_3_ and -OH groups present on the CDs.

Meena et al. (2019) synthesized Ayurvedic based CQDs from different plant sources like *Azadirachta indica*, *Ocimum tenuiflorum*, and *Tridax procumbens* that were labelled as CQDs-1, CQDs-2, and CQDs-3, respectively. About 100–120 mg of carbon ash (from natural sources) was added to 25 mL concentrated H_2_SO_4_ and HNO_3_ followed by sonication for 2–4 h and stirring for a period of 1 day. Precipitates of salt were formed by slow stirring for about 40 h in an ice bath. UV-vis spectroscopy analysis of CQDs identified two maximum absorption peaks at 218 and 315 nm which were attributed to the π-π* transition of C=C and n-π* transition of C=O, respectively. FTIR analysis gave peaks for different functional groups at 3,324–36141, 900–1,026, 1,500–1,600, 2,819 and 2,889 cm^−1^ revealing O-H, -OH, C=O, N-H bending, and -C-H stretching vibrations, respectively. PL spectra revealed an emission wavelength of 518 nm for 430 nm of excitation wavelength. The size range of particles was 6–10 nm along with the average size of 10 nm of CQDs as identified from the TEM results.

### 3.7 Graphene quantum dots

Tade and Patil (2020) synthesized fluorescent graphene quantum dots (GQDs) employing a one pot hydrothermal synthesis method using bamboo timber waste (Bf). Cellulose nanocrystals (CNCs) were prepared from bamboo timber fibers (Bf) by mixing 500 mg dried Bf into 50 mL (60%) H_2_SO_4_ solution followed by 30 min stirring at 70°C. Then, 70 mL ice-cold water was added into the solution which was neutralized using 5 M NaOH followed by washing at 20,000 rpm in order to obtain a dry powder denoted as Bf-CNCs. Bf-GQDs were fabricated by dispersing 200 mg Bf-CNCs in 20 mL distilled water followed by 20 min of sonication. Further, the mixture was autoclaved at 180°C for 8 h followed by cooling at room temperature. The resulting dark-brown dispersed solution was sonicated for 10 min at room temperature and filtered using a 0.22 µm microporous membrane and dialyzed (12,000 kDa). The outer yellowish-brown solution (Bf-GQDs) was collected and freeze-dried. A green fluorescence at 254 nm and strong fluorescence at 365 nm were revealed from the UV-vis spectroscopy. The increase in fluorescence intensity and the hyperchromic shift were noted after dialysis. The Bf-GQDs showed a pale brown color in the presence of natural light whereas a uniform color either at 254 nm (pale greenish) or 365 nm (strong blue) was seen. The typical absorption peaks of Bf-GQDs were detected at 263.6 nm (π-π* transition) and 321.4 nm (n-π* transition). An average diameter of Bf-GQDs was 9.8 nm along with a −16.65 mV zeta potential. The PL of Bf-GQDs varied with the nature of the solvent and concentration. Bf-GQDs showed a maximum excitation wavelength at 410 nm which suggested a higher PL. The PL QY was 38.9% for Bf-GQDs. SEM images showed the coarse and dispersed form of the Bf-GQDs. Elemental analysis of Bf-GQDs revealed an atomic percentage of C (41.10 wt%) and O (56.9 wt%), confirming the transformation of Bf into a graphite structure. FTIR analysis revealed an absorption peak at 1,637 cm^−1^, 3,403.57 cm^−1^, 1,443 cm^−1^, and 1,179 cm^−1^ for carboxyl, hydroxyl, carbonyl, and epoxy groups, respectively. HR-TEM images showed the even distribution and spherical-shaped crystal nature of Bf-GQDs along with a uniform size range (2–8 nm) and 2.8 ± 0.5 nm average size of the particle. Around a 0.27 nm distance was evaluated between the lattice fringes. The selected area diffraction pattern (SAED) demonstrated the ring pattern of Bf-GQDs which also suggested the crystalline (80.9%) nature of Bf-GQDs that was well in agreement with the XRD analysis. A monotonic elevation in the emission intensity of Bf-GQDs was observed with an increase in pH whereas at higher alkaline conditions the Bf-GQDs showed aggregative behavior. However, at stronger acidic conditions, the fluorescence property of the Bf-GQDs were completely compromised.

Tak et al. (2020) utilized flowers of *Clitoria ternatea* (ct) for the formation of GQDs. The synthesis was carried out from an ethanolic flower extract by a one-pot microwave assisted green synthesis method. Initially, the ethanolic extract of the ct flower was centrifuged at 8,000 rpm for 10 min to obtain the purified extract. The extract was filtered and heated for 5–10 min. Further, the slurry was heated for a period of 24 h at 60°C in order to produce the powdered form of ctGQDs. The absorption studies of ctGQDs via UV-vis spectroscopy identified the surface plasmon resonance (SPR) spectrum at 320 nm which was attributed to the electronic transition of the carbon structure (n-π*). The average hydrodynamic size of particle was 10 ± 1.3 nm while the PDI was 0.354 ± 1.8 which confirmed the formation of small-sized homogenously distributed particles. Spherical shapes 10–20 nm in particle size of the synthesized ctGQDs were revealed by field emission SEM (FESEM). This spherical morphology with a 10–20 nm particle size was also confirmed by TEM analysis. The occurrence of carbon and oxygen containing groups in ctGQDs was identified by XPS. The surface area of the particle was 412 m^2^/g.

Kumawat et al. (2017) fabricated bright red-luminescent graphene quantum dots (GQDs) from *M. indica* (mango) leaves. The reaction mixture was initially prepared by dipping tiny pieces of mango leaves in absolute ethanol which was followed by stirring and centrifugation at 8,000 rpm for 10 min. The slurry was microwaved for 5 min and was dispersed in absolute ethanol that was filtered and dried at 65°C for 24 h to produce a dried powder of mGQDs. The absorption peak attributed to the n-π* transitions (carbon structure) was observed at 280 nm via UV-vis spectroscopy. Peaks of -OH bending vibrations, carbonyl groups, C-C, C=C, and C-H stretching vibrations were confirmed from FTIR analysis. PL spectra showed excitation wavelengths from 300 to 500 nm and the highest emission peak at 400 nm. An excitation-independent fixed emission at 680 nm was noted that indicated excellent photostability and lifetime of the GQDs. The particle sizes ranged from 2 to 8 nm with a 0.32 nm lattice distance as determined from the field emission gun transmission electron microscope (FEG-TEM), as shown in Figures 3C, D.

Roy et al. (2015) fabricated GQDs from the neem leaf extract by hydrothermal treatment at 300°C for 8 h followed by centrifugation at 25,000 ×g for 20 min, and filtration through a dialysis membrane. The resulting pure GQDs (12 mg/mL) exhibited an absorption peak at 265 nm that was attributed to the π-π* transition of aromatic sp^2^ domains of GQDs. The PL spectral studies revealed an emission wavelength of 440 nm upon excitation with 365 nm. The highly dispersed GQDs had spherical shapes with sizes of 5.0 ± 0.4 nm as detected by TEM. XPS spectra confirmed the occurrence of carbonyl, hydroxyl, and carboxylic acid groups while the FTIR spectra detected stretching peaks for C-O, C-H, C=O, and O-H groups at 1,129, 1,420, 1,586, and 3,200–3,500 cm^−1^, respectively.

Anooj and Praseetha (2019) investigated GQDs synthesis from nutmeg seeds. The reaction mixture containing 0.1 g seeds and 1 mL hydrazine hydrate dissolved in 10 mL water was sonicated for 30 min. The mixture was heated between 150°C and 200°C for 6–10 h, cooled to 37°C, and drained using a microporous membrane. The resulting black-colored GQDs were dried at 80°C and the yield was 33%. The UV-vis spectrum revealed the highest absorption by GQDs at 320 nm which indicated a face-centered cubic polycrystalline structure with a 6.1 nm size.

Centeno et al. (2021) synthesized GQDs using fresh cladodes of *Opuntia* sp. A 500 mg extract of *Opuntia* sp. was added to the mixture of ethylene glycol (10 mL) and ultrapure water (2.5 mL). Further, magnetic stirring of the reaction mixture was carried out for 24 h followed by 4 min of microwave heating. GQDs formation was confirmed from the color change from green to brown. The resulting GQDs were then centrifuged for 10 min at 3,000 rpm, dialyzed for 24 h, washed with ethanol and acetone, and dried. TEM analysis revealed a dispersed non-aggregated quasi-spherical nanostructure with an average size of 2.6 ± 0.63 nm and lattice spacing of 0.21 ± 0.002. The Zeta potential was 33 mV that rendered high stability to the GQDs. Elemental analysis revealed the composition of GQDTs as 77% carbon and 18% oxygen attributed to C=O and C-O-C functional groups. FTIR analysis indicated a C-H stretching band at 2,946–2,878 cm^−1^. The highest absorption of C=C (π-π* transition) present in the aromatic ring of the graphene backbone was observed at 263 nm. The maximum emission spectrum for the GQDs appeared at 334 nm upon excitation at 253 nm.

Khose et al. (2021) greenly developed novel red-fluorescent GQDs from the leaves of guava (*Psidium guajava*). The fresh leaves were washed, chopped, and blended using a mixer to obtain a fine powder. A 10 g leaf powder was added into 100 mL of absolute alcohol followed by stirring for 3 h. The extract was autoclaved at 180°C for 18 h in a muffle furnace followed by filtration to obtain GQDs. Optical studies of GQDs via UV-vis spectroscopy revealed a strong peak at 220 nm that was attributed to π-π* transitions of electrons in molecular orbitals of sp^2^ graphene, a shoulder band at 415 nm that was attributed to n-π* absorptions of carbonyl, -OH, and other functional groups, and a long tail extending to 700 nm. The highest emission intensity (fluorescence) by GQDs was observed at a 400 nm excitation wavelength. FTIR spectroscopy revealed that O-H groups (3,321 cm^−1^), C-H stretching (2,972 cm^−1^), C=C groups (1,660 cm^−1^), C-H bending (1,447 cm^−1^), and C-O (1,100 and 1,045 cm^−1^) groups were associated with the GQDs. The abundance of C and O at a 3.11 ratio was revealed from the XPS analysis.

## 4 Mechanism of synthesis

It is important to understand the underlying mechanism behind the biogenic synthesis of QDs using algae and plants. Several studies have put forth various hypotheses based on their findings on how the metabolites play an important role in the synthesis and stabilization of QDs. This section discusses the mechanisms for QDs biosynthesis in detail.

### 4.1 Phycogenesis

Zhang et al. (2018) described the possible mechanism for intracellular CdSe QD fabrication by the use of *C. pyrenoidosa* and *S. obliquus*. The internalization of Na_2_SeO_3_ into the cells of microalgae was followed by its enzymatic reduction into the selenium precursor by the act of glutathione (GSH) in the presence of NADPH. These precursors were further combined with the Cd(SG)_2_ under the catalysis of relative reductase which finally resulted in CdSe QD formation. Zhang et al. (2022a) suggested the plausible mechanism behind the synthesis of CDs from *C. pyrenoidosa*. Initially, the substrates (such as carbohydrates, lipids, and proteins) were hydrolyzed into non-reducing sugars, fatty acids, and amino acids followed by the Amadori rearrangements of sugars and amino acids in order to form ketones and amines. Amidation reactions among amino acids and fatty acids resulted in the formation of amides and long-chain alkanes. In the next stage, the Amadori products along with sugars and amino acids were subjected to cyclization, rearrangement, deoxidization, or denitrification in order to generate aromatic products. Those products were further copolymerized to form CDs. Thus, the reactions such as hydrolysis, Amadori rearrangements, cyclization, and polymerization resulted in the successful formation of CDs. Guo et al. (2021) speculated the mechanism behind the formation of CDs from *C. pyrenoidosa*. The formation of CDs began initially by hydrothermal carbonization of *C. pyrenoidosa* cellulose and hemicellulose as well as proteins which resulted in the formation of carbohydrates and amino acids followed by their dehydration and decarboxylation reaction. The resulting product was further subjected to the Millard reaction, deamination polymerization as well as carbonization and nucleation which resulted in the formation of CDs.

### 4.2 Phytogenesis

Malavika et al. (2021) identified a bottom-up approach for the synthesis of CQDs from *A. vera* extracts. The possible mechanism behind the formation of CQDs using the microwave-assisted reflux method was the conversion of smaller carbon units (precursor molecules) into fluorophores due to the thermal energy generated in the microwave and further polymerization (due to heating), condensation, and carbonization of fluorophores which led to CQD formation. Shivaji et al. (2018) gave a plausible mechanism for CdS QDs synthesis where the addition of CdSO_4_ in the plant extract resulted in the binding of the Cd^2+^ ions with the plant-mediated proteins resulting due to metallic stress. Further, the addition of Na_2_S resulted in the formation of S^2+^ ions which attached to the plant-mediated cadmium proteins and formed the CdS QDs.

In another study, Singh et al. (2018) described the mechanism for ZnO QDs by reacting the *E. alba* leaf extract and zinc acetate. The first stage was activation, where the reduction of Zn^2+^ions to Zn^0^ atoms was carried out due to the bioreducing agents like flavones, quinones, and organic acids present in the leaf extract. The Zn^0^ atoms were further converted to ZnO in the presence of dissolved oxygen (O_2_) followed by nucleation of ZnO. The enhancement in thermodynamic stability of QDs due to spontaneous formation of larger particles from the bonding of smaller particles was observed in the second or growth stage. The third stage or termination/stabilization, was associated with the ZnO QDs attaining the final spherical shape with the most favorable conformation administered by the phytochemicals from the leaf extract which acted as capping agents for stabilizing and preventing aggregation of ZnO QDs.

Wang et al. (2021) illustrated the mechanism behind the synthesis of CdS QDs where the functional group B was formed due to the interaction between the sulfur atom present in the thioacetamide and the organic group. The electronic autoclave provided stable conditions in order to ensure the presence of sufficient energy required for bond formation and breaking. Eventually small sized water soluble CdS QDs were formed after the interaction of Cd of group A with the S of group B which were further wrapped with the organic group from the opposite end and were further stabilized in a colloidal environment created by starch.

**FIGURE 4:**
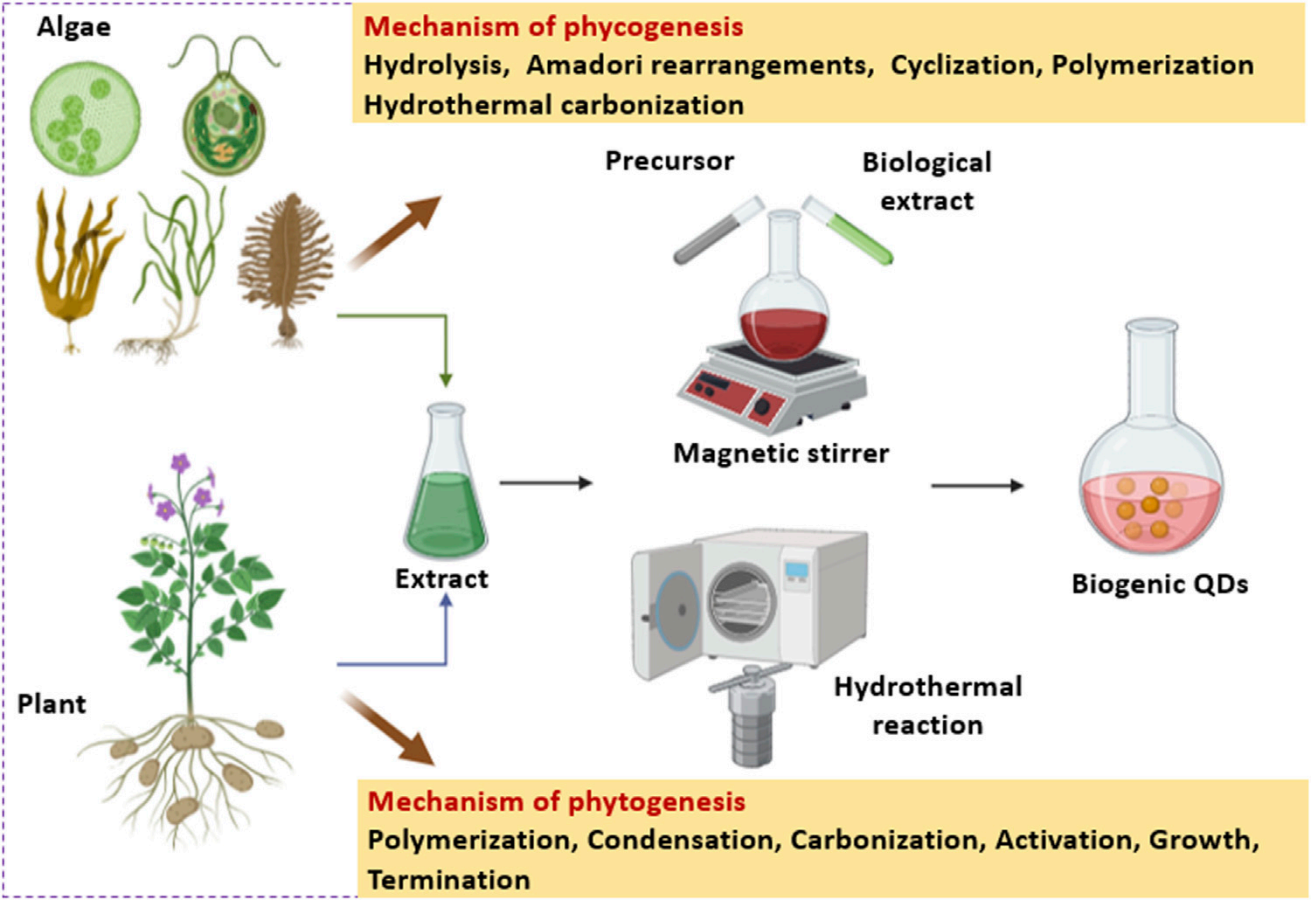
Schematic representation of phycogenesis and phytogenesis of QDs with their synthesis mechanisms.

In summary, both phycogenic and phytogenic models for the biofabrication of QDs involve nucleation, maturation, and stabilization of nanoparticles as schematically represented in Figure 4. This sequential process is guided by physical parameters that include concentration of the precursor, temperature, pH, and reaction time. These conditions can be rationally modulated for obtaining tunable physicochemical and optoelectronic properties of the resulting QDs.

## 5 Application

**FIGURE 5:**
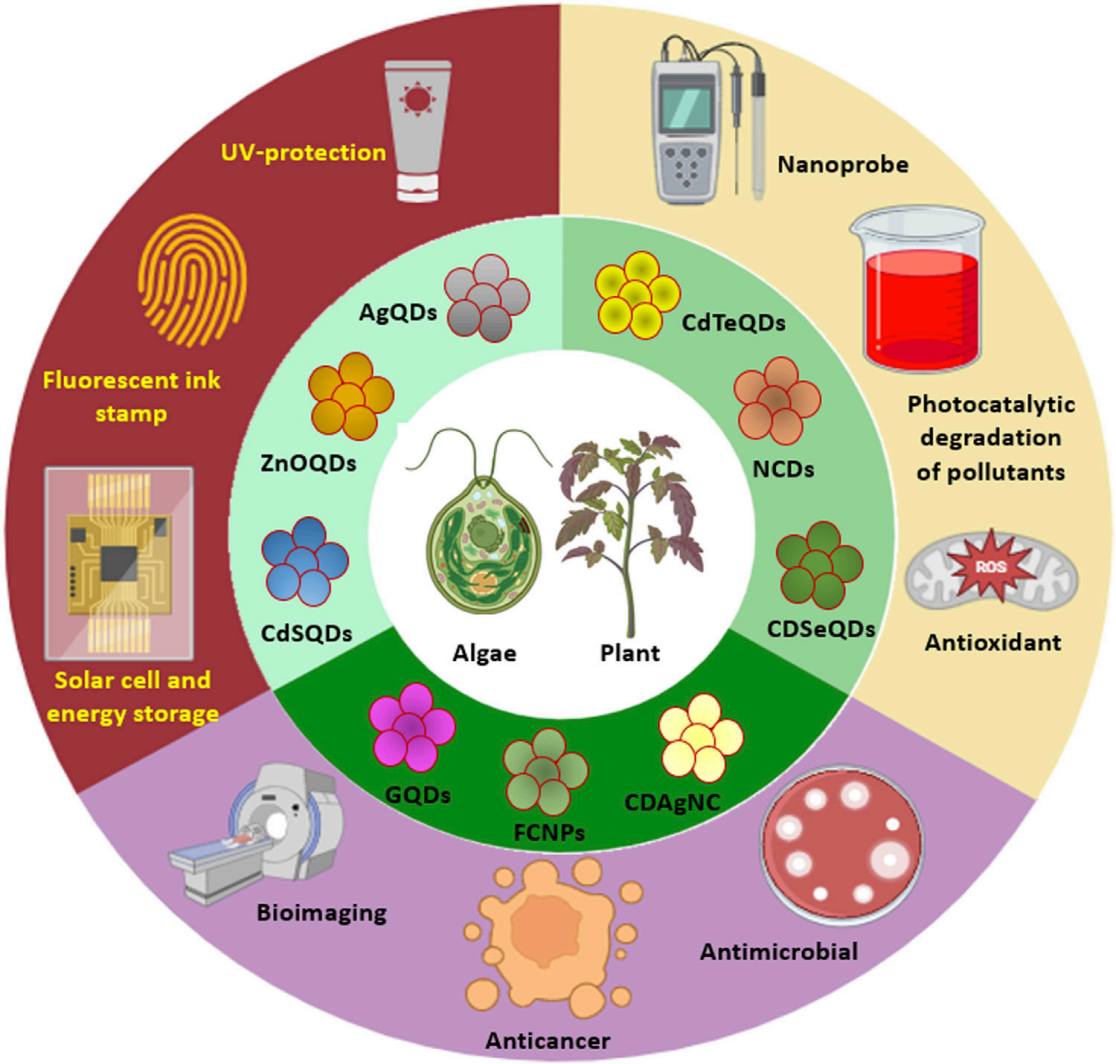
Applications of QDs synthesized using algae and plants.

The biogenic QDs fabricated using algae and plants have diverse functions that include sensing, photocatalysis, diagnostics and therapy. This section gives a detailed account of various applications of the phyco- and phyto-synthesized QDs which are pictorially illustrated in Figure 5.

### 5.1 Detection

Zhang et al. (2018) employed CdSe QDs derived from *C. pyrenoidosa* and *S. obliquus* in the detection of imatinib. The optimized parameters for the development of a highly sensitive nanoprobe were 100 μg/mL CdSe QDs in solution, pH 3, and ambient temperature. The fluorescence intensities of QDs (460 nm) showed a reduction with an increase in imatinib concentration. Except for Cu^2+^, no interference was observed for imatinib detection in the presence of other metals even at their higher concentrations. A recovery range of 99%–101% was noted at different concentrations of imatinib in solution even in presence of interfering ions suggesting high specificity and sensitivity of the nanoprobe.

In another study, Li et al. (2019a) detected Fe(III) ions using N/S-CDs derived from *D. salina*. The addition of 4 mmol/L EDTA to the mixture of different metals in the presence of N/S-CDs showed a significant reduction in fluorescence intensity particularly in the case of Fe(III). The concentration of Fe(III) showed a negative linear relationship with respect to fluorescence intensity. The Stern–Volmer quenching constant (K_SV_) was 6.43 × 10^3^ L/mol at 25°C and 6.60 × 10^−3^ L/mol at 45°C. The fluorescence was quenched within 6.42 ns in the absence of Fe(III) and 6.43 ns in the presence of Fe(III); about 91.60%–117.3% Fe(III) was recovered.

Singh et al. (2019) tested the Hg (II) and Cr (VI) sensing ability of the CDs derived from *D. salina*. About 50 µL of a salt solution (2 mM) was added to a 1 mL CDs solution at pH 6.8. Apart from different ions, Hg (II) and Cr (VI) possessed a greater ability to quench the blue fluorescence of CDs suggesting their promise in sensing metals. Zhang et al. (2022a) studied the Fe^3+^ detection ability of CDs derived from *C. pyrenoidosa*. The metal ions quenched the PL intensity of the particles. The limit of detection (LOD) of the metal ions was 0.55 nmol/L. Placido et al. (2019) evaluated the use of MAB-CDs derived from microalgal biochar in the sensing of metal ions such as: Pb^2+^, Cu^2+^, Cd^2+^, and Ni^2+^. At a 2 mM Pb^2+^ concentration, about 30% fluorescence was quenched and a linear correlation was observed between the Pb^2+^ concentration and fluorescence reduction from 0.012 µM to 5 µM. Similarly, at a 20 mM Cu^2+^ concentration, 60% quenching in the fluorescence with a linear correlation from 1 μM to 10 mM was noted. Cu^2+^ exhibited the lowest quenching of fluorescence with the highest reduction in fluorescence being less than 10%. The variation in quenching was observed from 0.0125 µM to 50 μM, above which the fluorescence reduction was saturated at 8%. In the case of Cd^2+^, a linear correlation between metal ion concentration and fluorescence reduction was noted between 0.01 µM and 1 µM. About a 50% reduction in fluorescence was observed at a 20 mM Ni^2+^ concentration and a linear correlation with fluorescence reduction was noted between 3 μM and 10 mM. Neutral and slightly alkaline pH was optimal for heavy metal quenching.

Liu et al. (2015) analyzed the metal-sensing ability of N-CDs synthesized from AA obtained from brown algae. At a 5 × 10^−3^ M concentration of Fe^3+^ ions, the PL quenching efficiency was 82.7%, the emission wavelength being 450 nm. Xu et al. (2017) tested the metal ion sensing ability of CNDs derived from *E. prolifera* under acidic conditions (pH 6). The concentration in the range of 0–1.7 mM of Fe^3+^ was used for the analysis. The LOD for Fe^3+^ by CNDs was around 0.5 µM. With an increase in Fe^3+^ concentration, the quenching of the PL also increased which suggested the conversion of bright blue light (UV) to a pale blue color. Arumugam and Kim (2018) applied CQDs obtained by a one step hydrothermal process of broccoli to detect Ag^+^ (from 0 to 600 µM). Strong emissions (450 nm) were exhibited by CQDs and the quenching of probe PL intensity upon the addition of different Ag^+^ concentrations was observed. The intensity and Ag^+^ concentration (0–600 µM) exhibited a linear relationship having a correlation coefficient *R* ^2^ = 0.992 as derived using the Stern–Volmer equation. CQDs sensitivity for Ag^+^ ion detection was confirmed from an LOD of 0.5 µM calculated at 3 ratios of signal-to-noise.

Centeno et al. (2021) used GQDTs synthesized from *Opuntia* sp. in order to detect phytic acid (PA) and phosphates. A linear behavior along with *R* ^2^ = 0.98 and 0.85 was observed while detecting dibasic phosphate and monobasic phosphate, respectively. The asymptotic behavior was indicated from detection of PA with an *R* ^2^ = 0.91. The detection of PA and phosphates was carried out at a pH 8.8 to increase the aggregation and quenching effect. The highest quenching of 53%, 26%, and 21% was observed for PA, sodium phosphate monobasic (SPMB) and sodium phosphate dibasic (SPDB), respectively. Arumugham et al. (2020) employed the CQDs synthesized using leaves of *C. roseus* as fluorescence probes for multi-ion detection. Different ions such as K^+^, Ca^2+^, Mg^2+^, Cr^3+^, Mn^2+^, Fe^2+^, Co^2+^, Ni^2+^, Cu^2+^, Ag^+^, Zn^2+^, Cd^2+^, Hg^2+^, and Al^3+^ were checked for detection using CQDs. Compared to the other ions, Fe^3+^ and Al^3+^ ions gave strong fluorescence intensity. The decrease from 5.48 ns to 4.99 ns in fluorescence lifetime (FL) was observed after the addition of Fe^3+^ in CQDs while an increase from 5.48 to 5.55 ns was noted after the addition of Al^3+^ to CQDs. LOD of about 0.3 µM was noted for Fe^3+^ and of about 0.5 µM for Al^3+^, respectively.

Khose et al. (2021) utilized the emission property of G-GQDs synthesized from a guava leaf as a fluorescence turn-off probe for the detection of the selective metal Hg^2+^ in an aqueous solution. The LOD of Hg^2+^ by G-GQDs was around 82 µM and they also possessed a large dynamic range of up to 0.38 mM. The detection was selective and very sensitive even under low pH conditions (pH 2). Wang et al. (2021) analyzed the application of CdS QDs synthesized from potato extract for the detection of Ag^+^ ions. About a 1–100 mg/L Ag^+^ solution was used for the experiment. The decrease in fluorescence intensity along with the quenching effect and red shifting of CdS QDs with respect to the increment in Ag^+^ concentration was observed after treating the QDs with the Ag^+^ solution. The straight-line correlation coefficient *R* ^2^ = 0.987 suggested the efficiency of CdS QDs for the detection of Ag^+^ ions. Raja and Sundaramurthy (2021) analyzed the sensing of Fe^3+^ ions using the CQDs synthesized from a betel leaf. It was observed that at a linear range of 0–150 nM, a good linear relationship with *R* ^2^ = 0.9944 was noted for the sensing of Fe^3+^ via CQDs and the quenching mechanism was attributed to the charge transfer of carboxylic group associated with CQDs to Fe^3+^.

Shahshahanipour et al. (2019) used CDs synthesized from a *L. inermis* plant for the sensing of the anticancer drug methotrexate. A nearly linear behavior with *R* ^2^ = 0.987 of fluorescence quenching was in the range of 0.02–18 μmol/L with an LOD of 7 nmol/L. Shen et al. (2017) used fluorescence CDs developed from the sweet potato for sensing Fe^3+^ ions. An inverse relation between Fe^3+^ ion concentration and fluorescence intensity of CDs was noted. A good linear relation was observed with an increase in Fe^3+^ from 1 to 100 µM with an *R* ^2^ value equivalent to 0.9967. The LOD for Fe^3+^ was very low and that provided strong evidence that the CDs were promising for the detection of even a trace amount of Fe^3+^. The lowest LOD was at 0.32 µM of Fe^3+^. Tade and Patil (2020) used Bf-GQDs synthesized from bamboo timber waste for the detection of curcumin. The fluorescence emission spectra were recorded at 410 nm when the curcumin concentration was in a range between 0 and 200 µM. The curcumin concentration and fluorescence of Bf-GQDs showed an inverse relationship. The linear relationship between the concentration of curcumin and quenching efficiency (*R* ^2^ = 0.9758) was in the range from 3.0 to 1.5 µM. Bf-GQDs showed the PL quenching kinetics towards the curcumin with an LOD of 30.0 nM/L as evaluated by the Stern–Volmer plot. The quenching efficiency (%) of curcumin towards Bf-GQDs was 77.83. Vandarkuzhali et al. (2017) investigated the Fe^3+^ sensing potential of CDs synthesized from the pseudo-stem of the banana plant. The drastic reduction in the fluorescence intensity of CDs (0.1 mg/mL) was observed with the addition of Fe^3+^ (100 µM). Even after the addition of the most common interfering ions (each at a 50 µM concentration) along with Fe^3+^ (50 µM), there was no significant change in the relative fluorescence of CDs unlike Fe^3+^ alone.

### 5.2 Degradation

Li et al. (2019a) studied the photocatalytic activity of N/S-CDs fabricated using *D. salina* towards methylene blue (MB) and methyl violet (MV) dyes. The rate of catalytic degradation of dyes by N/S-CDs was 2 times higher compared to the undoped CDs. This significant difference in the catalytic performance was mainly due to the variation in the surface functional groups of N/S-CDs (comprising hydroxyl and carboxyl) and CDs (containing amino and hydroxyl). It was speculated that the carbonyl groups present in the catalytic active site of N/S-CDs attributed to the superior photocatalytic degradation of dyes. Malavika et al. (2021) tested the photocatalytic activity of CQDs fabricated from the *A. vera* extract for anionic dye eosin yellow (EY) remediation. The photocatalytic efficiency was 98.55% and 100% at 80 min and 100 min of contact time, respectively. The remediation of the dye was attributed to the oxygen-containing functional groups and delocalized π electrons present on the CQDs surface which effectively interacted with the EY molecule. Zaib et al. (2021) used the CDs from leaves of *E. cardamomum* (EC) for the photocatalytic degradation of Congo red (CR) and methylene blue (MB) dye. The maximum CR dye degradation occurred at pH 4 when 5 mL of CDs reacted with 5 ppm of CR dye for 50 min.

### 5.3 Antioxidant activity

Li et al. (2019a) analyzed the radical scavenging activity by employing N/S-CDs derived from *D. salina*. At a 500 μg/mL particle concentration, the highest antioxidant activity against 1,1-diphenyl-2-picrylhydrazyl (DPPH) free radicals was 67.3%. Scavenging of the hydroxyl radical was 71.2% at a 400 μg/mL concentration. Wang et al. (2019) studied the radical scavenging potential of CDs derived from *E. prolifera* against natural phenolic compounds. An increase in concentration of 1,4-benzoquinone significantly increased the quenching effect. At a 1M CA concentration, the fluorescence of CDs was reduced by 78%. The QY of particles was 14% ± 0.2% which was reduced to 2.2% upon treatment with catechin and up to 4%–5% upon treatment with CA, caffeic acid, and dopamine.

Arumugham et al. (2020) investigated the antioxidant activity of CQDs obtained from the leaves of *C. roseus* using the DPPH assay which ranged from 50 to 300 μg/mL. The antioxidant activity was concentration dependent. However, a saturation in the antioxidant activity (67.3%) for CQD was attained beyond 200 μg/mL. Anooj and Praseetha (2019) reported the lowest DPPH activity of 12.48% at 125 μg/mL GQDs synthesized from nutmeg. Likewise, Naik et al. (2020) demonstrated dose-dependent scavenging of DDPH radicals by AAPCDs developed from *A. paniculata.* The quenching in the AAPCDs fluorescence was observed after its addition to a methanolic DPPH solution.

### 5.4 Antibacterial activity

Calangian et al. (2018) analyzed the antibacterial activity of FCNPs fabricated from *C. vagabunda* against *Staphylococcus aureus* and *Escherichia coli*. The largest zone of inhibition (ZOI) was 12 mm against *S. aureus* at a 1:1 concentration of particles in 40% (v/v) ethanol. The ZOI in the case of *E. coli* was around 9 mm at a 1:2 ratio of NPs in a 40% (v/v) ethanol concentration. Suresh et al. (2023) evaluated the antibacterial activity of CD-Ag NCs from *C. hornemanni* against *Klebsiella pneuomoniae*, *E. coli*, *Pseudomonas aeruginosa*, *B. subtilis*, MRSA, and *S. aureus*. At 50 μg/mL, the NCs exhibited the highest ZOI equivalent to 17.3 mm (*K. pneumoniae*), 16.6 mm (*E. coli*), 18 mm (*P. aeruginosa*), 16.3 mm (*Bacillus subtilis*), 16.3 mm (MRSA), and 17.6 mm (*S. aureus*).

Kim et al. (2022b) studied the antibacterial effect of CNDs-ZnO nanoconjugates fabricated using *S. horneri* against *E. coli*, *Salmonella typhimurium*, *Vibrio alginolyticus*, *B. cereus*, and *S. aureus*. The maximum ZOIs were 22 ± 3 and 22 ± 2 mm (*E. coli*), 21 ± 2 and 20 ± 2 (*S. typhimurium*), 35 ± 6 and 35 ± 8 mm (*V. alginolyticus*), 17 ± 2 and 16 ± 2 mm (*B. cereus*), and 25 ± 4 and 25 ± 4 mm (*S. aureus*). Under UV light exposure, the enhancement in the ZOI were noted against *E. coli* (22 ± 3 mm and 21 ± 2 mm), *S. typhimurium* (21 ± 2 mm and 21 ± 2 mm), *V. alginolyticus* (36 ± 9 mm and 37 ± 10 mm), *B. cereus* (18 ± 4 mm and 20 ± 2 mm), and *S. aureus* (26 ± 4 mm and 27 ± 3 mm). The MIC of the CNDs-ZnO were 362.5, 725.0, 362.5, 90.63, and 181.25 μg/mL against *E. coli*, *B. cereus*, *S. typhimurium*, *S. aureus*, and *V. alginolyticus*, respectively. Singh et al. (2018) tested the antimicrobial activity of ZnO QDs synthesized from the *E. alba* leaf extract against *E. coli* using an agar well diffusion technique. Treatment with 50 µL of ZnO QDs resulted in a clear ZOI of 15.69 mm which was 1.6-fold larger than the wells loaded with zinc acetate (control) showing a 9.63 mm zone of inhibition.

Pisal et al. (2019) reported antimicrobial activity against *P. aeruginosa* and *E. coli* where the maximum ZOIs were 17 mm and 17.5, respectively, at a 150 μg/mL concentration of CeO_2_QDs synthesized from the *A. vera* leaf extract. Oxidative stress was speculated as the underlying mechanism that resulted when the CeO_2_QDs came in contact with the bacterial cell membrane. Anooj and Praseetha (2019) reported the ZOIs against *S. aureus*, *E. coli*, *P. aeruginosa*, *Salmonella* spp., and *Streptococcus mutans* were 15, 13, 10, 9, and 15 mm, respectively, at 1,000 μg/mL of GQDs synthesized from nutmeg seed. Likewise, Silva et al. (2017) showed a 99.88% inhibition of the initial CFU/mL of *E. coli* ATCC 25992 using 5 and 10 mg/L of the AgQDs synthesized from *E. crassipes*.

**FIGURE 6:**
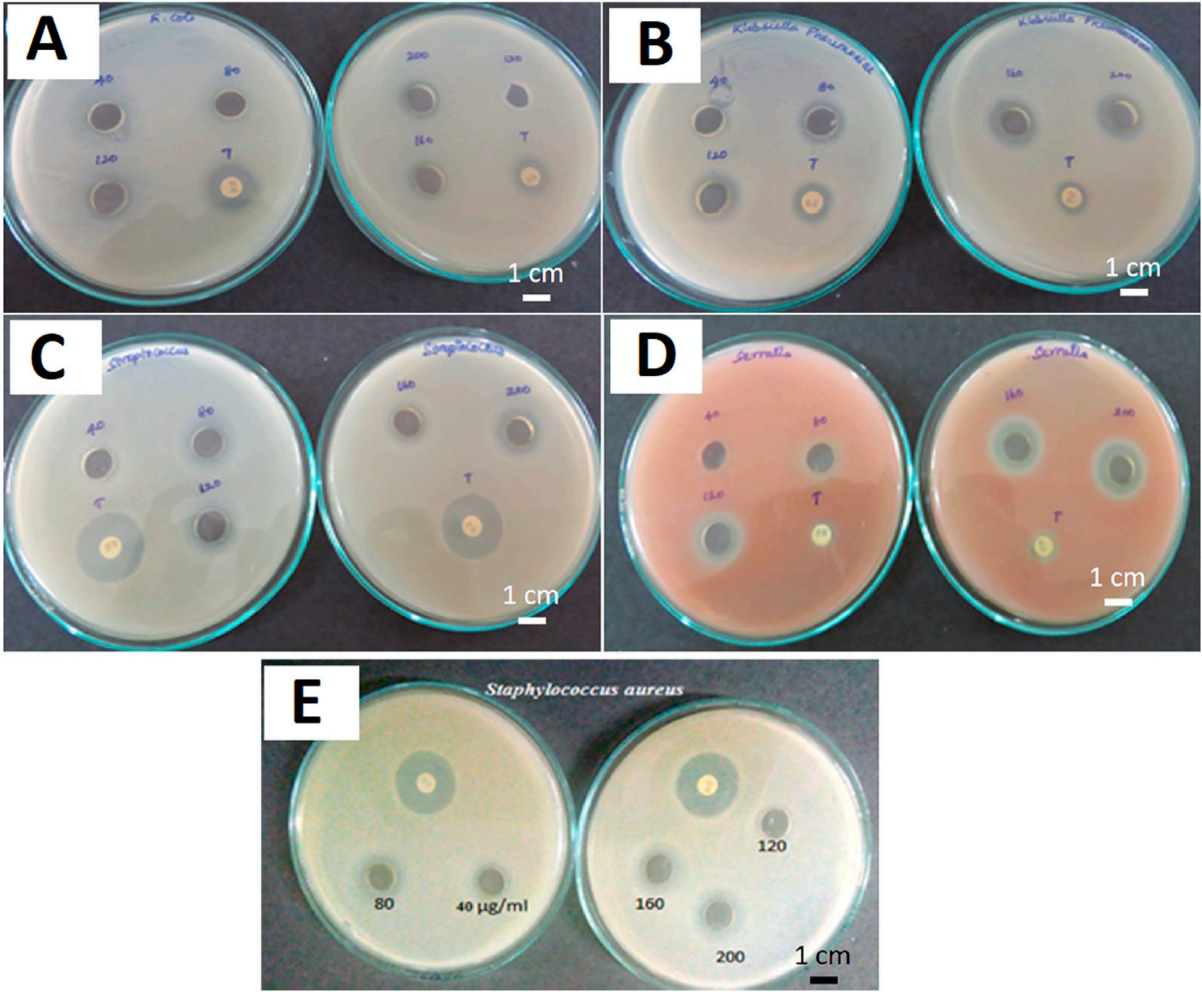
CdS QDs against **(A)** *E. coli*, **(B)** *K. pneumoniae*, **(C)** *S. pyogenes*, **(D)** *S. marcescens*, and **(E)** *S. aureus*. [Note: ampicillin (10 μg) and streptomycin (10 μg) were used as controls in each measurement]. Reprinted with permission from Shivaji et al. (2018). Green-synthesis-derived CdS quantum dots using tea leaf extract: Antimicrobial, bioimaging, and therapeutic applications in lung cancer cells. *ACS Appl. Nano Mater.* 1 (4), 1,683–1,693. doi:10.1021/acsanm.8b00147. Copyright^©^ 2018 American Chemical Society.

Shivaji et al. (2018) demonstrated a ZOI of 21 mm against *Serratia marcescens* using 200 μg/mL CdSQDs synthesized from a tea leaf extract as seen in Figure 6. The ZOI against *S. aureus* was 20 mm. Similarly, Alvand et al. (2019) reported a dose dependent increase in the antimicrobial effect of CdTe QDs synthesized from *F. johannis* plant against Gram-positive bacteria. Shahshahanipour et al. (2019) found that the CDs synthesized from *L. inermis* could inhibit both Gram-positive and Gram-negative bacteria even at a low concentration.

### 5.5 Antifungal activity

Kim et al. (2022b) evaluated the antifungal activity of CNDs-ZnO nanocomposites derived from *S. horneri* against fungal pathogens, namely, *Rhodotorula mucilaginosa*, *Saccharomyces cerevisae*, *Aspergillus flavus*, *Aspergillus niger*, and *Aspergillus terrus*. The particles showed a maximum ZOI of 24 ± 1 and 28 ± 2 mm against *R. mucilaginosa* followed by *A. niger* (22 ± 2 and 23 ± 2 mm), *A. flavus* (14 ± 1 and 15 ± 1 mm), *A. terreus* (12 ± 1 and 13 ± 1 mm), and *S. cerevisiae* (12 ± 1 and 16 ± 1 mm). In a study by Anooj and Praseetha (2019), the GQDs synthesized from nutmeg seeds exhibited ZOIs of 16 mm and 14 mm against *Microsporum gypseum* and *Trichophyton,* respectively, at a concentration of 1,000 μg/mL.

### 5.6 Cytotoxicity

Guo et al. (2017) studied the cytotoxicity of CQDs derived from microalgal powder on human breast cancer (MCF-7) cells by using a 3-[4, 5- dimethylthiazol-2-yl]-2, 5-diphenyltetrazolium bromide (MTT) assay. Cell viability was equivalent to 100%, 95% and 85% when treated with 400 μg/mL, 600 μg/mL, and 85%, of CQDs, respectively. Amjad et al. (2019) showed the anticancer activity of CQDs synthesized from the *Pectinodesmus* sp. strain PHM3 against breast cancer (HCC1954) and colorectal cancer (HCT116) cell lines. The concentration of CQDs at which a 50% growth inhibition (GI_50_) was evident against HCC1954 cells and HCT116 cells were 0.542 ± 0.715 ng/mL and 0.316 ± 0.447 ng/mL, respectively. CDs derived from eutrophic algal bloom exhibited a negligible cytotoxic effect against MCF cells even at a 0.5 mg/L concentration after 12 h. The viability of the cells reduced to 60% upon treatment with a 1 mg/L CDs concentration for the period of 48 h (Ramanan et al., 2016). The toxicity of FCNPs derived from *C. vagabunda* was evaluated via a brine shrimp lethality test. No lethal effect was noted on the brine shrimp upon treatment with a 1:1 to 1:16 ratio of the NPs (Calangian et al., 2018).

The viability of the HEK-293 cells was 80% after 24 h when they were treated with 75 μg/mL of CDs derived from *D. salina* (Singh et al., 2019). The IC_50_ value of the CD-Ag NCs fabricated from *C. hornemanni* against breast cancer cells (MDA-MB-231) as well as noncancerous HEK-293 cells were 12 μg/mL and 18 μg/mL, respectively after 24 h of treatment (Suresh et al., 2023). The Ishikawa cells showed a viability higher than 89% after 24 h when treated with 80 μg/mL of CDs synthesized using *Chlorella*. This suggested their lower cytotoxicity as well as good biocompatibility (Dong et al., 2021). HeLa cells treated with 1 mg/mL CDs fabricated using *E. prolifera* showed a viability higher than 85% after 24 h of incubation (Wang et al., 2019). Similarly, HeLa cells showed 85% viability when treated with 300 μg/mL CNDs derived from *E. prolifera* (Xu et al., 2017). Interestingly, a 50% decrease in viable MCF-7 cells (IC_50_) was detected at 52.2 ± 1.35 μg/mL of CQDs derived from *A. vera* extract (Malavika et al., 2021). 88% MCF-7 cell viability was noted upon treatment with 1 mg/mL CQD from *C. roseus* leaves while the viability of MCF-10a cells was 82% at an identical dosage (Arumugham et al., 2020). The lowest cell viability of 23.3% in HeLa cells was observed when treated with 1,000 μg/mL GQDs synthesized from nutmeg seeds (Anooj and Praseetha, 2019). Treatment with 12.5 mg/mL and 25 mg/mL of CdSQDs synthesized using hairy roots of *R. sativus* L exhibited 20% and 50% cell viability against MCF-7 and AGS cancerous cells, respectively (Gholami et al., 2020).

**FIGURE 7:**
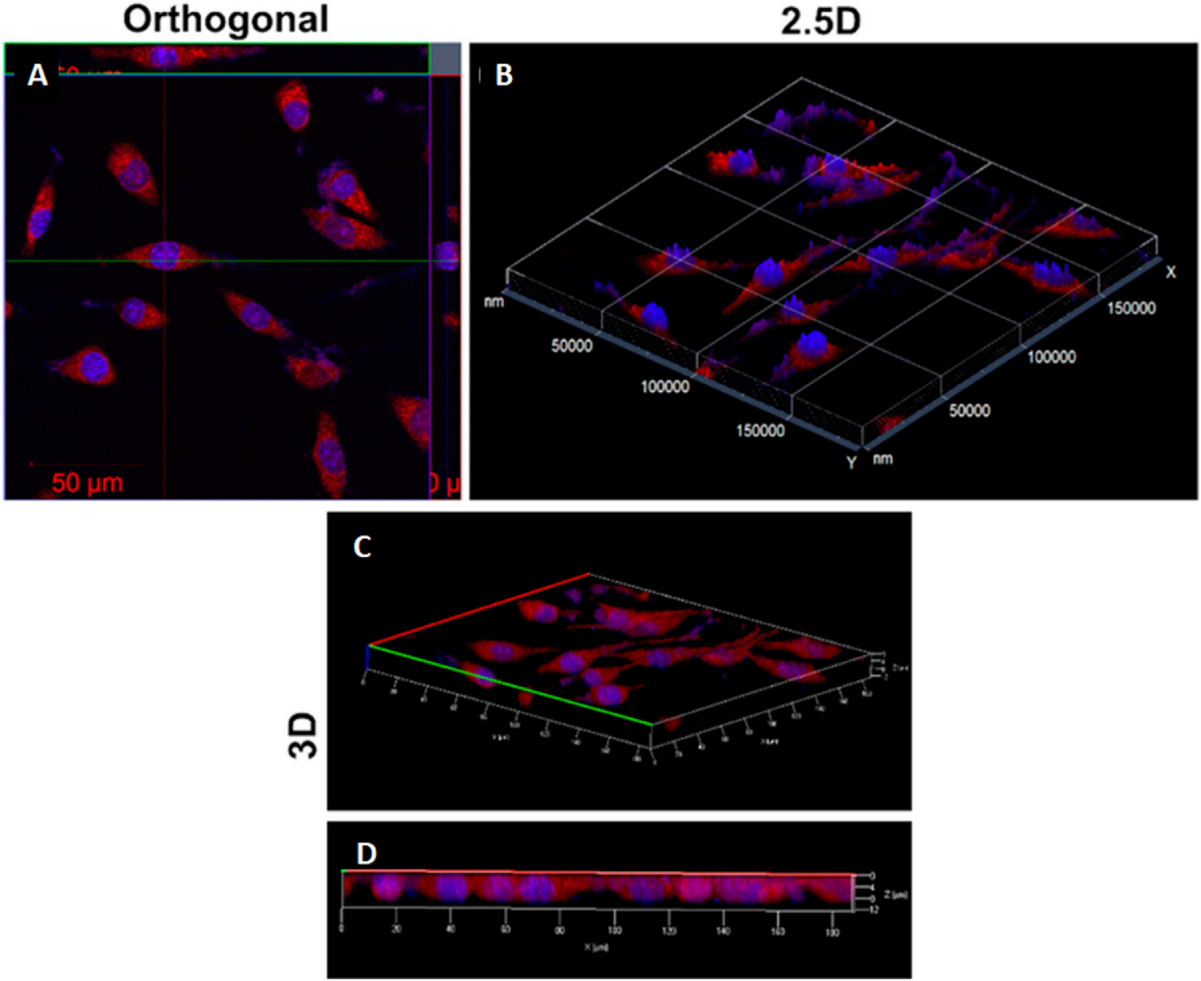
Intracellular localization of mGQDs in L929 cells stained with DAPI and mGQDs. **(A)** The optical section with *x-*, *y-*axes and corresponding projections of *x-*, *z-* and *y-*, *z-*axes of L929 cells; **(B)** 2.5D imaging; and **(C, D)** 3D intracellular imaging showing an obvious localization of signals from nuclei stained with DAPI and cytoplasm stained with mGQDs. Reprinted with permission from Kumawat et al. (2017). Graphene quantum dots from *Mangifera indica*: Application in near-infrared bioimaging and intracellular nanothermometry. *ACS Sustain. Chem. Eng.* 5 (2), 1,382–1,391. doi:10.1021/acssuschemeng.6b01893. Copyright^©^ 2016 American Chemical Society.

A dose dependent cytotoxicity of AAPCDs synthesized from *A. paniculate* was noted against MCF-7 cells where 50% cells were viable at a 2 mg/mL concentration of AAPCDs (Naik et al., 2020). Mewada et al. (2013) showed no killing of MDCL cells by CDs from a *T. bispinosa* peel at 1 μg/mL. However, treatment with 4 μg/mL of CDs resulted in 80.32% alive cells which indicated the exceptional biocompatibility of CDs for novel biological applications. Vandarkuzhali et al. (2017) showed low cytotoxicity of the CDs from a pseudo-stem of the banana plant against HeLa and MCF-7 cells where 95% and 85% live cells were seen upon treatment with 0.5 mg/mL and 1 mg/mL of CDs. Meena et al. (2019) reported 80%–85% of NIH-3T3 cell viability upon treatment with CQDs while it showed no significant effect on the normal cells. Its potential in photothermal therapy was rationalized by a reduction in HeLa cell viability from 98%–100% to 45%–59% when treated with CQDs under the exposure of 750 nm of light. Similarly, the viability of the CQDs treated bacteria decreased from 95% to 80% under the exposure of light. Kumawat et al. (2017) checked the effect of mGQDs synthesized from mango leaves on the cycle of L929 cells where the G2-phase was enhanced from 21.6% to 45.4% indicating increased cell proliferation. However, the QDs did not affect the G0-phase of the cell cycle indicating its biocompatibility. More than 95% of L929 cells were viable even after treatment with 5 μg/mL to 0.1 mg/mL of mGQDs for 24 h. The intercellular localization of the mGQDs can be seen in Figure 7.

### 5.7 Bioimaging

The optical properties of fluorescence QDs are widely exploited for bioimaging applications. Li et al. (2019a) used N/S-CDs derived from *D. salina* to treat *Thalassiosira weisslflogii* and *D. salina* cells. These cells were incubated with 200 μg/mL particles for one growth cycle which showed 100% viability of both cell types. At higher concentrations of particles (400 μg/mL), the viability of cells was around 90.6% (*T. weisslflogii*) and 92.4% (*D. salina*). The concentration of the probe utilized for bioimaging was only 150 μg/mL. Guo et al. (2017) incubated the MCF-7 cells with CQDs fabricated using microalgal powder for 3 h at 37°C. Upon excitation with 800 nm under a fluorescence microscope, the cells showed the presence of CQDs in the cell membrane as well as in the nucleus of the MCF-7 cells which suggested their high permeability into living cells. In a similar study, Ramanan et al. (2016) used CDs fabricated from a eutrophic algal bloom to treat MCF-7 cells at a 0.2 mg/L concentration. The cells showed intense green colored luminescence on their cell membrane as well as in the cytoplasmic areas whereas no luminescence was noted in the cell’s nucleus.

**FIGURE 8:**
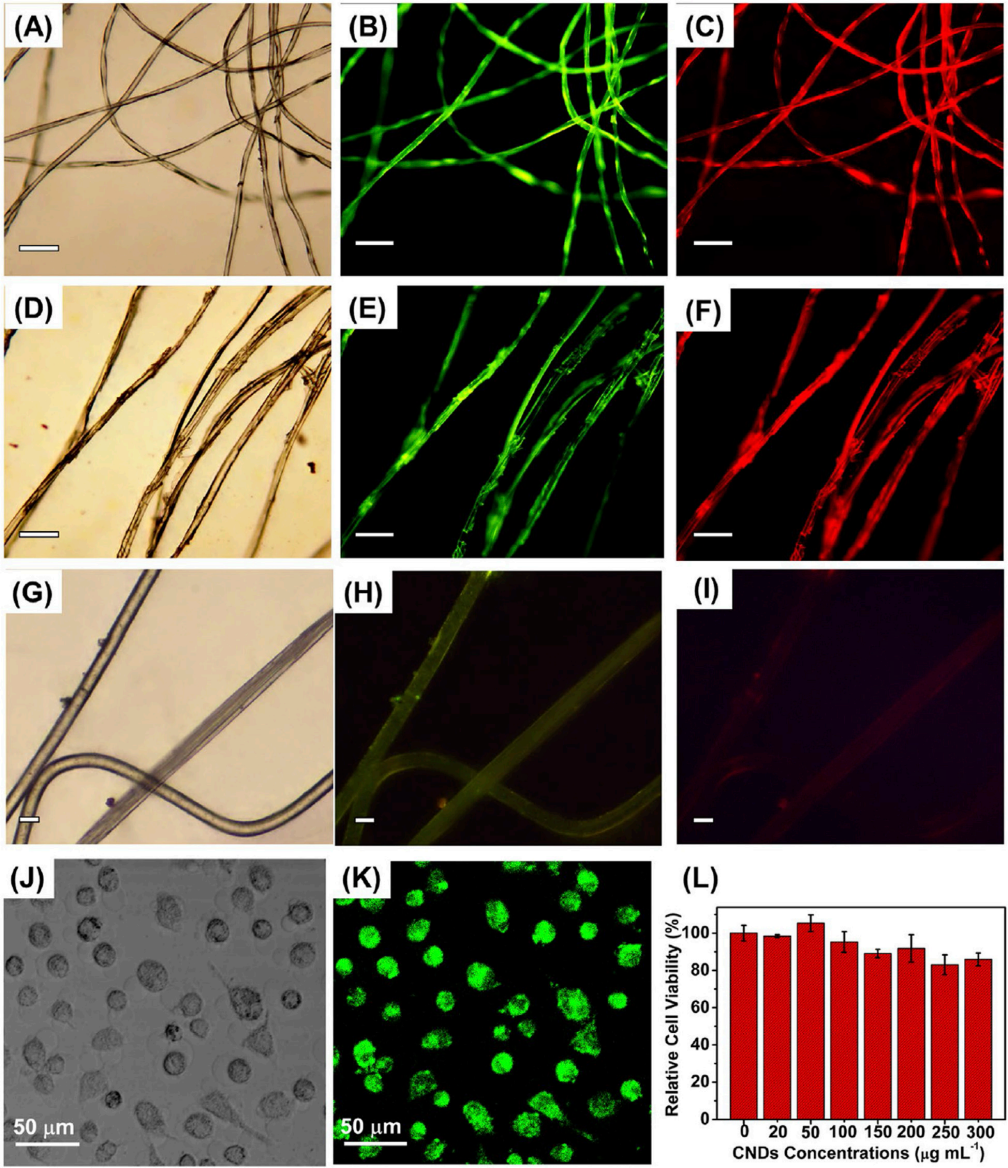
**(A)** Bright field and **(B, C)** fluorescence images of cotton fiber stained with the prepared CNDs. **(D)** Bright-field and **(E, F)** fluorescence images of silk fiber stained with the prepared CNDs. **(G)** Bright field and **(H, I)** fluorescence images of cotton fiber without being stained with the prepared CNDs. The fluorescent images were obtained at the excitation wavelengths of **(B, E)** 450–480 nm, and **(C, F)** 510–550 nm. Scale bars of A–I: 100 μm. LSCM images of HeLa cells incubated with 100 μg mL^−1^ CNDs for 24 h at 37°C: **(J)** bright field images, **(K)** fluorescence images at an excitation wavelength of 488 nm. **(L)** Cell viability of HeLa cells (n = 3, mean ± S.D.) after being incubated with CNDs at various concentrations (0, 20, 50, 100, 150, 200, 250, 300 μg mL^−1^, respectively). Reprinted from Xu et al. (2017). Enhanced-quantum yield sulfur/nitrogen co-doped fluorescent carbon nanodots produced from biomass *Enteromorpha prolifera*: Synthesis, posttreatment, applications and mechanism study. *Sci. Rep.* 7, 4,499. doi:10.1038/s41598-017–04754-x. (Open access).

In another interesting study, Zhang et al. (2017) used the N/S-CDs derived from the *Nannochloropsis* biomass for the imaging of *Arabidopsis thaliana* guard and root cells. Upon treating the guard cells with the particles, an intense blue-colored luminescence was noted in the open as well as closed stomata which suggested their successful penetration in the guard cells. Similarly, the root cells exhibited blue luminescence after treating with the particles and exposing them to 405 nm of excitation wavelength. No damage was noted to the plant tissue cells after treatment with N/S-CDs which suggested biocompatibility of particles as well as lower cytotoxicity. Singh et al. (2019) used 50 μg/mL of CDs fabricated from *D. salina* for treating HEK-293 cells for 24 h. The intracellular uptake of CDs was observed in the dichromic channel (red and green) which was further quenched upon treatment with 100 µM Hg (II) and Cr (VI) solutions. HeLa cells treated with 100 μg/mL of CNDs fabricated using *E. prolifera* emitted bright green fluorescence after excitation with 488 nm intensity of light as evident from Figure 8 (Xu et al., 2017).

Sharma et al. (2022) used CQDs derived from the leaf extract of *C. gigantea* as an alternative suitable fluorescent probe for the optical and bio-imaging of bacteria, fungi, and plant cells. Roy et al. (2015) studied the GQDs synthesized from the neem leaf extract for cell imaging using healthy cells (MCF-10A) and cancer cells (HeLa and MCF-7). Upon excitation with an argon laser light (488 nm), fluorescent green and red colored images of cells were observed. Along with this, the average cell viability of the GQDs treated cells was found to be greater than 95% at a 2 mg/mL concentration. Likewise, Meena et al. (2019) used CQDs for treating the HeLa cells whereas the intracellular localization of CQDs was confirmed by the significantly distributed green fluorescence in the cell interior. Pete et al. (2023) utilized CDs fabricated from *A. squamosa* peel in the germination of mung bean (*Vigna radiata*) seeds. The seedlings were allowed to grow for 8 days. The root length of the seedling was maximum when treated with 4 mg/mL of CDs while 16 mg/mL of the same gave the maximum shoot length. The assimilation of CDs in the plant cells was evaluated by the detection of PL in the germinating mung bean seeds under the UV light (320/312 nm UV-B). The growing parts of the CD treated seeds showed a blue-green fluorescence which confirmed the absorption of CDs without any functionalization.

Bhamore et al. (2019) utilized multicolored emissive CDs synthesized from *M. zapota* fruits for the imaging of different microbes like *E. coli*, *Aspergillus aculeatus*, and *Fomitopsis* sp. CDs were localized in the cell membrane and cytoplasm of these microbes after successful internalization via the endocytosis process. The cells treated with the CDs showed different fluorescence like blue, green and yellow while, no fluorescence was emitted from the untreated cells. Thus, the CDs were internalized easily into the cell due to their ultra-small size which further resulted in their better distribution inside the cell showing bright fluorescent signals during imaging. Shen et al. (2017) used fluorescent CDs synthesized from sweet potatoes in HeLa and HepG2 cell imaging. The CDs, due to their small sizes and hydrophilic functional groups, easily penetrated into these cells. The treated cells emitted blue, green, and red fluorescence upon excitation with ultraviolet, blue, and green light. Thus, the CDs were therefore considered biocompatible and suitable probes for cell imaging. Vandarkuzhali et al. (2017) developed fluorescent CDs from banana pseudo-stem that displayed resistance to high ionic strength, higher fluorescence emission under physiological pH, and good photostability making them a potential candidate for biosensing and bioimaging.

### 5.8 Others

Apart from the above-mentioned applications, biogenic QDs can also be applied for a variety of other purposes, although there are less reports in such areas. Al-Ghamdi et al. (2023) used CQDs fabricated using *H. opuntia* algae to develop CQDs/GCE which was further electrochemically analyzed for storage applications. The constant current density of 1 A/g was observed for different charge-discharge cycles (10–200 cycles). The highest specific capacitance (C_sp_) of 311 F/g was noted at a 1 A/g current density. These results suggest its higher priority for energy storage applications.

Ana and Camacho (2019) evaluated the application of CDs fabricated from cellulose extracted from *C. rupestris* as the carbon nanodots sensitized solar cells (CND-SSC). Under dark conditions, a diode property was observed in the solar cell which demonstrated no flow of current in dark conditions. However, under the illumination of light, a shift in the current-voltage curve was noted along with the generation of current. Power conversion efficiencies of 0.00362% and 0.0176% were noted for CDs derived from RC and CNC precursors, respectively.

Shivaji et al. (2018) reported 1.83% ± 0.20% hemolysis upon treatment with 60 μg/mL CdSQDs from the tea leaf extract. Suresh et al. (2023) investigated the use of CD-Ag NCs derived from *C. hornemanni* against the larvae of *Aedes aegypti*. The lethal concentration (LC_50_) of NCs in the elimination of 50% of the 3^rd^ and 4^th^ instar larva ranged from 27.595 μg/mL to 39.180 μg/mL. Similarly, the LC_90_ value was in the range from 109.656 to 119.780 μg/mL for 3^rd^ and 4^th^ instar larvae. The egg yielding capacity was 161.6, 131, 114.3, 75, and 51.3 when the mosquitos were treated with 5, 10, 20, 40, and 80 μg/mL of NCs, respectively.

Guo et al. (2021) used CDs fabricated from *C. pyrenoidosa* as a fluorescent ink. Around 20 mg CDs were added into 4 mL water followed by the addition of 1 mL glycerine which acted as the thickener. This solution was further used in the preparation of fluorescent ink stamps. Li et al. (2019b) studied the phytophysiological effect of SiQDs derived from *T. rotula* on the cucumber seedling. A 51.91% enhancement in its fresh weight was observed after 10 days of incubation with 0.05 mg/mL of SiQDs. The water uptake rate by the roots increased by 74.6% after 5 days of incubation. About 16.3 times the increase in the concentration of Si was noted in the roots after treatment with 0.3 mg/mL SiQDs. The expression of aquaporins such as CsPIP1-2, CsPIP2-1, CsPIP2-4, and CsPIP2-5 increased by 116.3, 155.7, 43.7, 25.3%, respectively, after 5 days of incubation while the same was 177, 106.3, 113.3, and 116.7%, respectively, after 10 days of incubation. Also, Chatzimitakos et al. (2020) evaluated the use of CNDs derived from *D. salina* as sun protection filters. The CNDs (11 mg/mL) exhibiting absorption peaks between 290 and 320 nm were used in the preparation of the highest sun protection filter of 37.

## 6 Future prospects

The growing demands of QDs in electronic appliances and nanomedicine emphasizes the need to develop more rapid, efficient and environmentally benign synthesis strategies for the same. Biocompatibility and stability are the two most important aspects that functionality of QDs can achieve. Although, preliminary reports suggests that photoautotrophs have promising nanobiotechnological potential for QDs synthesis, there is a wide scope in this area where cutting edge research can be done. Biogenic QDs can be doped with suitable dopants as they can auto-ionize due to quantum confinement unlike pure QDs that often require thermal activation (Robkhob et al., 2020).

Rational doping with Ag, Cr, Co, Fe, Na, or Li may enhance the properties of QDs due to autoionization when the quantum confinement energy exceeds *Coulombic* interaction between carrier (hole or electron) and impurity (*n*-type or *p*-type). Similarly, alloying the core of QDs can help in engineering the band-gap of the biogenic QDs that can modify its optoelectronic properties (Bloch et al., 2022; Farzin and Abdoos, 2021). Introduction of surface defects in the biogenic QDs can serve as temporary ‘traps’ for the electrons, holes or excitons, quenching free radicals resulting in enhanced antioxidant activity. This can help in developing efficient nanomedicine that may prevent generation of reactive oxygen species (ROS) mediated oxidative stress (Asok et al., 2015).

Biogenic synthesis of CQDs is essentially a ‘Bottom-Up’ approach that can be well understood employing first principles modelling (Cabral et al., 2016). Simulation studies revealed that initially a layered system is formed with limited amounts of C-C bonds between the members of the adjacent layers (Boukhvalov and Osipov, 2023). Eventually, oxidation along the edges as well as in the basal plane associated with the growing surface results in the graphenic systems. However, after reaching a size equivalent to ∼1 nm, the lateral growth of these molecular structures seizes. This is mainly attributed to a large amount of structural defects introduced at the peripheral region of the quasi-graphene planes owing to their saturation with oxygen-containing groups. This results in the formation of the nucleation centers that are composed of a countable number of atoms that serve as seeds for further growth of the graphene nanostructure which is often 5–7 nm. This structural evolution is accompanied by topological synchronization and coherence of crystal lattices between the adjacent layers assigned to the AA stacking type.

Politi et al. (2021) used the first principles approach for studying the interaction between CQDs and alkali metals. The C–C bond distances were between 1.25 Å to 1.45 Å, while the carbon atoms were arranged in a triangular pattern at the CQD_32_ lower edges. In another significant study, the state-of-the-art first-principles calculation was used for studying the intercalation energetics when various alkali metals like Li, Na, K, Rb, and Cs were doped in a bi-layer graphene and MoS_2_. The formation energy of the multi-layer structures was negative when K, Rb, and Cs were doped unlike Li, and Na that were slightly positive (Chepkasov et al., 2020; Heath and Kuroda, 2018). Although first principles modelling is used for studying chemically and physically synthesized carbon based QDs, it would be interesting to extend this model to biogenic QDs as well. This would provide an in-depth understanding about the intrinsic properties of the QDs and mechanism behind their synthesis.

Doped CQDs have tremendous potential as theranostic agents which can simultaneously monitor drug targeting, triggered release, cellular uptake, and biodistribution in tissues. In a recent study, bi-doped CQDs were integrated with liposomes for fluorescence visualization and cancer control (Zhu et al., 2024). This biomaterial termed as lipo/Bi-doped CQDs was used for labelling of colon cancer (CT26) cells.

Thus, there is a scope to integrate the biogenic QDs with liposomes in a similar fashion for *in vivo* antitumor therapy. Further, multi-element charge-transfer co-doped CDs can be fabricated using a phycogenic and/or phytogenic route that can serve as an enhanced dual-mode imaging contrast agent for anticancer applications. This is rationalized by a recent study, where elements such as P, W, and Hf were co-doped in CDs that exhibited high long-wavelength quantum yields up to 27.3% (Zhu et al., 2023). Interestingly, these novel nanostructures were biocompatible which were employed for 143B cell imaging, and fluorescence/CT high contrast imaging performance in Balb/C tumor-bearing mice.

Enhancement QY of CDs is an important aspect that has a deep impact on its functionality, especially in the field of fluorescent probes, LEDs, and photocatalysis. Among various strategies that aim to increase the QY, doping of diverse non-metallic heteroatoms, such as S and N are considered most significant. It is important to note that single atom doping of CDs with S and N can result in 67% and 80% enhancement, respectively, in the QY. Also, P and B doping either individually or co-doping with S and N can synergistically enhanced the QY of CDs.

Additionally, doping with metals like Ba, Cu, Gd, Mn, and Zn facilitate the radiative recombination of electrons and hole on the surface of CD. The valence electrons in the metallic dopant atoms or metal carbonite promote the charge transfer, eventually resulting in a QY enhancement equivalent to 30% or more (Xu et al., 2018b). Controlled graphitization, surface functionalization, and selection of appropriate precursors like dopamine and/or o-phenylenediamine can also enhance the QY of the QDs (Liu et al., 2021). The aforementioned strategies can be applied to fabricate biogenic QDs with higher QY.

Cellular metabolites may help in the surface passivation during biogenesis of QDs making them photostable. This will saturate all dangling bonds, exhibiting no surface state. Additionally, this will led to internal quantum-confinement of all near band-edge states in the biogenic QDs. Such organic capping will also keep the QDs monodispersed as a colloidal suspension (Kitture et al., 2015). This is advantageous for bioconjugating the QDs with an appropriate ligand ideal for bioimaging and targeted therapy. Although, in general, mercaptans (-SH) and/or phosphenes are mostly used ligands, and they can be replaced with enzymes, antibodies and/or aptamers depending on the application. QDs with an inverted core/shell can be fabricated biologically that may exhibit either type I or type II interfacial band offsets which is a function of the core radius and the shell thickness. The reaction parameters like time, temperature, pH, extract and precursor concentrations can be carefully optimized so that the lattice parameter of the shell material is within 12% of the core to promote epitaxy and thereby provide better passivation (Uprety and Abrahamse, 2022).

During physical and chemical synthesis, the QDs are often fabricated in nonpolar solvents rendering them hydrophobic (Karmakar et al., 2020). This makes them unfavorable for biological applications. Hence, coating them with polar biomolecules or oxide-based materials will not only decrease their toxicity, but also enhance their stability in physiological conditions. Tailor-made biofabrication of multi-shell QDs will reduce the interfacial lattice strain while maintaining the large band offsets (Bera et al., 2010). In view of the background, there is a tremendous scope to explore the untapped possibilities in the area of biogenic QDs from the photoautotrophs.

## 7 Conclusion

Based on the green synthesis strategies, this review is the first of its kind which aims to cover one of the most untouched areas of nanobiotechnology where photoautotrophs are exclusively explored for QDs synthesis. Significant synthesis approaches and figures of merit of these biogenic QDs are highlighted in this study. In-depth mechanisms behind the synthesis, biomolecules involved and the metabolic pathways are discussed in detail. Despite their notable progress in the field of nanotechnology, certain challenges still exist that include tunable optical properties and stability of the biogenic nanoparticles. These limitations have greatly affected the acceptability and introduction of the same in commercial products like sensors, solar cells and therapeutics. Therefore, biogenic nanoparticles with well-defined physicochemical and optoelectronic properties are in high-demand for industrial applications which should be affordable and effective.

This review emphasizes QDs as the newest generation of photostable biogenic nanoparticles that can generate singlet oxygen by triplet energy transfer (TET) and enhance the photoluminescence of classical photosensitizers via FRET. The synthesis route is rapid, efficient and nontoxic while the resulting QDs stabilized by cellular metabolites associated amines, carboxylic acids, alcohols, and thiols form stable aqueous dispersion. The biogenic QDs can be functionalized with specific ligands for the site specific localization of the photosensitizers avoiding undesired phototoxicity. Thus, biogenic QDs can serve as next-generation nanotheranostics due to their promising potential in photothermal and photodynamic therapy as well as bioimaging. However, currently, no reports are available on the pharmacokinetics and pharmacodynamics of QDs synthesized from photoautotrophs. Hence, a thorough investigation of biogenic QDs for long term toxicity to human health and the environment would further provide a strong rationale for their use in healthcare and environmental remediation.

## References

[B1] Ahmadian-Fard-Fini S. Ghanbari D. Amiri O. Salavati-Niasari M. (2021). Green sonochemistry assisted synthesis of hollow magnetic and photoluminescent MgFe_2_O_4_–carbon dot nanocomposite as a sensor for toxic Ni (ii), Cd (ii) and Hg (ii) ions and bacteria. RSC Adv. 11 (37), 22805–22811. 10.1039/d1ra02458b 35480469 PMC9034268

[B2] Al-Ghamdi S. A. Darwish A. A. A. Hamdalla T. A. Pasha A. Elnair M. E. Al-Atawi A. (2023). Biological synthesis of novel carbon quantum dots using *Halimeda opuntia* green algae with improved optical properties and electrochemical performance for possible energy storage applications. Int. J. Electrochem. Sci. 18 (5), 100102. 10.1016/j.ijoes.2023.100102

[B3] Alvand Z. M. Rajabi H. R. Mirzaei A. Masoumiasl A. Sadatfaraji H. (2019). Rapid and green synthesis of cadmium telluride quantum dots with low toxicity based on a plant-mediated approach after microwave and ultrasonic assisted extraction: synthesis, characterization, biological potentials and comparison study. Mater. Sci. Eng. C 98, 535–544. 10.1016/j.msec.2019.01.010 30813055

[B4] Amjad M. Iqbal M. Faisal A. Junjua A. M. Hussain I. Hussain S. Z. (2019). Hydrothermal synthesis of carbon nanodots from bovine gelatin and PHM3 microalgae strain for anticancer and bioimaging applications. Nanoscale Adv. 1, 2924–2936. 10.1039/c9na00164f 36133618 PMC9419553

[B5] Ana J. C. S. Camacho D. H. (2019). Influence of precursor size in the hydrothermal synthesis of cellulose-based carbon nanodots and its application towards solar cell sensitization. Mater. Chem. Phys. 228, 187–193. 10.1016/j.matchemphys.2019.02.073

[B6] Anooj E. S. Praseetha P. K. (2019). Synthesis and characterization of graphene quantum dots from nutmeg seeds and its biomedical application. Int. J. Recent Technol. Eng. 7 (6S5), 144–151 .Retrieval Number: F10220476S519/19 ©BEIESP.

[B7] Arumugam N. Kim J. (2018). Synthesis of carbon quantum dots from Broccoli and their ability to detect silver ions. Mater. Lett. 219, 37–40. 10.1016/j.matlet.2018.02.043

[B8] Arumugham T. Alagumuthu M. Amimodu R. G. Munusamy S. Iyer S. K. (2020). A sustainable synthesis of green carbon quantum dot (CQD) from *Catharanthus roseus* (white flowering plant) leaves and investigation of its dual fluorescence responsive behavior in multi-ion detection and biological applications. Sustain. Mater. Technol. 23, e00138. 10.1016/j.susmat.2019.e00138

[B9] Asok A. Ghosh S. More P. A. Chopade B. A. Gandhi M. N. Kulkarni A. R. (2015). Surface defect rich ZnO quantum dots as antioxidants inhibiting α-amylase and α-glucosidase: a potential anti-diabetic nanomedicine. J. Mater. Chem. B 3 (22), 4597–4606. 10.1039/c5tb00407a 32262403

[B10] Bentolila L. A. (2015). “Photoluminescent quantum dots in imaging, diagnostics and therapy,” in Applications of Nanoscience in Photomedicine. Editors Hamblin, M. R. Avci P. (UK: Chandos Publishing, Elsevier Inc.), 77–104.

[B11] Bera D. Qian L. Tseng T.-K. Holloway P. H. (2010). Quantum dots and their multimodal applications: a review. Materials 3, 2260–2345. 10.3390/ma3042260

[B12] Bhamore J. R. Jha S. Park T. J. Kailasa S. K. (2019). Green synthesis of multi-color emissive carbon dots from *Manilkara zapota* fruits for bioimaging of bacterial and fungal cells. J. Photochem. Photobiol. B 191, 150–155. 10.1016/j.jphotobiol.2018.12.023 30639997

[B13] Bloch K. Mohammed S. M. Karmakar S. Shukla S. Asok A. Banerjee K. (2022). Catalytic dye degradation by novel phytofabricated silver/zinc oxide composites. Front. Chem. 10, 1013077. 10.3389/fchem.2022.1013077 36385994 PMC9659756

[B14] Bloch K. Sarkar B. Ghosh S. (2024). Microbial fabrication of quantum dots: mechanism and applications. Curr. Microbiol. 81, 294. 10.1007/s00284-024-03813-7 39095512

[B15] Boukhvalov D. W. Osipov V. Y. (2023). First-principles modeling of bottom-up synthesis of carbon quantum dots. Crystals 13 (5), 716. 10.3390/cryst13050716

[B16] Cabral B. J. C. Coutinho K. Canuto S. (2016). A first-principles approach to the dynamics and electronic properties of p-nitroaniline in water. J. Phys. Chem. A 120 (22), 3878–3887. 10.1021/acs.jpca.6b01797 27187208

[B17] Calangian M. T. F. Ildefonzo A. B. Manzano V. K. S. Agcaoili G. J. T. Ganado R. J. J. Yago A. C. C. (2018). Facile synthesis of biologically derived fluorescent carbon nanoparticles (FCNPs) from an abundant marine alga and its biological activities. Orient. J. Chem. 34 (2), 791–799. 10.13005/ojc/340224

[B18] Centeno L. Romero-García J. Alvarado-Canché C. Gallardo-Vega C. Télles-Padilla G. Barriga-Castro E. D. (2021). Green synthesis of graphene quantum dots from *Opuntia* sp. extract and their application in phytic acid detection. Sens. Bio-Sens. Res. 32, 100412. 10.1016/j.sbsr.2021.100412

[B19] Chatzimitakos T. G. Kasouni A. Troganis A. Leonardos I. Tzovenis I. Ntzouvaras A. (2020). Carbon nanodots synthesized from *Dunaliella salina* as sun protection filters. C 6 (4), 69. 10.3390/c6040069

[B20] Chepkasov I. V. Ghorbani-Asl M. Popov Z. I. Smet J. H. Krasheninnikov A. V. (2020). Alkali metals inside bi-layer graphene and MoS_2_: insights from first-principles calculations. Nano Energy 75, 104927. 10.1016/j.nanoen.2020.104927

[B21] De B. Karak N. (2013). A green and facile approach for the synthesis of water soluble fluorescent carbon dots from banana juice. RSC Adv. 3 (22), 8286–8290. 10.1039/c3ra00088e

[B22] Demchenko A. P. Dekaliuk M. O. (2013). Novel fluorescent carbonic nanomaterials for sensing and imaging. Methods Appl. Fluoresc. 1 (4), 042001. 10.1088/2050-6120/1/4/042001 29148449

[B23] Dong D. Liu T. Liang D. Jin X. Qi Z. Li A. (2021). Facile hydrothermal synthesis of *Chlorella*-derived environmentally friendly fluorescent carbon dots for differentiation of living and dead *Chlorella* . ACS Appl. Bio Mater. 4 (4), 3697–3705. 10.1021/acsabm.1c00178 35014454

[B24] Du F. Zhang M. Li X. Li J. Jiang X. Li Z. (2014). Economical and green synthesis of bagasse-derived fluorescent carbon dots for biomedical applications. Nanotechnol 25 (31), 315702. 10.1088/0957-4484/25/31/315702 25036467

[B25] Farzin M. A. Abdoos H. (2021). A critical review on quantum dots: from synthesis toward applications in electrochemical biosensors for determination of disease-related biomolecules. Talanta 224, 121828. 10.1016/j.talanta.2020.121828 33379046

[B26] Gholami Z. Dadmehr M. Jelodar N. B. Hosseini M. Parizi A. P. Pakdin Parizi A. (2020). One-pot biosynthesis of CdS quantum dots through *in vitro* regeneration of hairy roots of *Rhaphanus sativus L*. and their apoptosis effect on MCF-7 and AGS cancerous human cell lines. Mater. Res. Express 7 (1), 015056. 10.1088/2053-1591/ab66ea

[B27] Gong X. Gao X. Du W. Zhang H. Zhang S. Nguyen T. T. (2019). Wood powder-derived quantum dots for CeO_2_ photocatalytic and anti-counterfeit applications. Opt. Mater. 96, 109302. 10.1016/j.optmat.2019.109302

[B28] Guo C. X. Xie J. Wang B. Zheng X. Yang H. B. Li C. M. (2013). A new class of fluorescent-dots: long luminescent lifetime bio-dots self-assembled from DNA at low temperatures. Sci. Rep. 3, 2957. 10.1038/srep02957 24129792 PMC3797422

[B29] Guo D. Lyu Y. Gao Y. Lin Y. Zhang X. Pan Y. (2021). Synthesis, solution and solid-state fluorescence of nitrogen self-doped carbon dots derived from *Chlorella pyrenoidosa* . Colloids Surf. A Physicochem. Eng. Asp. 631, 127741. 10.1016/j.colsurfa.2021.127741

[B30] Guo L. P. Zhang Y. Li W. C. (2017). Sustainable microalgae for the simultaneous synthesis of carbon quantum dots for cellular imaging and porous carbon for CO_2_ capture. J. Colloid Interface Sci. 493, 257–264. 10.1016/j.jcis.2017.01.003 28110060

[B31] Gusain D. Renuka N. Guldhe A. Bux F. (2021). Use of microalgal lipids and carbohydrates for the synthesis of carbon dots via hydrothermal microwave treatment. Inorg. Chem. Commun. 134, 109021. 10.1016/j.inoche.2021.109021

[B32] Heath J. J. Kuroda M. A. (2018). First principles studies of the interactions between alkali metal elements and oxygen-passivated nanopores in graphene. Phys. Chem. Chem. Phys. 20 (40), 25822–25828. 10.1039/C8CP04958K 30283971

[B33] Hoan B. T. Tam P. D. Pham V. H. (2019). Green synthesis of highly luminescent carbon quantum dots from lemon juice. J. Nanotechnol. 2019 (1), 1–9. 10.1155/2019/2852816

[B34] Huang G. Chen X. Wang C. Zheng H. Huang Z. Chen D. (2017). Photoluminescent carbon dots derived from sugarcane molasses: synthesis, properties, and applications. RSC Adv. 7 (75), 47840–47847. 10.1039/c7ra09002a

[B35] Huang H. Li C. Zhu S. Wang H. Chen C. Wang Z. (2014). Histidine-derived nontoxic nitrogen-doped carbon dots for sensing and bioimaging applications. Langmuir 30 (45), 13542–13548. 10.1021/la503969z 25375765

[B36] Jain A. Wadhawan S. Kumar V. Mehta S. K. (2018). Colorimetric sensing of Fe^3+^ ions in aqueous solution using magnesium oxide nanoparticles synthesized using green approach. Chem. Phys. Lett. 706, 53–61. 10.1016/j.cplett.2018.05.069

[B37] Jiang J. He Y. Li S. Cui H. (2012). Amino acids as the source for producing carbon nanodots: microwave assisted one-step synthesis, intrinsic photoluminescence property and intense chemiluminescence enhancement. Chem. Commun. 48 (77), 9634–9636. 10.1039/c2cc34612e 22908119

[B38] Jin H. Livache C. Kim W. D. Diroll B. T. Schaller R. D. Klimov V. I. (2023). Spin-exchange carrier multiplication in manganese-doped colloidal quantum dots. Nat. Mater. 22 (8), 1013–1021. 10.1038/s41563-023-01598-x 37443379 PMC10390332

[B39] Jones S. S. Sahatiya P. Badhulika S. (2017). One step, high yield synthesis of amphiphilic carbon quantum dots derived from chia seeds: a solvatochromic study. New J. Chem. 41 (21), 13130–13139. 10.1039/c7nj03513f

[B40] Karmakar S. Ghosh S. Kumbhakar P. (2020). Enhanced sunlight-driven photocatalytic and antibacterial activities of flower-like ZnO@MoS_2_ nanocomposite. J. Nanopart. Res. 22 (11), 11. 10.1007/s11051-019-4710-3

[B41] Khose R. V. Chakraborty G. Bondarde M. P. Wadekar P. H. Ray A. K. Some S. (2021). Red-fluorescent graphene quantum dots from guava leaf as a turn-off probe for sensing aqueous Hg (ii). New J. Chem. 45 (10), 4617–4625. 10.1039/d0nj06259f

[B42] Kim K. W. Chung D. Jung S. H. Kwon Y. M. Kim J. Y. H. Baek K. (2022b). Antimicrobial effect of carbon nanodots–ZnO nanocomposite synthesized using *Sargassum horneri* . J. Mar. Sci. Eng. 10 (10), 1546. 10.3390/jmse10101546

[B43] Kim K. W. Kwon Y. M. Kim S. Y. Kim J. Y. H. (2022a). One-pot synthesis of UV-protective carbon nanodots from sea cauliflower (*Leathesia difformis*). Electron. J. Biotechnol. 56, 22–30. 10.1016/j.ejbt.2021.12.004

[B44] Kitture R. Chordiya K. Gaware S. Ghosh S. More P. A. Kulkarni P. (2015). ZnO nanoparticles-red sandalwood conjugate: a promising anti-diabetic agent. J. Nanosci. Nanotechnol. 15 (6), 4046–4051. 10.1166/jnn.2015.10323 26369011

[B45] Kumar N. Kumar R. (2013). Nanotechnology and nanomaterials in the treatment of Life-threatening Diseases. United States: Elsevier Inc.

[B46] Kumawat M. K. Thakur M. Gurung R. B. Srivastava R. (2017). Graphene quantum dots from *Mangifera indica*: application in near-infrared bioimaging and intracellular nanothermometry. ACS Sustain. Chem. Eng. 5 (2), 1382–1391. 10.1021/acssuschemeng.6b01893

[B47] Li Q. H. Zhang L. Bai J. M. Liu Z. C. Liang R. P. Qiu J. D. (2015). Preparation of novel fluorescent DNA bio-dots and their application for biothiols and glutathione reductase activity detection. Biosens. Bioelectron. 74, 886–894. 10.1016/j.bios.2015.07.018 26248043

[B48] Li Y. Li W. Zhang H. Dong R. Li D. Liu Y. (2019b). Biomimetic preparation of silicon quantum dots and their phytophysiology effect on cucumber seedlings. J. Mater. Chem. B. 7 (7), 1107–1115. 10.1039/C8TB02981D 32254778

[B49] Li Y. Liu F. Cai J. Huang X. Lin L. Lin Y. (2019a). Nitrogen and sulfur co-doped carbon dots synthesis via one step hydrothermal carbonization of green alga and their multifunctional applications. Microchem. J. 147, 1038–1047. 10.1016/j.microc.2019.04.015

[B50] Liu Y. Liu Y. Park S. J. Zhang Y. Kim T. Chae S. (2015). One-step synthesis of robust nitrogen-doped carbon dots: acid-evoked fluorescence enhancement and their application in Fe^3+^ detection. J. Mater. Chem. A 3 (34), 17747–17754. 10.1039/c5ta05189d

[B51] Liu Y. Wei J. Yan X. Zhao M. Guo C. Xu Q. (2021). Barium charge transferred doped carbon dots with ultra-high quantum yield photoluminescence of 99.6% and applications. Chin. Chem. Lett. 32 (2), 861–865. 10.1016/j.cclet.2020.05.037

[B52] Liu Y. Zhou Q. Yuan Y. Wu Y. (2017). Hydrothermal synthesis of fluorescent carbon dots from sodium citrate and polyacrylamide and their highly selective detection of lead and pyrophosphate. Carbon 115, 550–560. 10.1016/j.carbon.2017.01.035

[B53] Malavika J. P. Shobana C. Ragupathi M. Kumar P. Lee Y. S. Govarthanan M. (2021). A sustainable green synthesis of functionalized biocompatible carbon quantum dots from *Aloe barbadensis* Miller and its multifunctional applications. Environ. Res. 200, 111414. 10.1016/j.envres.2021.111414 34052245

[B54] Mangalampalli B. Dumala N. Grover P. (2018). *Allium cepa* root tip assay in assessment of toxicity of magnesium oxide nanoparticles and microparticles. J. Environ. Sci. 66, 125–137. 10.1016/j.jes.2017.05.012 29628079

[B55] Meena R. Singh R. Marappan G. Kushwaha G. Gupta N. Meena R. (2019). Fluorescent carbon dots driven from ayurvedic medicinal plants for cancer cell imaging and phototherapy. Heliyon 5 (9), E02483. 10.1016/j.heliyon.2019.e02483 31687577 PMC6819859

[B56] Mewada A. Pandey S. Shinde S. Mishra N. Oza G. Thakur M. (2013). Green synthesis of biocompatible carbon dots using aqueous extract of *Trapa bispinosa* peel. Mater. Sci. Eng. C 33 (5), 2914–2917. 10.1016/j.msec.2013.03.018 23623114

[B57] Mondal J. Srivastava S. K. (2018). Green synthesis of carbon dot weak gel from pear juice: optical properties and sensing application. ChemistrySelect 3 (29), 8444–8457. 10.1002/slct.201801383

[B58] Mukherjee A. Sarkar D. Sasmal S. (2021). A review of green synthesis of metal nanoparticles using algae. Front. Microbiol. 12, 693899. 10.3389/fmicb.2021.693899 34512571 PMC8427820

[B59] Naik G. G. Alam M. B. Pandey V. Mohapatra D. Dubey P. K. Parmar A. S. (2020). Multi-functional carbon dots from an ayurvedic medicinal plant for cancer cell bioimaging applications. J. Fluoresc. 30, 407–418. 10.1007/s10895-020-02515-0 32088852

[B60] Nasseri M. A. Keshtkar H. Kazemnejadi M. Allahresani A. (2020). Phytochemical properties and antioxidant activity of *Echinops persicus* plant extract: green synthesis of carbon quantum dots from the plant extract. SN Appl. Sci. 2, 670. 10.1007/s42452-020-2466-0

[B61] Peng H. Travas-Sejdic J. (2009). Simple aqueous solution route to luminescent carbogenic dots from carbohydrates. Chem. Mater. 21 (23), 5563–5565. 10.1021/cm901593y

[B62] Pete A. M. Ingle P. U. Raut R. W. Shende S. S. Rai M. Minkina T. M. (2023). Biogenic synthesis of fluorescent carbon dots (CDs) and their application in bioimaging of agricultural crops. Nanomater 13 (1), 209. 10.3390/nano13010209 PMC9824522 36616122

[B63] Pisal V. Wakchaure P. Patil N. Bhagwat S. (2019). Green synthesized CeO_2_ quantum dots: a study of its antimicrobial potential. Mater. Res. Express 6 (11), 115409. 10.1088/2053-1591/ab4fa5

[B64] Placido J. Bustamante-Lopez S. Meissner K. E. Kelly D. E. Kelly S. L. (2019). Microalgae biochar-derived carbon dots and their application in heavy metal sensing in aqueous systems. Sci. Total Environ. 656, 531–539. 10.1016/j.scitotenv.2018.11.393 30529956

[B65] Politi J. R. S. Martins J. B. L. Cabral B. J. C. (2021). A first principles approach to the interactions of alkali metal atoms with carbon quantum dots. Comput. Mater. Sci. 197, 110614. 10.1016/j.commatsci.2021.110614

[B66] Raja D. Sundaramurthy D. (2021). Facile synthesis of fluorescent carbon quantum dots from Betel leafs (Piper betle) for Fe^3+^ sensing. Mater. Today Proc. 34 (2), 488–492. 10.1016/j.matpr.2020.03.096

[B67] Ramanan V. Thiyagarajan S. K. Raji K. Suresh R. Sekar R. Ramamurthy P. (2016). Outright green synthesis of fluorescent carbon dots from eutrophic algal blooms for *in vitro* imaging. ACS Sustain. Chem. Eng. 4 (9), 4724–4731. 10.1021/acssuschemeng.6b00935

[B68] Ramanujam K. Sundrarajan M. (2014). Antibacterial effects of biosynthesized MgO nanoparticles using ethanolic fruit extract of *Emblica officinalis* . J. Photochem. Photobiol. B. 141, 296–300. 10.1016/j.jphotobiol.2014.09.011 25463681

[B69] Robkhob P. Ghosh S. Bellare J. Jamdade D. Tang I. M. Thongmee S. (2020). Effect of silver doping on antidiabetic and antioxidant potential of ZnO nanorods. J. Trace Elem. Med. Biol. 58, 126448. 10.1016/j.jtemb.2019.126448 31901726

[B70] Roy P. Periasamy A. P. Lin C. Y. Her G. M. Chiu W. J. Li C. L. (2015). Photoluminescent graphene quantum dots for *in vivo* imaging of apoptotic cells. Nanoscale 7 (6), 2504–2510. 10.1039/c4nr07005d 25569453

[B71] Sahu S. Behera B. Maiti T. K. Mohapatra S. (2012). Simple one-step synthesis of highly luminescent carbon dots from orange juice: application as excellent bio-imaging agents. Chem. Commun. 48 (70), 8835–8837. 10.1039/C2CC33796G 22836910

[B72] Salem J. K. El-Nahhal I. M. Hammad T. M. Kuhn S. Sharekh S. A. El-Askalani M. (2015). Optical and fluorescence properties of MgO nanoparticles in micellar solution of hydroxyethyl laurdimonium chloride. Chem. Phys. Lett. 636, 26–30. 10.1016/j.cplett.2015.07.014

[B73] Shahshahanipour M. Rezaei B. Ensafi A. A. Etemadifar Z. (2019). An ancient plant for the synthesis of a novel carbon dot and its applications as an antibacterial agent and probe for sensing of an anti-cancer drug. Mater. Sci. Eng. C 98, 826–833. 10.1016/j.msec.2019.01.041 30813088

[B74] Sharma N. Sharma I. Bera M. K. (2022). Microwave-assisted green synthesis of carbon quantum dots derived from *Calotropis gigantea* as a fluorescent probe for bioimaging. J. Fluoresc. 32, 1039–1049. 10.1007/s10895-022-02923-4 35262854

[B75] Shen J. Shang S. Chen X. Wang D. Cai Y. (2017). Facile synthesis of fluorescence carbon dots from sweet potato for Fe^3+^ sensing and cell imaging. Mater. Sci. Eng. C 76, 856–864. 10.1016/j.msec.2017.03.178 28482600

[B76] Shi B. Su Y. Zhang L. Huang M. Liu R. Zhao S. (2016). Nitrogen and phosphorus co-doped carbon nanodots as a novel fluorescent probe for highly sensitive detection of Fe^3+^ in human serum and living cells. ACS Appl. Mater. Interfaces 8 (17), 10717–10725. 10.1021/acsami.6b01325 27014959

[B77] Shivaji K. Mani S. Ponmurugan P. De Castro C. S. Lloyd Davies M. Balasubramanian M. G. (2018). Green-synthesis-derived CdS quantum dots using tea leaf extract: antimicrobial, bioimaging, and therapeutic applications in lung cancer cells. ACS Appl. Nano Mater. 1 (4), 1683–1693. 10.1021/acsanm.8b00147

[B78] Shukla D. Das M. Kasade D. Pandey M. Dubey A. K. Yadav S. K. (2020). Sandalwood-derived carbon quantum dots as bioimaging tools to investigate the toxicological effects of malachite green in model organisms. Chemosphere 248, 125998. 10.1016/j.chemosphere.2020.125998 32006833

[B79] Silva A. Martínez-Gallegos S. Rosano-Ortega G. Schabes-Retchkiman P. Vega-Lebrún C. Albiter V. (2017). Nanotoxicity for *E. coli* and characterization of silver quantum dots produced by biosynthesis with *Eichhornia crassipes* . J. Nanostruct. 7 (1), 1–12. 10.22052/jns.2017.01.001

[B80] Singh A. K. Pal P. Gupta V. Yadav T. P. Gupta V. Singh S. P. (2018). Green synthesis, characterization and antimicrobial activity of zinc oxide quantum dots using *Eclipta alba* . Mater. Chem. Phys. 203, 40–48. 10.1016/j.matchemphys.2017.09.049

[B81] Singh A. K. Singh V. K. Singh M. Singh P. Khadim S. R. Singh U. (2019). One pot hydrothermal synthesis of fluorescent NP-carbon dots derived from *Dunaliella salina* biomass and its application in on-off sensing of Hg (II), Cr (VI) and live cell imaging. J. Photochem. Photobiol. A Chem. 376, 63–72. 10.1016/j.jphotochem.2019.02.023

[B82] Song T. Zhu X. Zhou S. Yang G. Gan W. Yuan Q. (2015). DNA derived fluorescent bio-dots for sensitive detection of mercury and silver ions in aqueous solution. Appl. Surf. Sci. 347, 505–513. 10.1016/j.apsusc.2015.04.143

[B83] Sunitha A. P. Sandeep K. Rose J. Hajara P. Saji K. J. (2021). Carbon quantum dots synthesized from *Plectranthus amboinicus*: an eco-friendly material with excellent non-linear optical properties. Mater. Today Proc. 47, 1601–1604. 10.1016/j.matpr.2021.04.288

[B84] Suresh U. Subhadra S. Sivaramakrishnan S. (2023). Green hydrothermal synthesis of carbon dot-silver nanocomposite from *Chondrococcus hornemanni* (marine algae): an application of mosquitocidal, anti-bacterial, and anti-cancer (MDA-MB-231 cells). Biomass Convers. biorefin. 14, 21461–21474. 10.1007/s13399-023-04214-9

[B85] Tade R. S. Patil P. O. (2020). Green synthesis of fluorescent graphene quantum dots and its application in selective curcumin detection. Curr. Appl. Phys. 20 (11), 1226–1236. 10.1016/j.cap.2020.08.006

[B86] Tak K. Sharma R. Dave V. Jain S. Sharma S. (2020). *Clitoria ternatea* mediated synthesis of graphene quantum dots for the treatment of Alzheimer’s disease. ACS Chem. Neurosci. 11 (22), 3741–3748. 10.1021/acschemneuro.0c00273 33119989

[B87] Tang L. Ji R. Cao X. Lin J. Jiang H. Li X. (2012). Deep ultraviolet photoluminescence of water-soluble self-passivated graphene quantum dots. ACS Nano 6 (6), 5102–5110. 10.1021/nn300760g 22559247

[B88] Thambiraj S. Shankaran R. (2016). Green synthesis of highly fluorescent carbon quantum dots from sugarcane bagasse pulp. Appl. Surf. Sci. 390, 435–443. 10.1016/j.apsusc.2016.08.106

[B89] Tungare K. Bhori M. Racherla K. S. Sawant S. (2020). Synthesis, characterization and biocompatibility studies of carbon quantum dots from *Phoenix dactylifera* . 3 Biotech. 10 (12), 540. 10.1007/s13205-020-02518-5 PMC7674538 33240743

[B90] Uprety B. Abrahamse H. (2022). Semiconductor quantum dots for photodynamic therapy: recent advances. Front. Chem. 10, 946574. 10.3389/fchem.2022.946574 36034651 PMC9405672

[B91] Vandarkuzhali S. A. A. Jeyalakshmi V. Sivaraman G. Singaravadivel S. Krishnamurthy K. R. Viswanathan B. (2017). Highly fluorescent carbon dots from pseudo-stem of banana plant: applications as nanosensor and bio-imaging agents. Sens. Actuators B Chem. 252, 894–900. 10.1016/j.snb.2017.06.088

[B92] Wang S. Yu J. Zhao P. Guo S. Han S. (2021). One-step synthesis of water-soluble CdS quantum dots for silver-ion detection. ACS Omega 6 (10), 7139–7146. 10.1021/acsomega.1c00162 33748627 PMC7970548

[B93] Wang X. Chen Q. Zhang Z. He H. Ma X. Liu Z. (2019). Novel *Enteromorpha prolifera* based carbon dots: probing the radical scavenging of natural phenolic compounds. Colloids Surf. B Biointerfaces 174, 161–167. 10.1016/j.colsurfb.2018.11.019 30448713

[B94] Xu H. V. Zheng X. T. Zhao Y. Tan Y. N. (2018a). Uncovering the design principle of amino acid-derived photoluminescent biodots with tailor-made structure–properties and applications for cellular bioimaging. ACS Appl. Mater. Interfaces 10 (23), 19881–19888. 10.1021/acsami.8b04864 29786414

[B95] Xu Q. Su R. Chen Y. Sreenivasan S. T. Li N. Zheng X. (2018b). Metal charge transfer doped carbon dots with reversibly switchable, ultra-high quantum yield photoluminescence. ACS Appl. Nano Mater. 1 (4), 1886–1893. 10.1021/acsanm.8b00277

[B96] Xu Y. Li D. Liu M. Niu F. Liu J. Wang E. (2017). Enhanced-quantum yield sulfur/nitrogen co-doped fluorescent carbon nanodots produced from biomass *Enteromorpha prolifera*: synthesis, posttreatment, applications and mechanism study. Sci. Rep. 7, 4499. 10.1038/s41598-017-04754-x 28674396 PMC5495774

[B97] Zaib M. Akhtar A. Maqsood F. Shahzadi T. (2021). Green synthesis of carbon dots and their application as photocatalyst in dye degradation studies. Arab. J. Sci. Eng. 46, 437–446. 10.1007/s13369-020-04904-w

[B98] Zhang C. Xiao Y. Ma Y. Li B. Liu Z. Lu C. (2017). Algae biomass as a precursor for synthesis of nitrogen-and sulfur-co-doped carbon dots: a better probe in *Arabidopsis* guard cells and root tissues. J. Photochem. Photobiol. B. 174, 315–322. 10.1016/j.jphotobiol.2017.06.024 28818777

[B99] Zhang J. Xia A. Chen H. Nizami A. S. Huang Y. Zhu X. (2022a). Biobased carbon dots production via hydrothermal conversion of microalgae *Chlorella pyrenoidosa* . Sci. Total Environ. 839, 156144. 10.1016/j.scitotenv.2022.156144 35609698

[B100] Zhang Z. Chen J. Yang Q. Lan K. Yan Z. Chen J. (2018). Eco-friendly intracellular microalgae synthesis of fluorescent CdSe QDs as a sensitive nanoprobe for determination of imatinib. Sens. Actuators B Chem. 263, 625–633. 10.1016/j.snb.2018.02.169

[B101] Zhou Q. Liu Y. Wu Y. Li Z. Li Y. Liu M. (2021). Measurement of mercury with highly selective fluorescent chemoprobe by carbon dots and silver nanoparticles. Chemosphere 274, 129959. 10.1016/j.chemosphere.2021.129959 33979911

[B102] Zhu H. Wang X. Li Y. Wang Z. Yang F. Yang X. (2009). Microwave synthesis of fluorescent carbon nanoparticles with electrochemiluminescence properties. Chem. Commun. (34), 5118–5120. 10.1039/b907612c 20448965

[B103] Zhu P. Liu Y. Tang Y. Zhu S. Liu X. Yin L. (2024). Bi-doped carbon quantum dots functionalized liposomes with fluorescence visualization imaging for tumor diagnosis and treatment. Chin. Chem. Lett. 35 (4), 108689. 10.1016/j.cclet.2023.108689

[B104] Zhu P. Zhao X. Zhu Q. Han X. Tang Y. Liao S. (2023). Exploring multi-element co-doped carbon dots as dual-mode probes for fluorescence/CT imaging. Chem. Eng. J. 470, 144042. 10.1016/j.cej.2023.144042

